# The Purple Sea Urchin *Strongylocentrotus purpuratus* Demonstrates a Compartmentalization of Gut Bacterial Microbiota, Predictive Functional Attributes, and Taxonomic Co-Occurrence

**DOI:** 10.3390/microorganisms7020035

**Published:** 2019-01-26

**Authors:** Joseph A. Hakim, Julie B. Schram, Aaron W. E. Galloway, Casey D. Morrow, Michael R. Crowley, Stephen A. Watts, Asim K. Bej

**Affiliations:** 1 Department of Biology, University of Alabama at Birmingham, 1300 University Blvd., Birmingham, AL 35294, USA; sawatts@uab.edu; 2 Oregon Institute of Marine Biology, University of Oregon, 63466 Boat Basin Rd, Charleston, OR 97420, USA; jschram@uoregon.edu (J.B.S.); agallow3@uoregon.edu (A.W.E.G.); 3 Department of Cell, Developmental and Integrative Biology, University of Alabama at Birmingham, 1918 University Blvd., Birmingham, AL 35294, USA; caseym@uab.edu; 4 Department of Genetics, Heflin Center Genomics Core, School of Medicine, University of Alabama at Birmingham, 705 South 20th Street, Birmingham, AL 35294, USA; mcrowley@uab.edu

**Keywords:** Illumina, high-throughput sequencing (HTS), bacteriome, PICRUSt, 16S rRNA gene, PhyloToAST, CoNet, QIIME, LEfSe, KEGG

## Abstract

The sea urchin *Strongylocentrotus purpuratus* (order Camarodonta, family Strongylocentrotidae) can be found dominating low intertidal pool biomass on the southern coast of Oregon, USA. In this case study, three adult sea urchins were collected from their shared intertidal pool, and the bacteriome of their pharynx, gut tissue, and gut digesta, including their tide pool water and algae, was determined using targeted high-throughput sequencing (HTS) of the 16S rRNA genes and bioinformatics tools. Overall, the gut tissue demonstrated *Arcobacter* and *Sulfurimonas* (Epsilonproteobacteria) to be abundant, whereas the gut digesta was dominated by *Psychromonas* (Gammaproteobacteria), *Propionigenium* (Fusobacteria), and Flavobacteriales (Bacteroidetes). Alpha and beta diversity analyses indicated low species richness and distinct microbial communities comprising the gut tissue and digesta, while the pharynx tissue had higher richness, more closely resembling the water microbiota. Predicted functional profiles showed Kyoto Encyclopedia of Genes and Genomes (KEGG) Level-2 categories of energy metabolism, membrane transport, cell motility, and signal transduction in the gut tissue, and the gut digesta represented amino acid, carbohydrate, vitamin and cofactor metabolisms, and replication and repair. Co-occurrence network analysis showed the potential relationships and key taxa, such as the highly abundant *Arcobacter* and *Propionigenium*, influencing population patterns and taxonomic organization between the gut tissue and digesta. These results demonstrate a trend of microbial community integration, allocation, predicted metabolic roles, and taxonomic co-occurrence patterns in the *S. purpuratus* gut ecosystem.

## 1. Introduction

The purple sea urchin *Strongylocentrotus purpuratus* (order Camarodonta, family Strongylocentrotidae) inhabits the rocky tide pools along the North-East Pacific from Alaska to Baja Mexico. *S. purpuratus* is primarily herbivorous, which tempers the growth of marine vegetation and plays an important role in shaping the dynamic population patterns in their marine ecosystem [1,2,3,4,5,6]. The low intertidal tide pools on the southern Oregon coast are dominated by *S. purpuratus* and are interspersed with mosaics of tufted algae and invertebrate assemblages representing multiple phyla [7]. The microhabitats of these tide pools are influenced by the feeding activity of the inhabiting sea urchins [7]. The sea urchins present unique digestive physiology in a straightforward model and offer an evolutionary context to fundamental biological and physiological processes occurring in higher deuterostome organisms [8]. In general, the pharynx is enclosed within the Aristotle’s Lantern, which is a pentamerally symmetric mastication apparatus of five tooth-like structures that assist in scraping and releasing intracellular nutrients from algae [9]. The pharynx tissue contains specialized mucus cells that contribute to the formation of a mucous envelope of ingested feed [10], forming individual pellets of gut digesta [11]. This gut digesta pellet formation has been considered an evolved digestive strategy of this organism, likely as a result of water flow dynamics in the gut lumen environment [12]. The role of gut bacteria in host health and digestion have been of interest beginning with the work of Lasker and Giese [9], who isolated gut bacteria from the gut digesta of *S. purpuratus*, showing the potential for these bacteria to digest polysaccharides from algal sources. In a separate study, bacteria isolated from the sea urchins *S. intermedius* and *S. nudus* demonstrated a similar algynolytic activity [13]. The importance of gut bacteria in sea urchin host health was further supported in *S. droebachiensis*, in which microbial suppression through antibiotics showed a reduced capacity for host incorporation of essential amino acids [14]. 

The microbial communities of the sea urchin gut pellets also play an important role in the biogeochemical cycles of the marine environment. In previous studies, it has been shown that the microbial community composition and their metabolic processes in the gut digesta remains stable following egestion into the environment [15,16,17]. For example, studies examining the chemical composition of *S. droebachiensis* egesta through flash combustion have shown increased in lipid, nitrogen, and organic carbon, and decreases in the carbon: nitrogen ratio, indicating the metabolic importance of the bacterial communities in the degradation and transformation of the contents within the pellets into a nutrient-rich food source for nearby marine organisms [17,18,19,20]. Additionally, it has been suggested that urchin gut microbiota are responsible for differences in algal digestion and synthesis of essential long chain fatty acids in both *S. purpuratus* and *S. droebachiensis* [20]. The pelleted egesta has also been considered as a mode for the dispersion of sea urchin gut microbiota into their environment [17,21]. Most of the early studies of the potential role of microbial communities in digestive processes of sea urchin gut ecosystem were conducted by culture-dependent methods [9]. However, recent advancement of the culture-independent method of high-throughput sequencing (HTS) of 16S rRNA genes from the metacommunity DNA has been shown to provide gut microbial community composition with high taxonomic coverage, including their potential metabolic functions [22,23]. Recently, the application of HTS on the V4 hypervariable segment of the 16S rRNA gene of the gut bacteriome of *Lytechinus variegatus* from the U.S. Gulf of Mexico [15,16,24] demonstrated distinct microbial community compositions between the gut tissue and gut digesta. Specifically, representative taxa from class Epsilonproteobacteria (assigned as *Arcobacter*/*Sulfuricurvum* through the National Center for Biotechnology Information (NCBI) Basic Local Alignment Search Tool (BLAST)) dominated the gut tissue, whereas Gammaproteobacteria (namely *Vibrio*) were heightened in the digesta [15]. Additionally, predictive functional profiling of these compartmentalized microbial communities showed energy metabolisms such as oxidative phosphorylation, carbon fixation, nitrogen, methane, and sulfur metabolisms to be heightened in the gut tissue, compared to carbohydrate, amino acid, and lipid metabolisms in the digesta [16]. 

Such HTS technology and bioinformatics analyses applied to the gut ecosystem of the naturally occurring sea urchin *S. purpuratus* can help establish a comprehensive microbial community composition and provide crucial information into the gut bacterial taxa and likely functions performed as they relate to host health and digestion. In this study, we have elaborated the microbial profiles of the gut tissue, pharynx tissue, and mucous enveloped gut digesta of *S. purpuratus* collected from their natural rocky tide pool habitat on the coastline of Oregon, USA. In addition, samples of the tide pool seawater and adjacent algal community were collected and also analyzed for comparison with the gut tissue and digesta. We used HTS and bioinformatics tools to analyze the community composition, patterns of microbial taxa allocation in the gut environment, and the predicted metabolic functions of the bacterial microbiota in the gut ecosystem. These data were further refined using Phylogenetic Tools for Analysis of Species-level Taxa (PhyloToAST v1.4.0) [25] alongside Quantitative Insights into Microbial Ecology (QIIME v1.9.1) [26] to condense redundant taxonomic groups. This allowed increased resolution of microbial taxonomic groups to the species level and enhanced beta diversity inference. Additionally, the keystone taxa (herein “key” taxa) of the gut ecosystem were elaborated through topological analysis of Co-occurrence Network inferences (CoNet v1.1.1) [27,28,29] based on criteria described in Berry and Widder [30,31]. The results of this baseline case study demonstrate the microbial composition and associated functional capacity within the compartmentalized gut system of this evolutionarily and ecologically significant purple *S. purpuratus* sea urchin species.

## 2. Materials and Methods 

### 2.1. Collection and Sample Preparation of S. Purpuratus 

**Figure 1:**
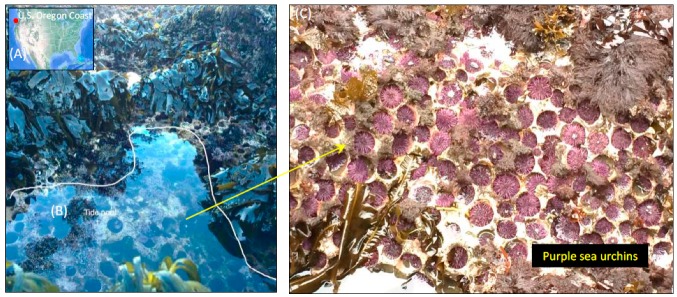
Sample collection site of *S. purpuratus* (purple sea urchins) from their natural rocky tide pool habitat along the coast of Oregon (43°18′14.3″N 124°24′05.1″W). (**A**) Satellite image of the collection site (red marker) provided through Google Earth Pro (v.7.3.2.5491) (Data SIO, NOAA, US Navy NGA, GEBCO, Image Landsat/Copernicus; US Dept. of State Geographer; image date: December 2015). (**B**) Overview of the tide pool collection site (labeled as tide pool 1) showing naturally occurring sea urchins. (**C**) Sea urchin congregates with the algal food source in view. Photographs by J.B Schram.

Adult *S. purpuratus* sea urchins (UR; *n* = 3) were collected from within the same natural rocky tide pool habitat at Cape Arago, Oregon (43°18′14.3″N 124°24′05.1″W) in September 2016, from within a 1 m^2^ sampling plot (Figure 1), under permit is: #20366 (Oregon Department of Fish and Wildlife). Sea urchins were measured and sexed, which was followed by tissue dissection performed at the Oregon Institute of Marine Biology (OIMB) in Coos County, Oregon. For each sea urchin, an incision was made into the test area surrounding the Aristotle’s Lantern mastication structure using sterilized instruments, and the test was cut radially to expose the internal digestive tissue. The pharynx, which was enclosed by the Aristotle’s Lantern, was separated from the gut tissue and collected. The remaining digestive tissue (gut tissue) was gently rinsed with sterile phosphate buffered saline water (1x PBS, pH 7.4) (Fisher Scientific, Hampton, NH, USA), and the contents (gut digesta) were collected. The whole gut tissue was collected separately from the voided gut digesta. Replicate seawater samples (water; *n* = 3) (1 L) from each tide pool was vacuum filtered separately through 0.22 µm filter paper (EMD Millipore Corporation, Danvers, MA, USA). The grazed-upon algal communities (algae; *n* = 3) immediately surrounding the sea urchins were also collected as the general food source and used in this study. A total of 15 samples (pharynx, *n* = 3; gut tissue, *n* = 3; gut digesta, *n* = 3; water, *n* = 3; and algae, *n* = 3) were placed into 95% (v/v) ethanol [32], flash frozen in liquid nitrogen, and shipped to the University of Alabama at Birmingham (UAB), where they were preserved at −20 °C until used. Research performed under the Institutional Animal Care and Use Committee (IACUC-10043). 

### 2.2. Community DNA Extraction, Illumina MiSeq Sample Preparation, and High-Throughput Sequencing 

The metacommunity DNA from each sample was purified using the Fecal DNA isolation kit (Zymo Research, Irvine, CA, USA; catalog no. D6010), and an amplicon library of the metacommunity V4 hypervariable region (V4) of the 16S rRNA gene was created using uniquely barcoded DNA oligonucleotide primers adapted from the Earth Microbiome Project (www.earthmicrobiome.org) [33,34,35]. These primers consisted of the upstream nucleotide sequence for hybridization to the Illumina MiSeq flow-cell surface (underlined), a “pad” region (italicized), and a “linker” region (bolded). The forward primer (515F) for the V4 segment was: 5′-AATGATACGGCGACCACCGAGATCTACAC*TATGGTAAT*TGTGTGCCAGCMGCCGCGGTAA-3′. The reverse primer for the V4 segment (modified from 806R) also included a unique barcode (6 N’s) region and was as follows: 5′-CAAGAGAAGACGGCATACGAGATNNNNNN*AGTCAGTCAG*CCGGACTACHVGGGTWTCTAAT-3′ (Eurofins Genomics, Inc., Huntsville, AL, USA) [35,36]. Polymerase chain reaction (PCR) amplification was performed using the LongAmp Taq PCR Kit (New England Biolabs, Ipswich, MA, USA; catalog no. E5200S) at a total reaction volume of 50 μL with the following reagents: 10 μL of 5× Reaction Buffer; 1.5 μL of each dNTPs (200 μM); 2 μL of each oligonucleotide primer (1.5 μM); 1.5 μL of LongAmp® enzyme (5 U); 30 μL of template DNA (2–5 ng/μL); and 3 μL of sterile H2O. The PCR proceeded with an initial denaturation at 94 °C for 1 min followed by 32 cycles of amplification of which each cycle consisted of denaturation at 94 °C for 30 sec, primer annealing at 50 °C for 1 min, and primer extension at 65 °C for 1 min, followed by the final extension at 65 °C for 3 min and a final hold at 4 °C. An amplicon fragment of approximately 380 bases was visualized through an ultraviolet (UV) transilluminator (Photodyne, Inc., Los Angeles, CA, USA) and excised with a sterile scalpel following electrophoresis through a 1.0% (w/v) Tris-borate-EDTA (TBE)/agarose gel [37]. The excised DNA fragments were purified using the QIAquick Gel Extraction Kit (Qiagen Inc., Venlo, Limburg; catalog no. 28704). PicoGreen dye (Life Technologies, Grand Island, NY, USA) was used to quantify each sample to adjust the concentration to 4 nM [35]. HTS was performed using the Illumina MiSeq platform [35,36], incorporating the 250 base paired-end kits from Illumina specific to the V4 region of the 16S rRNA gene. 

### 2.3. Quality Assessment and Filtering 

The raw sequence reads generated by HTS on the Illumina MiSeq platform were demultiplexed and converted to FASTQ format [38]. The read quality was evaluated using FastQC [39], and quality reads with 80% of bases at Q score >33 were retained for downstream analysis using the “fastx_trimmer” command from the FASTX Toolkit [35,40]. Then, the paired-ends were merged using USEARCH [41], and pairs with <50 base overlap and/or over 20 mismatching nucleotides were filtered. Read quality was again assessed after filtering using FASTQC, chimeric sequences were identified and removed using USEARCH [41]. Additionally, with the newly established bioinformatics techniques presented in the QIIME2 package (v2018.11) [42], an alternative approach to filtering and merging the paired-end sequence data was implemented. To do this, a “denoising” strategy was used based on the Poisson distribution through the Divisive Amplicon Denoising Algorithm program (DADA2, v1.10) [43,44]. This was performed utilizing the “qiime dada2 denoise-paired” module on the demultiplexed sequence data, with a truncation set at 250 bases for the forward and reverse reads. The sequence reads corresponding to each sample of this study have been deposited in NCBI SRA for public access (Bioproject number PRJNA504890).

### 2.4. Taxonomic Distribution

The resultant quality assessed sequence files were processed using QIIME (v1.9.1) [26] along with PhylotoAST (v1.4.0) [25] to condense redundant operational taxonomic units (OTUs) [45]. First, OTUs were selected at a 97% sequence similarity threshold using the default UCLUST algorithm option in QIIME (v1.9.1) [41]. Representative OTU sequences were then selected using the “most_abundant” option, and taxonomy was assigned to the representative sequences at a 60% confidence threshold using the Ribosomal Database Project (RDP) classifier [46], trained with the GreenGenes reference database (v13.8) [47,48]. At this stage, OTUs occurring at less than 0.0005% average abundance across all samples in the study were filtered [49,50,51,52,53]. Then by using the PhyloToAST (v1.4.0) workflow, the species-level resolution was enhanced using the “assign_taxonomy_by_blast _result.py” command to assign taxonomy through BLAST [45] to the GreenGenes (v13.8) database, and redundant OTUs were merged through the “condense_workflow.py” command [25]. Variation in the read-depth was accounted for by subsampling of the condensed OTU table using both the median and minimum read count values across all samples as described in de Carcer et al. [54] through the “single_rarefaction.py” command in QIIME (v1.9.1), and both subsampled OTU tables were assessed for downstream analysis. Additionally, for the top 100 taxa determined in the rarefied OTU table, the representative sequences were extracted and aligned to multiple databases using the SILVA ACT: Alignment, Classification and Tree Service (www.arb-silva.de/aligner) [55]. For this analysis, the SSU (Small Sub-Unit) category was selected, and a minimum similarity identity was set to 0.9, with 20 neighbors per query sequence. Sequences below an identity threshold of 70% were discarded. For taxonomic identification, the least common ancestor (LCA) method was used, and the databases selected included GreenGenes [47,48], Ribosomal Database Project (RDP) [56], and SILVA [57]. Lastly, for the alternative QIIME2 (v2018.11) method, the denoised and merged sequence data was used to generate representative sequences with the “qiime feature-table tabulate-seqs” command. These representative sequences, herein referred as amplicon sequence variants (ASVs), were assigned taxonomic identities through the “qiime feature-classifier” command utilizing the “classify-sklearn” option [58] against the GreenGenes (v13.8) database. 

### 2.5. Alpha Diversity 

The PhyloToAST (v1.4.0) condensed and minimum-count subsampled OTU table (herein, rarefied OTU table) was used to determine the taxonomic distribution and alpha diversity metrics of each sample. The rarefied OTU table was merged according to biological replicates and used to create the relative abundance graph of phyla represented at >1% abundant, as well as the top 100 most resolved taxonomic identities across all sample groups using Microsoft Excel Software (Seattle, WA, USA). Taxa represented at >1% in the gut system (gut tissue and digesta) were also visualized, with standard deviations calculated through STAMP (v2.1.3) [59]. Shannon [60,61,62], and Simpson [61,63] diversity measurements were determined through the “alpha_diversity.py” command in QIIME (v1.9.1). These values were plotted as a kernel density estimator-smoothed histogram using the “diversity.py” command through PhyloToAST (v1.4.0), to show both the diversity value and the range of underlying data points (density) for each sample group. Kruskal–Wallis H-tests were performed for the five groups to show the alpha diversity variation between groups at a significance value of *p* = 0.1 [64].

### 2.6. Beta Diversity 

For beta diversity, the rarefied OTU table was used to determine the Bray–Curtis distance matrix values [65]. These values were also used to calculate significant grouping among biological replicates (*n* = 3) through an analysis of similarity (ANOSIM) and multivariate analysis of variance (Adonis) of groups, both set at 999 permutations, determined through the QIIME (v.1.9.1) “compare_categories.py” module utilizing the Vegan (v2.4.3) R package implementation of the statistical methods [66,67,68]. Additionally, this ANOSIM and Adonis analysis was performed on each OTU table generated in this study (5 total), which included the unfiltered OTU table, filtered OTU table (<0.0005%), PhyloToAST (v1.4.0) condensed OTU table, condensed median, and condensed minimum subsampled OTU tables. Visualization of beta diversity trends was performed using Plymouth Routines in Multivariate Ecological Research (PRIMER-6) software (Primer-E Ltd, Plymouth Marine Laboratory, Plymouth UK, v6.1.2) [67]. In PRIMER-6, a 2D multidimensional scale (MDS) plot was generated using the Bray–Curtis distance matrices, to show variation between each sample, along with an overlay of Bray–Curtis similarity values [69]. A dendrogram was also generated based on clustering by group average [69]. A 2D MDS and dendrogram cluster analysis was also performed on the top 100 OTUs and the remaining rare OTUs, to show the contributions of both the heightened and rare taxa to the observed sample community diversity and cluster patterns. In addition to the Bray–Curtis based analyses described above, the rarefied OTU table was used to determine the weighted and unweighted Unifrac distances [70], which was calculated through the “beta_diversity_through_plots.py” module of QIIME (v1.9.1), and used to generate the 3D principle coordinates analysis (PCoA) plots through the “PCoA.py” command in PhyloToAST (v1.4.0). These values were also used to calculate the ANOSIM and Adonis metrics for group analyses as previously described, and uploaded into PRIMER-6 to generate the dendrogram based on group average. Heatmap analysis was performed using the rarefied OTU table in R (v3.3.2), incorporating the heatmap.2 function from gplots (v3.0.1) package [71]. In brief, the associated sample group dendrogram was created through the Vegan (v2.4.3) package [68] using the Bray–Curtis distance metric of the grouped biological replicate count data and clustered according to the group average algorithm. Microbial taxa represented at <1% of the total dataset were filtered from the heatmap. A color palette was selected using the RColorBrewer package [72], and the relative abundances were shown for each taxon across all sample groups (black bar lines). Linear discriminant analysis (LDA) effect size (LEfSe) analysis was used to determine the taxa contributing to the effect size between the compartmentalized gut microbial communities of the gut tissue (*n* = 3) and digesta (*n* = 3) [73]. This analysis was performed through the Hutlab Galaxy web application (huttenhower.sph.harvard.edu/galaxy/), and incorporated the non-parametric Kruskal-Wallis sum-rank test for significant differential abundance set at a significance of *p* = 0.05 [64], followed by LDA to estimate effect size at log(10) values [73,74]. The results were plotted to show those taxa that demonstrated an LDA of ±3 for effect size. 

### 2.7. Predicted Functional Analysis 

The functional capacity associated with the microbial communities of the gut tissue and digesta was determined using the Phylogenetic Investigation of Communities by Reconstruction of Unobserved States (PICRUSt v1.1.2) package [75] and analyzed in STAMP (v2.1.3) [59]. For this analysis, an OTU table was constructed by the “pick_closed_reference_otus.py” strategy to ensure representative taxonomic information through the GreenGenes (v13.8) database as suggested in PICRUSt [47,48,75]. The resultant OTU table was normalized by copy number, and Kyoto Encyclopedia of Genes and Genomes (KEGG) Orthology (KO) Ids were predicted along with the weighted Nearest Sequenced Taxon Index (NSTI) values for the confidence of predictions using the “predict_metagenomes.py” command. The assigned functional categories were then collapsed into levels (KEGG-Level-2, and 3) through the “categorize_by_function.py” command. The KEGG-Level-2 and 3 profiles were uploaded into STAMP (v2.1.3) for two-group scatter plot analysis, to determine the metabolic categories that are preferentially enriched in each group at two levels of hierarchical classification. Additionally, LEfSe analysis [73] was performed on the KO Ids using an LDA score of ± 2.4, again utilizing the non-parametric Kruskal–Wallis sum-rank test for significant differential abundance set at a significance of *p* = 0.05 [64] and LDA for effect size using log(10) values [73,74], to demonstrate those categories contributing most to functional profile dissimilarity.

### 2.8. Co-Occurrence Analysis of Microbial Taxa 

Significant co-occurrence patterns occurring between the microbial communities of the gut tissue and the gut digesta were determined using Co-occurrence Network inference (CoNet v1.1.1) [27,28,29]. To do this, the rarefied OTU data were uploaded into Cytoscape (v3.6.0) [76] through the CoNet (v1.1.1) plugin with taxa assigned to sample type (gut tissue and gut digesta). Links between higher level taxa were not explored and a parent-child exclusion was applied. Taxonomic entries with a cumulative group sum of 200 and at least 2/3 of samples containing non-zero values were kept [27,28,29,77]. Significant co-occurrences between taxa were determined by utilizing the Pearson [78,79], Spearman [80], Bray–Curtis [65], Kullback–Leibler [81], and mutual information similarity [82], with a 10^−8^ pseudo-count [27,28,29,77]. The 200 highest (most positive) and lowest (most negative) edges were selected and merged by the union approach using the mean value [77]. The multi-edge scores were shuffled row-wise at 100 permutations (for null distributions), followed by bootstrapping at 100 permutations (for randomizations). The *p*-values of the multi-edges assigned to node pairs were merged using the Brown method [83], with unstable edges filtered out, and the corrected significance value (q-value) was determined with a threshold set at *p* < 0.05 for significance [27,28,29]. 

The final network was constructed in Cytoscape (v3.6.0) using the radial layout algorithm in the yFiles plugin (v1.0) [84], and topological parameters were determined by NetworkAnalyzer (v2.7) [85] using an undirected approach. Node sizes were scaled to their group abundance, colored according to phylum (class for Proteobacteria), and assigned a shape according to group membership (circle for gut tissue, “-gut”; diamond for gut digesta; “-dig”). The edges were scaled by the q-value and colored according to their positive (co-presence; green) and negative (co-exclusion; red) association. Based on the topological features determined through NetworkAnalyzer (v2.7), those nodes tending to have a high degree (number of edges), closeness centrality, and low betweenness centrality have been referred to as key taxa as described by Berry and Widder et al. [30] [77,86,87]. These features were plotted as a scatter plot (y = closeness centrality; x = betweenness centrality; node size is scaled to degree) through Microsoft Excel Software (Seattle, WA, USA). The top 10 nodes based on their closeness centrality values were selected as likely key taxa.

## 3. Results

### 3.1. Environmental Conditions and Sea Urchin Measurements

The tide pool location sea water conditions were determined to have a salinity of 30.19 ppt with a 7.7 pH. The dissolved oxygen content of the tide pool water was determined to be 59% and the temperature was determined to be 13.1 °C. The sea urchins of this study weighed between 38.98–53.35 g and had a mean diameter of 5.1–5.6 cm, a height of 2.6–2.8 cm, and a spheroid volume of 38.4–41.6 cm^3^. The sexes of the sea urchins for the study were determined as UR1 = F, UR2 = F, and UR3 = M. 

### 3.2. Quality Assessment and Sample Statistics

The total sequences generated through Illumina MiSeq-based HTS of the bacterial 16S rRNA gene of the 15 samples of the study generated a total of 1,714,746 forward and reverse reads (Table 1). Quality checking and trimming using the FASTX Toolkit, followed by merging of the forward and reverse sequences resulted in 1,249,827 total reads. Grouping of biological replicate data (*n* = 3) showed the following total sequence read counts: algae (340,438), gut digesta (221,684), gut tissue (231,854), pharynx (194,640), and water (261,211). Clustering of sequences into OTUs and taxa assignment revealed a total of 44,664 unique assignments. Filtering of rare OTUs occurring at less than 0.0005% reduced the number of OTUs to 4290 unique observations across all samples. Condensing of redundant taxonomic IDs through PhyloToAST (v1.4.0) showed a total of 776 OTUs. Rarefication of the condensed OTU table to the median read count value (77,806) and the minimum read count value (49,641) both maintained 776 unique observations. Through the alternative strategy utilizing DADA2 (v1.10) implemented in QIIME2 (v2018.11), a total of 1134 unique features were determined (ASVs) representing a total of 467,866 reads across all samples, which were subsequently assigned to 371 taxonomic identities when collapsed to the species level (data not shown). 

### 3.3. Taxonomic Distribution across Samples

**Figure 2:**
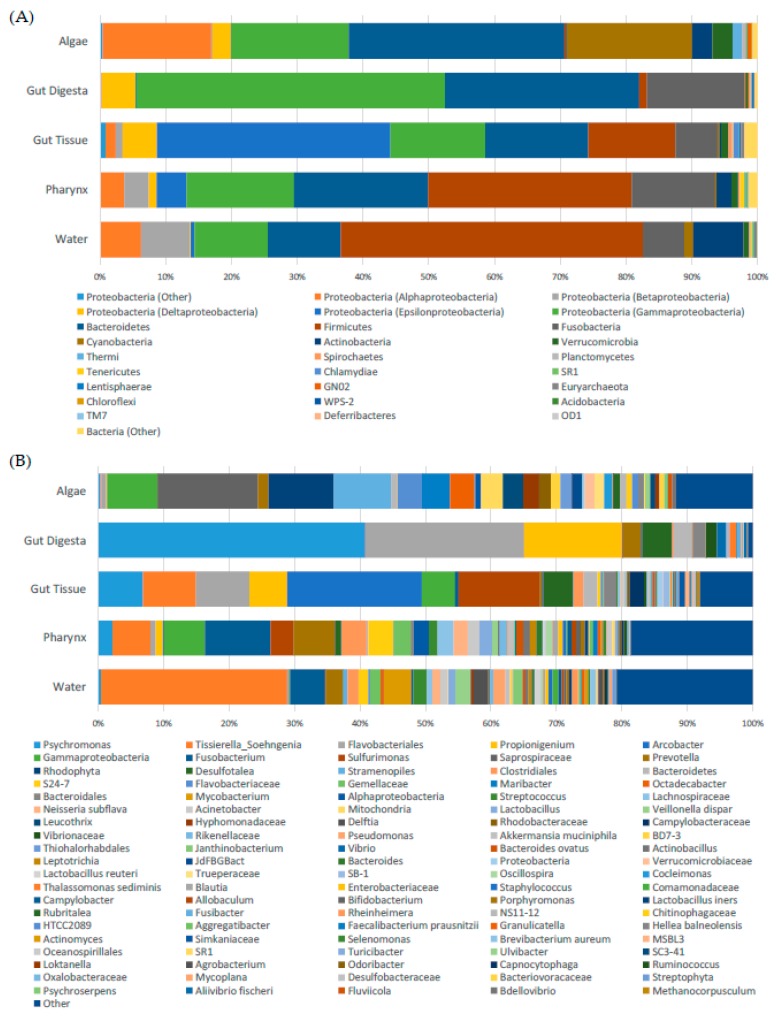
Taxonomic distribution of microbial communities in the gut ecosystem and rocky tide pool environment of the sea urchin *S. purpuratus*. (**A**) The relative abundance of phyla (class for Proteobacteria) represented at >1% are shown, with phyla <1% grouped as “Other.” (**B**) The top 100 taxa at the most resolvable level across all samples were also visualized, and taxa not included as the top 100 were assigned as “Other.” OTUs were picked at 97% similarity threshold, filtered at <0.0005%, condensed using PhyloToAST (v1.4.0), and subsampled to minimum OTU count (rarefied OTU table). Taxonomic identities were determined by using the GreenGenes (v13.8) database, and the color code corresponds to each taxon observed across the gut and environmental samples. Grouping of biological replicates (*n* = 3) was supported by an analysis of similarity (ANOSIM) and multivariate analysis of variance (Adonis) (*p* < 0.001). Relative abundance plot was created through Microsoft Excel Software (Seattle, WA, USA).

Taxonomic distribution across all samples showed the gut tissue represented a uniquely heightened amount of Epsilonproteobacteria in the order of Campylobacterales, namely family Camplylobacteraceae (*Arcobacter*) (~20%) and Helicobacteraceae (*Sulfurimonas*) (~12%) as compared to the other samples of the study (Figure 2A,B). Members of Firmicutes (*Tissierella_Soehngenia*) were observed in the gut tissue and appeared to be present in the pharynx tissue and the environmental samples, particularly the water. Also observed were Bacteroidetes (Flavobacteriales, ~2%), as well as Gammaproteobacteria (*Psychromonas*, ~7%), Deltaproteobacteria (*Desulfotalea*, ~5%), and Fusobacteria (*Propionigenium*, ~5%), to lesser degrees of abundance. 

**Figure 3:**
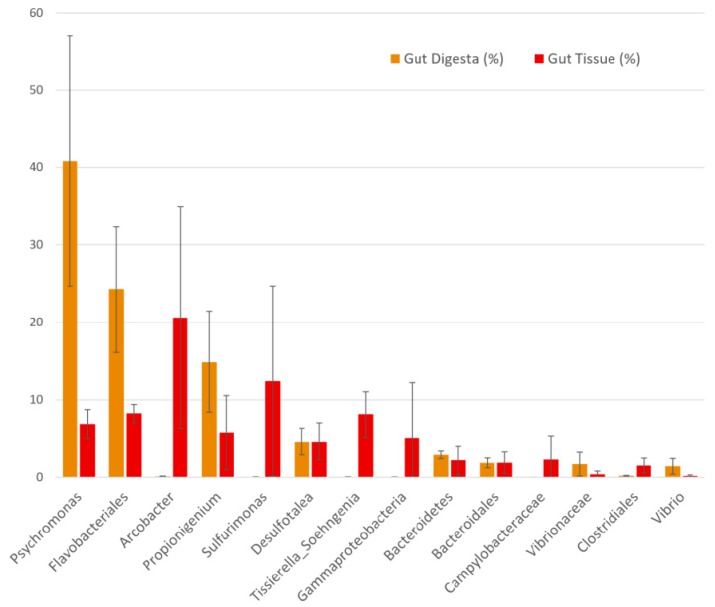
Comparison of the observed taxa between the gut tissue (*n* = 3) and gut digesta (*n* = 3) using the rarefied OTU table data. Taxa observed at <1% were filtered from the graph. Standard deviation and relative abundances were determined through STAMP (v2.1.3), and the graph was generated through Microsoft Excel Software (Seattle, WA, USA).

The dominant microbial taxa in the gut digesta were observed to be members of *Psychromonas* (~40%), *Propionigenium* (~15%), and class Flavobacteriales (~25%). Compared to the gut tissue, these taxa comprised a large relative abundance (~80%) of the bacterial microbiota observed in the gut digesta. The gut digesta also included members of phylum Bacteroidetes (3%), class Gammaproteobacteria identified as Vibrionaceae and *Vibrio* (~1 and ~2% respectively), and *Desulfotalea* (~5%) at noticeable relative abundances (Figure 3). 

The pharynx tissue presented many of the same bacterial taxa observed in the water and to a lesser extent the algae samples. Of these shared taxa, *Fusobacterium* (~10%) was observed at the highest abundance, which was followed by the families S27-7 (Bacteroidales) and Gemellaceae, and genus *Prevotella*. The presence of these bacteria in the gut tissue and gut digesta were negligible (<1%). However, the pharynx tissue also included members of *Tissierella_Soehngenia* (~6%), *Sulfurimonas* (~3%), and *Desulfotalea* (~1%) which were observed in the gut tissue and digesta. 

The algae samples showed Saprospiraceae (~15%), Rhodophyta (~10%), and *Stramenopiles* (~9%) to be heightened, the presence of which was negligible in the other samples in the present study. We also observed the genera *Maribacter* and *Octadecabacter* at equal capacities (~4%). All microbial taxa at their most resolved level, including their group abundance identified in the rarefied OTU table used in this study, have been elaborated (Table S1). Additionally, the unfiltered OTU table generated in this study has also been elaborated (Table S2).

From the alternative strategy utilizing ASVs, the taxonomic distribution was in concert with the OTU picking strategy, with only slight variations in relative abundance (Table S3). In the gut tissue, a slightly higher relative abundance of *Arcobacter* (~22%) and *Sulfurimonas* (~14%) was determined through the ASV method compared to the OTU picking strategy. A variation in relative abundances of heightened taxa was also observed in the gut digesta, where *Psychromonas* was more highly represented at ~50%, whereas *Propionigenium* (~13%) and Flavobacteriales (~14%) were marginally less abundant. The pharynx tissue was also consistent between the two strategies, with a slightly higher abundance of *Fusobacterium* (12%) and *Sulfurimonas* (~7%). The ASV method was able to resolve an extra phylogenetic level of one heightened feature in the pharynx tissue, which was classified to phylum Gammaproteobacteria (~6%) through the OTU picking method, but was determined as order Legionellales (~10%) through the ASV approach. For the water samples, *Tissierella_Soengenia* (~32%) comprised the highest abundance, followed by *Fusobacterium* (~7%), and for the algae samples, Saprospiraceae, Rhodophyta, and *Stramenopiles* were confirmed through the ASV method, at slightly higher relative abundances compared to the OTU picking method. Additionally, the results of the alignment of the representative sequences corresponding to the top 100 taxa determined in the rarefied OTU table through SILVA ACT: Alignment, Classification and Tree Service (www.arb-silva.de/aligner) have been elaborated (Table S4). 

### 3.4. Alpha Diversity

**Figure 4:**
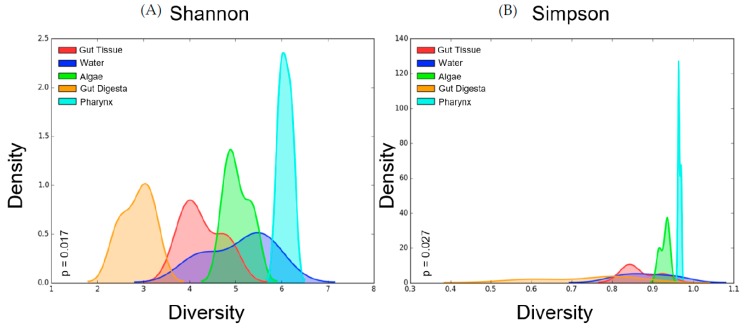
Per-group alpha diversity measurements calculated across all samples in the study. (**A**) Shannon and (**B**) Simpson alpha diversity histograms were smoothed by kernel density estimation. The Kruskal–Wallis H-tests were performed for the five groups and showed a significance value of *p* = 0.017 for the Shannon and *p* = 0.027 for Simpson diversity measurements, indicating significant differences between each group’s alpha diversity. The X-axis shows the diversity value of Shannon (values much greater than 0 are more diverse) and Simpson (values closer to 1 are more diverse). The histogram values of each sample were smoothed through kernel estimation to show the range of sample data points within each group. The Y-axis depicts the density function, which denotes the distribution of data points falling within this range (higher peak represents more clustered data points). Relevant p-values are listed in each graph. Plots were generated using the “diversity.py” command through PhyloToAST (v1.4.0).

Alpha diversity measures performed on the rarefied OTU table showed the highest Shannon diversity in the pharynx tissues (avg = 6.08 ± 0.08 SEM), followed by the water (5.08 ± 0.44 SEM) and algae (5.04 ± 0.17 SEM), with the gut tissue (avg = 4.27 ± 0.28 SEM) and gut digesta (avg = 2.87 ± 0.21 SEM) showing the lowest diversity. Simpson diversity showed a similar trend in the sea urchin gut samples, with the pharynx tissue showing the highest diversity (avg = 0.976 ± 0.002 SEM), and the gut tissue (0.871 ± 0.027 SEM) and gut digesta (0.738 ± 0.070 SEM) showing the lowest. The water (0.885 ± 0.039 SEM) and algae (0.929 ± 0.007 SEM) microbial profiles were more diverse than the gut tissue and digesta, but less diverse than the pharynx tissue (Table 1). Kruskal–Wallis H-test analysis through PhyloToAST (v1.4.0) showed significant differences between the Shannon (p = 0.017) and Simpson (p = 0.027) alpha diversity values across the 5 groups of the study. The kernel density smoothed histograms plotted using PhyloToAST (v1.4.0) visualized the alpha diversity values, with density representing the intra-sample variation in the group (high density = low variation between the diversity of samples in the group). For both Shannon (Figure 4A) and Simpson (Figure 4B) diversity, the highest density corresponded to the pharynx tissue samples, indicating low intra-sample variation in the group. Shannon diversity showed the broadest diversity value range in the water (Figure 4A), and Simpson showed the broadest range in the gut digesta (Figure 4B). For Shannon diversity, the gut digesta and gut tissue samples had distinct histogram peaks. For Simpson diversity, the range of alpha diversity measures for the gut digesta was broader, indicating a minimal density peak, and the gut tissue showed a low but noticeable peak (Figure 4A,B).

### 3.5. Beta Diversity

**Figure 5:**
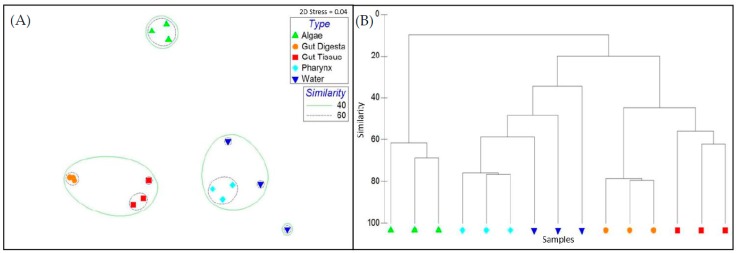
Beta diversity analysis of microbial communities observed across all samples in the study using Bray–Curtis similarity metrics determined for the rarefied OTU table. (**A**) A 2D multidimensional scale (MDS) plot analysis was performed to show sample cluster patterns based on observed OTUs, with a 40% and 60% Bray-Curtis similarity overlay, and the stress value (2D Stress = 0.04) was indicated. (**B**) Dendrogram analysis was also performed and each sample’s cluster patterns were based on group average. The OTU table was pretreated via standardization by the total and log transformation prior to Bray-Curtis analysis. Figure legends are shown in the 2D MDS plot. Data was generated and plotted through PRIMER-6 software (Primer-E Ltd, Plymouth Marine Laboratory, Plymouth UK, v6.1.2).

Microbial taxonomic distribution patterns determined through Bray–Curtis metrics across all samples revealed the gut tissues to cluster together at >50% and the gut digesta >60% (Figure 5A). These two sample groups maintained a Bray–Curtis similarity >40%. The pharynx tissues group demonstrated low intrasample variation (Bray–Curtis similarity >60%) and had a microbial community structure that was the most similar to the water samples (Bray–Curtis similarity >40%, Figure 5B). The algae samples clustered together at a value >60% but were least similar to the other samples of the study. The observed cluster patterns were strengthened when only the top 100 OTUs were plotted through 2D MDS (Figure S1A,B). For the rare taxa (the taxa not included in the top 100 OTUs), although low intrasample variation was observed, the gut tissue group was clustered nearer the pharynx and water samples (Figure S1C,D). Although the general trends of within- and between-group sample similarity were supported through the weighted (Figure S2A,B) and unweighted (Figure S3A,B) Unifrac 3D PCoA and dendrogram analysis, there were slight variations in cluster patterns between the two Unifrac approaches. Through the weighted Unifrac method, the gut tissue and gut digesta microbial communities clustered closer together as compared to the unweighted method, which showed the gut tissue samples to more closely resemble the pharynx. However, in concert with the Bray–Curtis method, both methods demonstrated the pharynx tissue to more closely resemble the water samples, and the algae samples maintained a divergent cluster pattern away from the other samples of the study.

ANSOIM and Adonis analysis supported biological replicate grouping and demonstrated the highest grouping similarity corresponding to the OTU data that was rarefied to the minimum count value (ANOSIM R = 0.94; Adonis R2 = 0.76). However, significant grouping patterns of biological replicate groups were observed when the analysis was performed on each OTU table generated in this study (unfiltered, filtered, condensed, median rarefied, and minimum rarefied OTU tables) with *p* = 0.001 for ANOSIM and Adonis (Table 2). Additionally, ANOSIM and Adonis performed on the rarefied OTU table using the weighted (ANOSIM R = 0.92; Adonis R2 = 0.77) and unweighted (ANOSIM R = 0.88; Adonis R2 = 0.73) Unifrac statistic also supported significant biological replicate grouping, with *p* = 0.001 determined using both methods, with the weighted method showing a slightly higher value for both statistics.

**Figure 6:**
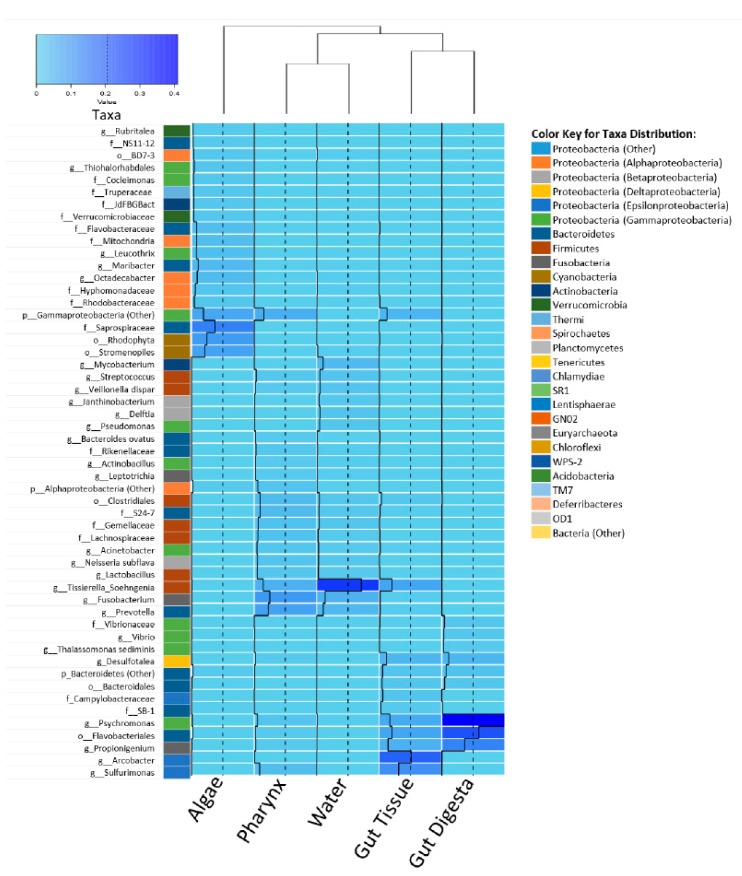
Heatmap of the top 53 taxa at the highest resolution, determined using the rarefied OTU table and generated using R (v3.3.2). The heatmap.2 function from the gplots (v3.0.1) package (www.rdocumentation.org/packages/gplots) was used. Sample dendrogram was generated using Vegan (v2.4.3), employing the Bray-Curtis metric of the grouped biological replicate count data. Color palette selected using the RColorBrewer package and set from “sky blue” for less abundant, to “blue” for more abundant (shown in color key). Relative abundance values of each taxon are also indicated through a trace line (black). The associated table includes the most resolvable taxonomic assignment according to the GreenGenes (v13.8) database, which is color-coded to the phylum level assignments (class for Proteobacteria) as indicated in the key and corresponding to the relative abundances in the Figure 2A relative abundance graphs. The figure has been generated using scalable graphics and, therefore, regions of interest can be viewed at a higher resolution digitally by increasing the magnification.

**Figure 7:**
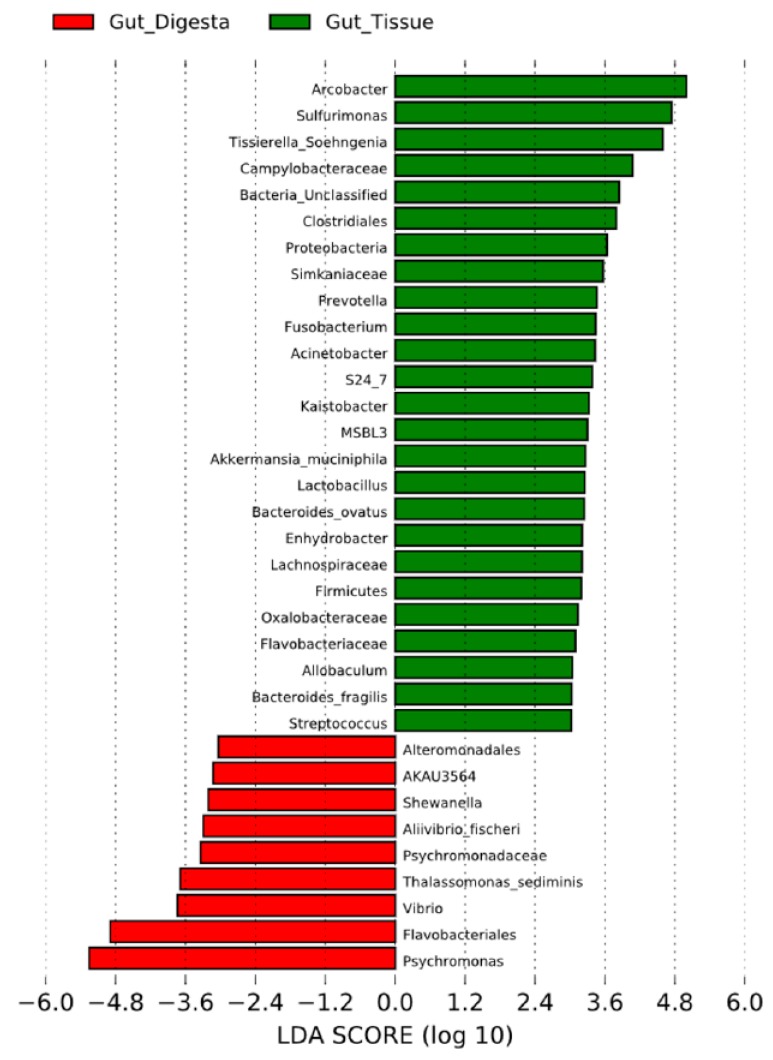
Linear discriminant analysis (LDA) effect size (LEfSe) performed on the microbial community relative abundance data at the of the gut tissue (*n* = 3) and gut digesta (*n* = 3). Grouped data were first analyzed using the Kruskal–Wallis test with a significance set to 0.05 to determine if the data was differentially distributed between groups, and those taxa that were differentially distributed were used for LDA model analysis to rank the relative abundance difference between groups. The LDA for significance was set to ±3, and the log(10) transformed score is shown to demonstrate the effect size. Data were analyzed and prepared through Hutlab Galaxy provided through the Huttenhower lab. The gut tissue group is shown as green, and the gut digesta group as red.

Similarity trends between sample groups were elaborated by heatmap analysis, which also depicted the relative abundance associated with each taxon contributing to the group diversity across the grouped biological replicate samples (Figure 6). Dendrogram analysis across grouped biological replicates for the heatmap analysis revealed the gut digesta and gut tissue to have a more similar microbial ecology composition, and likewise for the water and pharynx groups. The algal food source was the least similar to the other samples of the study, confirming the similarity trends observed in the 2D MDS plot analysis (Figure 5A). LEfSe analysis of the gut tissue and digesta at an LDA score of ±3 showed the taxa contributing most to the dissimilarity (effect size) of the gut tissue to be *Arcobacter*, *Sulfurimonas*, and *Tissierella_Soehngenia*, whereas the gut digesta revealed *Psychromonas*, Flavobacteriales and *Vibrio* (Figure 7). The effect size of *Propionigenium*, which was noticeably abundant in the gut tissue (5.7% ± 4.8%) and digesta (14.9% ± 9.6%), was not observed in LDA analysis, likely due to overlapping standard deviation values (as observed in Figure 3).

### 3.6. Predicted Functional Capacity

**Figure 8:**
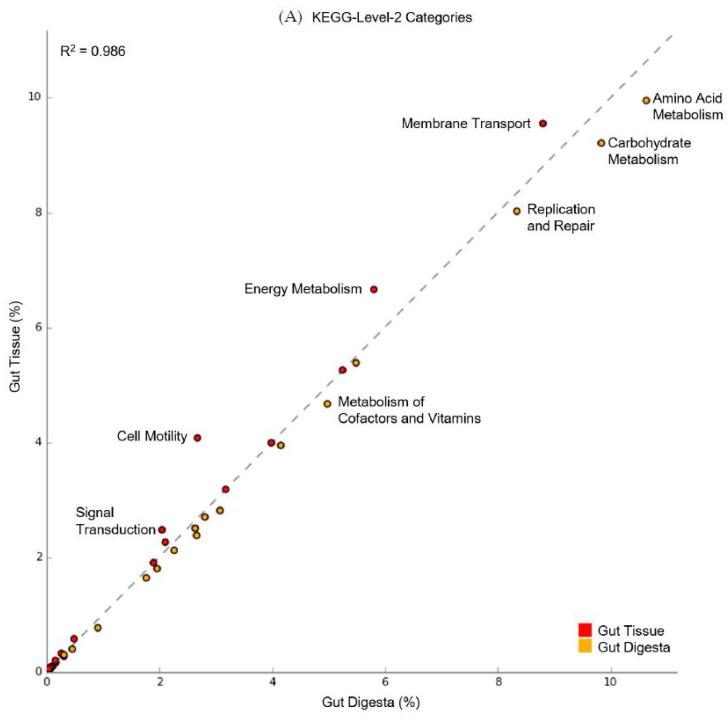

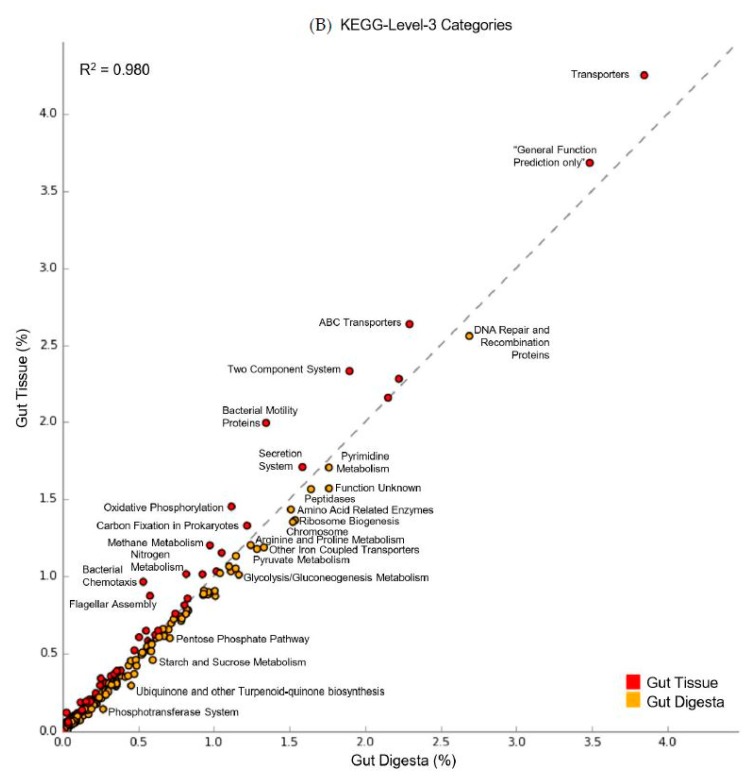
Scatter plot analysis of the predicted KEGG Orthology (KO) metabolic functions determined through Phylogenetic Investigation of Communities by Reconstruction of Unobserved States (PICRUSt v1.1.2) performed on the gut tissue (*n* = 3) and gut digesta (*n* = 3). Biological replicates were grouped, and analysis was performed for the (**A**) KEGG-Level-2 and (**B**) KEGG-Level-3 hierarchical functional categories. The linear regression value calculated for the two groups is shown for each scatter plot graph. Preferentially enriched categories for the gut tissue are shown as red, and for the gut digesta as brown. Those categories with clearly preferentially abundant categories have been labeled. Data were analyzed and visualized using STAMP (v2.1.3) analytical software. The node labels have been generated using scalable graphics, and therefore regions of interest can be viewed at a higher resolution digitally by increasing the magnification.

**Figure 9:**
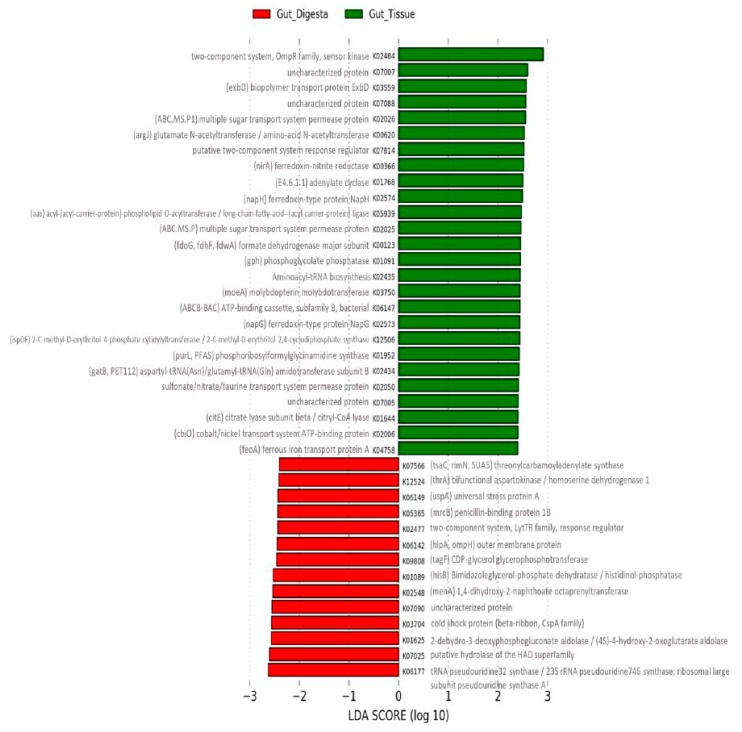
Linear discriminant analysis (LDA) effect size (LEfSe) performed on the KEGG Orthology (KO) metabolic functions determined through PICRUSt (v1.1.2) for the gut tissue (*n* = 3) and gut digesta (*n* = 3). The KO Ids were determined through the “predict_metagenomes.py” command in PICRUSt (v1.1.2). Grouped data were analyzed using the Kruskal–Wallis with a significance set to 0.05, and the significantly differentially distributed KO Ids were used for LDA model analysis ranking the relative abundance significance, at an LDA threshold showing entries ranking at ± 2.4. The log(10) transformed score is shown as the effect size. Data were analyzed and prepared through Hutlab Galaxy provided through the Huttenhower lab. The gut tissue group is shown as green, and the gut digesta group as red. The KO Id labels have been generated using scalable graphics, and therefore regions of interest can be viewed at a higher resolution digitally by increasing the magnification.

The predicted functional capacity of the microbial communities of the digestive system was performed using PICRUSt (v1.1.2), showing an average NSTI value of 0.139 (range from 0.105 - 0.179). Scatter plot analysis using STAMP (v2.1.3) of the KEGG-Level-2 categories showed a preferential abundance of energy metabolism in the gut tissue, as well as membrane transport, cell motility, and signal transduction (Figure 8A). For the gut digesta, amino acid metabolism, carbohydrate metabolism, metabolism of cofactors and vitamins, and replication and repair categories were observed. KEGG-Level-3 observances showed a preferential abundance of oxidative phosphorylation, carbon fixation, methane, and nitrogen metabolisms in the gut tissue, and categories related to the transporter and motility-related categories (2-component system, chemotaxis, bacterial motility proteins, and flagellar assembly) (Figure 8B). The gut digesta displayed categories related to pyrimidine metabolisms, as well as peptidases and amino acid enzymes, including arginine and proline metabolisms. Other categories that were enriched in the digesta included starch and sucrose metabolism, pentose phosphate pathway, glycolysis/gluconeogenesis, and ubiquinone and turpenoid-quinone biosynthesis necessary for electron transport. The KEGG-Level-2 and 3 categories identified through PICRUSt (v1.1.2) were listed in Table S5. Additionally, the LEfSe analysis of KO Ids (highest metabolic resolution) contributing most to the effect size difference between the gut tissue and gut digesta were determined and listed along with their associated metabolic definitions (Figure 9 and Table S6).

### 3.7. Co-Presence, Co-Exclusion, and Key Taxa in the Gut Environment

**Figure 10:**
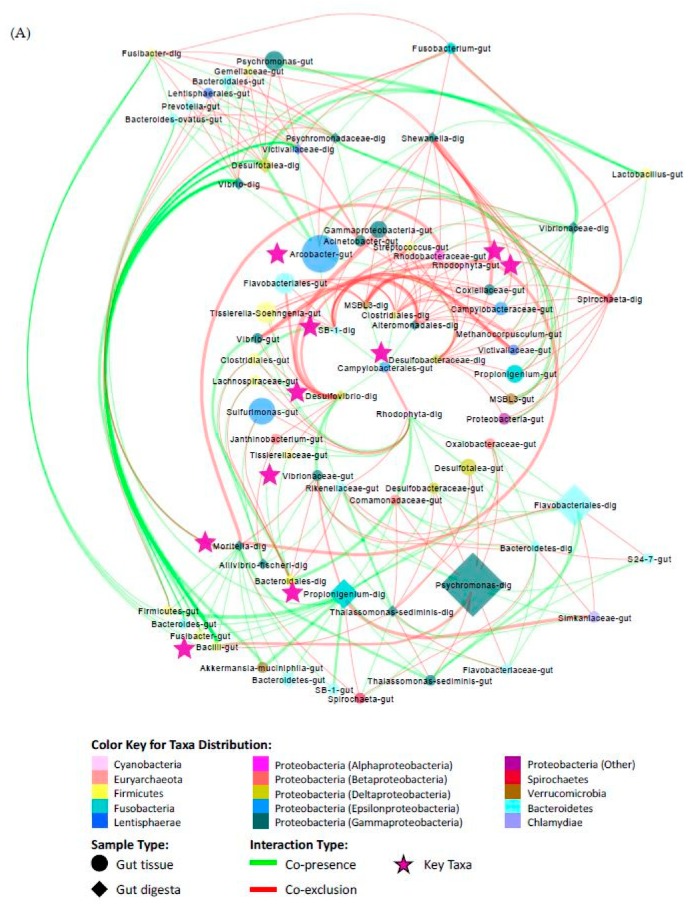

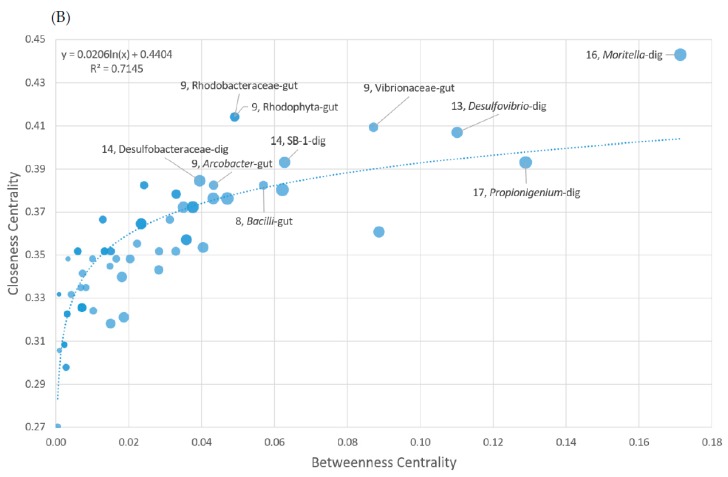
Co-occurrence patterns between taxonomic entries of the gut tissue and gut digesta, determined through Co-occurrence Network inference (CoNet v1.1.1), and analyzed through Cytoscape (v3.6.0). Taxonomic entries with a cumulative row sum of 200 or above with 2/3 of samples showing non-zero value entries were used through an ensemble approach that incorporated the Pearson, Spearman, Bray–Curtis, Kullback–Leibler, and mutual information metrics. The top and bottom 200 edges were selected and merged by the union method. (**A**) The network analysis shows the edges represented by the q-value (merged with the Brown method at *p* < 0.05 for each metric) and are shown as green (co-presence) and red (co-exclusion), with the nodes representing taxa were scaled according to relative abundance and colored according to the phyla (class for Proteobacteria) assignments. The final network was arranged using the yFiles (v1.0) Cytoscape (v3.6.0) add-on radial layout, and taxonomic entries shown at the highest resolution are denoted with the sample type (circle for gut tissue, “-gut”; diamond for gut digesta; “-dig”). The network has been generated using scalable graphics, and therefore nodes of interest can be viewed at a higher resolution digitally by increasing the magnification. (**B**) Scatter plot analysis was performed using topological metrics determined by NetworkAnalyzer (v2.7), to demonstrate patterns of key (keystone) species between taxonomic entries of the gut tissue and gut digesta based on closeness and betweenness centrality, as well as the degree (number of co-presence and co-exclusion edges). Linear regression between closeness and betweenness centrality was shown as logarithmic (R^2^ value = 0.7145), and the top 10 entries ranked by closeness centrality are depicted. Note, the taxa Rhodophyta and Rhodobacteraceae had the same closeness and betweenness centrality measurements, and their corresponding plot is indistinguishable. Linear regression determined through Microsoft Excel Software (Seattle, WA).

The resultant network generated through CoNet (v1.1.1) in Cytoscape (v3.6.0) yielded 71 nodes and 294 edges elucidating possible interactions occurring between taxa representing the distinct microbial communities of the sea urchin gut environment (Figure 10A). Analysis of network properties using NetworkAnalyzer (v2.7) showed an average network centralization of 0.128, the characteristic path length of 2.856, average number of neighbors at 8.282, with a network density of 0.118 and network heterogeneity of 0.452. Scatter plot analysis demonstrated the trends of closeness centrality plotted against betweenness centrality, along with the nodes scaled to the degree (Figure 10B). The top 10 candidate key taxa based on the topological qualities of taxonomic nodes described in Berry and Widder et al. [30] were ranked by their closeness centrality, and showed *Propionigenium*, *Moritella*, SB-1 (Bacteroidetes), Desulfobacteraceae and *Desulfovibrio* in the gut digesta, and Rhodobacteraceae, Rhodophyta, Vibrionaceae, *Arcobacter* and *Bacilli* in the gut tissue (Table 3). Of these taxa, the gut digesta showed *Propionigenium* to have the highest degree of associations with the gut tissue taxa (17 total), with the majority of these associations shown as co-presence (14 total). The gut tissue showed Rhodobacteraceae to have the highest degree (9 total), and most of these associations were co-exclusion (8 total). The gut tissue also showed the highly abundant *Arcobacter* to be a likely key taxon, revealing a degree of 9, with the majority of these associations as co-presence (7 total).

## 4. Discussion

Overall, the *S. purpuratus* gut ecosystem exhibited high abundances of *Arcobacter* and *Sulfurimonas* in the gut tissues. These taxa belong to phylum Epsilonproteobacteria, and specifically within the order Campylobacterales, members of which are known to be chemolithoautotrophic [88]. Both *Arcobacter* and *Sulfurimonas* have been implicated as marine sulfur-oxidizing bacteria, with members of *Arcobacter* forming oxidized sulfur filaments in response to geothermally produced sulfide in hydrothermal vents [89,90], and *Sulfuromonas* has been observed to oxidize sulfur in anoxic deep-sea hydrothermal sediments [91,92]. Additionally, Epsilonproteobacteria have been reported in the gut systems of marine organisms, with *Arcobacter* previously observed in the deep-sea vent-dwelling shrimp *Rimicaris exoculata* [93] and the marine Chilean oyster *Tiostrea chilensis* [94], and *Sulfurimonas* in the stomach of the hydrothermal vent crab *Xenograpsus testudinatus* [95] and as symbionts in marine gastropod mollusks *Alviniconcha* [96,97]. These bacteria have been implicated as potential symbionts, assisting in the oxidation of sulfur in the highly sulfidic environments [98]. Previous studies using Illumina MiSeq HTS of the V4 region of the collective 16S rRNA genes conducted on the gut environment of the sea urchin *L. variegatus* from the Gulf of Mexico similarly revealed Campylobacterales to be near-exclusively abundant in the gut tissue [15,16], of which further analysis of the highly-represented Campylobacterales sequence showed a ~90-91% similarity match to *Arcobacter*/*Sulfuricurvum* through NCBI BLAST [15].

In contrast to the gut tissue, the microbial composition of gut digesta was dominated by *Psychromonas* (phylum Gammaproteobacteria). This halophilic genus is known to occur in cold marine environments (~4 °C) [99], and members are capable of hydrolyzing starch and other insoluble sugars [100,101,102,103], as well as producing ω-3 polyunsaturated fatty acids [99]. It has recently been shown that the total ω-3 fatty acids of the gut contents (23.26 ± 6.88; mean ± SD) of experimentally fed *S. purpuratus* are higher than the total ω-3 fatty acids of their algal diets (13.41 ± 6.32); the dominant driver of this pattern was from the ω-3 eicosapentaenoic acid (EPA; 20:5ω-3) [20], but the mechanism for this enrichment was unknown. The psychrophilic capabilities of *Psychromonas* are consistent with the temperature of the Oregon Pacific Coast seawater temperature from which *S. purpuratus* was sampled, which was recorded at 13.1 °C, within the range of optimum growth *Psychromonas* spp. [100]. Also observed in the gut digesta were *Propionigenium* of phylum Fusobacteria, members of which are anaerobic, are capable of fermenting succinate to propionate [104] and have been implicated in carbohydrate metabolisms that include cellulose, producing short-chain fatty acids [105]. *Propionigenium* is involved in a myriad of host health benefits, including its association in the modulation of the lifespan of the Turquoise Killifish (*Nothobranchius furzeri*) [105]. Order Flavobacteriales of phylum Bacteroidetes were also observed, which have been identified in the intestines of shrimp *Litopenaeus stylirostris* raised in aquaculture and clear waters [106]. Importantly, members of Flavobacterales have been investigated with consideration of their metabolic capability to break-down alginate from brown seaweeds common in both temperate and polar coastal environments [107]. Lastly, the genus *Desulfotalea* of Deltaproteobacteria was observed at approximately equal relative abundances in the gut digesta and gut tissue (~5% in each). Certain members of *Desulfotalea* are adapted to extremely cold environments, and these bacteria are capable of sulfur reduction [108,109]. 

Shannon and Simpson’s diversity measured for all samples showed the microbial communities of the gut environment to have low diversity, whereas the pharynx and environmental samples had a high diversity value. Such high microbial diversity in the marine environment indicates a rich microbial community and is expected due to the fluctuations of abiotic conditions and nutrients, spatial dispersion of microbes through the marine environment, and dynamic host-species populations that shape the microbiota to the environment [21]. The reduction of species richness from the environment to the gut tissue and digesta indicated a more preferential microbial community comprised of specific taxa in the gut system. 

Compartmentalization of microbial community profiles in the gut environment of *S. purpuratus* was demonstrated through Bray-Curtis based 2D MDS plot and dendrogram cluster analysis and was supported through heatmap and ANOSIM/Adonis analysis. Grouping based on sample type was found to be significant through ANOSIM/Adonis statistics, indicating the consistency of microbial taxa across biological replicates representing each group (pharynx, gut tissue, gut digesta, water, and algae). 2D MDS plot analysis supported this low intra-sample variation within groups, and dendrogram analysis showed the gut tissues to cluster nearer the gut digesta, and the pharynx nearer the water samples. 2D MDS plot and dendrogram analysis of the top 100 OTUs strengthened the trends observed when using the rarefied OTU table. Additionally, an intra-sample variation of the rare taxa was not markedly transformed when determined through 2D MDS plot and dendrogram analysis. However, analysis using rare taxa slightly altered the cluster patterns of groups, showing the gut tissue to cluster nearer the water and pharynx, suggesting shared rare microbial taxa between these groups. Interestingly, although the weighted and unweighted Unifrac approaches did support the significant within-group similarities observed through Bray–Curtis based 2D MDS and dendrogram analysis, the between-group cluster patterns were slightly transformed. For weighted Unifrac, which measures the distance between samples using the relative abundances and phylogenetic relationships of the presented microorganisms [70], the gut tissue and digesta samples clustered closer together. However, through the unweighted method, which considers the presence/absence of taxa and their phylogeny [70], the gut tissue clustered with the water and pharynx samples. Such differences suggest an underlying phylogenetic relatedness between those bacterial microbiota colonizing the gut system based on their abundance. For all ordination analyses performed in this study, the microbial communities of the algae remained the least similar to all samples of the study. 

Heatmap analyses supported the 2D MDS plot and dendrogram analysis, showing the contribution of Epsilonproteobacteria (*Arcobacter* and *Sulfurimonas*) to the unique microbial profile of the gut tissue, and *Psychromonas* in the gut digesta. LEfSe analysis demonstrated the significant effect size of certain key bacterial microbiota contributing to microbial community dissimilarity of the gut tissue and digesta, showing *Arcobacter*, *Sulfurimonas*, and *Tissierella_Soehngenia* in the gut tissue, and *Psychromonas*, Flavobacteriales, and *Vibrio* in the gut digesta. Although *Propionigenium* was found to be more heightened in the gut digesta (14.9% ± 9.6%) than the gut tissue (5.7% ± 4.8%), this taxon did not contribute to a high LDA score. This is likely due to the overlapping standard deviation of the relative abundance values of this taxon (Figure 3). Thus, these beta diversity analyses demonstrate the contribution of all taxonomic groups (heightened and rare taxa) in shaping both intra-sample and group-wise microbial profile similarity in the sea urchin *S. purpuratus*, as well as the specific taxa contributing to the unique cluster patterns and effect size of the compartmentalized gut tissue and digesta bacteria. 

Public repositories of taxonomic information, such as the GreenGenes (v13.8) database used in this study, often possess repetitive entries, resulting in OTU tables with many redundant taxonomic assignments [25]. By using PhyloToAST (v1.4.0), such bias in the in the representation of 16S rRNA gene and associated taxonomic information can be improved, by binning highly similar taxonomic OTU sequences and assigning taxonomy to a higher resolution (genus/species when possible) through BLAST. Other strategies to improving the diversity measures of OTU tables generated from a targeted HTS approach have been proposed, such as the LULU algorithm that utilizes a post-clustering strategy to curate an OTU table based on co-occurrence patterns to help eliminate spuriously generated OTUs from the final dataset [110]. In this study, the initial OTU table generated following quality checks resulted in 44,664 unique assignments. However, condensing of redundant taxonomic IDs through PhyloToAST (v1.4.0) reduced the number of unique observations to 776, without any loss of read-count data when compared to the filtered OTU table. Additionally, subsampling of the condensed OTU table to the minimum read count (49,641) increased the beta diversity significance between biological replicate groups. Thus, the combined use of PhyloToAST (v1.4.0) and subsampling to the minimum value, enhanced the resolution of taxa, as well as increased the robustness of the beta analyses measures. 

The popularity of targeted high throughput sequencing of microbial 16S rRNA genes has spurred the development of multiple bioinformatics tools, techniques, and approaches, designed to ensure the quality and reliability of the generated results. Traditionally, following the quality assessment and filtering of raw sequence read data, OTUs are chosen based on clustering highly similar sequences at a designated threshold, such as the 97% pairwise similarity described in our analysis, and commonly used as a proxy for species based on the 16S rRNA gene [111,112]. This OTU picking approach will often generate an initially high amount of unique observations, spuriously inflate the alpha diversity, and skew beta diversity measures, due to PCR and/or sequencing errors generating a high number of very rare OTUs [49]. Therefore, an additional filtration step is often necessary, such as the removal of OTUs occurring at <0.0005% average abundance across all samples as applied in our study and others [50,51], and described for Illumina MiSeq generated sequence read data [49,52]. Alternative approaches to this strategy include a current practice of selecting ASVs as the operational units, which are generated by coupling a denoising step on the raw data that employs a parametric error model to define the frequency of observed sequence variants as it relates to real biological sequence data [43,44]. One such ASV selection tool, DADA2 (v1.10) as implemented in the recently developed QIIME2 package (v2018.11), utilizes a Poisson model to determine these repeated sequence variants [43,44], and was used as an alternative approach to our OTU method. For our OTU-based approach, the initial unfiltered OTU table generated a high number of unique observances, which were further filtered to eliminate potential spurious sequence reads, and then condensed through a BLAST approach with PhyloToAST (v1.4.0) and rarefied to the minimum sequence value. As previously stated, this produced 776 unique OTUs with 49,641 reads across all samples. After using DADA2 (v1.10) through QIIME2 (v2018.11), a total of 1134 unique ASVs with an average read count of ~31,191 were generated across all samples, producing a quantitatively comparable sequence read count to the rarefied OTU table used in our downstream analyses. Additionally, following taxonomic assignment of the representative sequences generated for both the OTUs and ASVs using GreenGenes (v13.8), a similar relative abundance distribution was determined at comparable levels of phylogenetic resolution. Some exceptions included the additional resolution of one OTU assigned as phylum Gammaproteobacteria in the pharynx tissue, which was further resolved to order Legionellales through the ASV method. However, the similarities of relative abundance are consistent with the most current literature comparing the two strategies [52]. Lastly, it should be noted that although DADA2 denoising may be advantageous in determining real biological sequences associated with rare taxa, this may come at the cost of determining “false positives” as described elsewhere [52]. 

Predicted functional profiles generated for the gut tissue and digesta at KEGG-Level-3 showed enrichment of metabolic qualities related to energy metabolisms, including oxidative phosphorylation, carbon fixation, methane, and nitrogen metabolisms in the gut tissue. The gut tissue also included heightened motility-related categories, suggesting a potential role of these functional attributes in the colonization of the gut tissue lumen [113], potentially through abiotic factors driving microbial colonization into the more oxygenated sea urchin gut tissue surface [114]. In the gut digesta, KEGG-Level-3 categories related to protein metabolisms (e.g., peptidases and amino acid enzymes, arginine and proline metabolisms) and carbohydrate metabolisms (e.g., starch and sucrose metabolism, pentose phosphate pathway, glycolysis/gluconeogenesis, and ubiquinone and other terpenoid-quinone biosynthesis) suggest a potential role of these bacteria in the digestive physiology of the purple sea urchin. These results are consistent with previous culture-dependent studies of sea urchin gut bacteria in digestion [9] and reflect potential anaerobic metabolisms likely to be performed in this mucous-sequestered niche [115]. LEfSe analysis at the KO Id level indicated the categories that contributed most to the differences between the distinct microbial communities of the gut system and supported the observed KEGG-Level-2 and 3 categories. The gut tissue presented seven categories related to membrane transporters, such as multiple sugar transport system permease proteins, cobalt/nickel transport system ATP-binding proteins and ferrous iron transport protein A, including ABC transport proteins such as the sulfonate/nitrate/taurine transport system permease protein category. Categories related to nitrogen metabolism, and specifically nitrate reduction, were also observed as heightened. Such nitrate reductive activity occurring in the sea urchin gut has been previously suggested to occur by the gut microbiota of sea urchins [14,116,117]. The gut digesta represented categories related to amino acid metabolism related to threonine and histidine biosynthesis, vitamin metabolisms such as Menaquinone (vitamin K2) production, and categories related to carbohydrate metabolism in the pentose phosphate pathway. These categories support the trends observed in the scatter plot analysis and offer insight into the functional qualities that accompany the life strategies of the gut microbes that may drive their distribution in the compartmentalized gut system. Lastly, although the calculated NSTI values (avg. = 0.139) for the data in this study indicated adequate confidence in the functional predictions of non-human-associated microbial communities based on taxonomic inference alone [75], a shotgun metagenomics approach targeting the metacommunity DNA of a microbiome sample would offer more reliable insight into those genes likely involved in host health and digestion. 

We used CoNet (v1.1.1) analysis to identify theoretical modeling of relationships occurring between taxa in the distinctly compartmentalized gut tissue and gut digesta microbial communities [27,28,29]. Although taxa that were noticeably abundant in this study were represented (such as *Propionigenium* in the gut digesta and *Arcobacter* in the gut tissue), there were many low-abundance taxa representing likely key taxa. For example, *Moritella* (Gammaproteobacteria, <1% in the gut digesta) were observed to have a high closeness centrality relative to betweenness centrality and a high degree of edges (16 total) with 10 positive associations to taxa in the gut digesta. Interestingly, two taxa identified as Desulfobacteraceae and *Desulfovibrio* to the highest resolution were included in the top 10 likely key taxa in the gut digesta, were sulfur-reducing bacteria (<1% in the gut digesta) and represented a total degree of 14 and 13 respectively, with the majority of these indicated as co-exclusion relationships. These taxa belong to the phylum Deltaproteobacteria and represent species known to utilize sulfate as electron acceptors [118]. In the gut tissue, Rhodobacteraceae (Alphaproteobacteria) and order Rhodophyta (classified in phylum Cyanobacteria according to the GreenGenes v13.8 database) revealed the same pattern of degree (nine each), closeness and betweenness centrality measures. Additionally, *Arcobacter*, which was highly abundant in the gut tissue (~ 20%), was identified as a key taxon with a degree of nine (with seven positive associations). This genus is comprised of species capable of utilizing elemental sulfur, hydrogen sulfide, and thiosulfate as terminal electron acceptors [119], alluding to a biogeochemical basis for the observed ecological relationships as suggested previously [120]. However, the relationship between microbial taxa of the gut tissue and gut digesta, including the role or key status of particular microbial taxa will require further verification. 

In summary, the results of this study provide insight into the gut bacterial microbiota of *S. purpuratus* grown *in situ*, specifically elaborating (1) the taxonomic distribution, (2) the predicted functional categories assigned to the gut-associated bacterial communities, and (3) key taxa likely involved in maintaining the distribution patterns of gut microbiota through co-occurrence relationships in this evolutionarily and ecologically significant deuterostome. The gut environment demonstrated an allocation of microbial communities in the gut tissue, with a heightened abundance of Epsilonproteobacteria (namely *Arcobacter* and *Sulfurimonas*), and in the gut digesta, a higher abundance of Gammaproteobacteria (such as the psychrophilic genus *Psychromonas*). This trend of microbial compartmentalization has been previously observed in the laboratory [15,24] and naturally occurring [16] *L. variegatus* sea urchin bacteriome from the Gulf of Mexico. In those studies, certain microbial taxa were found to be consistent between the gut systems of both laboratory-raised and naturally occurring organisms, with the naturally occurring organisms showing slightly higher species diversity and richness in the gut system. More specifically, a near-exclusive abundance of *Arcobacter* (Epsilonproteobacteria) was observed in the gut tissue, whereas *Vibrio* (Gammaproteobacteria), a genus common to halophilic temperate marine environments, was most dominant in the gut digesta. Other bacterial microbiota found consistent in the gut digesta of *L. variegatus* included *Propionigenium* and taxa assigned as family Rhodobacteraceae. For both the laboratory-raised and naturally occurring sea urchins, beta diversity analysis of the pharynx showed cluster patterns that diverged from the gut tissue and digesta and more closely resembled that of their environment. Interestingly, many of the same trends of microbial integration in *L. variegatus* were also observed in the naturally occurring sea urchin *S. purpuratus* gut system of this study. This included an abundance of Epsilonproteobacteria such as *Arcobacter* and *Sulfurimonas* in the gut tissue, Gammaproteobacteria such as *Psychromonas* and *Vibrio*, as well as other common species such as *Propionigenium* in the gut digesta, and a pharynx microbial community resembling the environment. Such selective enrichment and compartmentalization of bacteria in both *S. purpuratus* and *L. variegatus* despite geographical separation support an essential role of specific bacterial taxa to their hosts’ health and digestion, and could be further supported by future studies establishing the gut microbiome of laboratory-raised counterparts and/or sea urchins collected at different time-points and locations. Lastly, whether this trend of bacterial enrichment into the sea urchin gut system is the result of (1) naturally occurring microbes in adjacent seawater finding a suitable habitat in the urchin gut environment to flourish, or (2) a host-mediated selective integration of key bacterial microbiota, remains to be verified. However, the similarities identified in taxa across geographical scales suggests that this could be an interesting avenue for future study.

## Figures and Tables

**Table 1:** Sequence reads, operational taxonomic unit (OTU) count, and alpha diversity of each sample of the study. The table shows the sequence read count (1) before and (2) after quality checking and filtering based on low quality reads using FASTX Toolkit, the (3) unique unfiltered (Unfilt.) OTU observances following chimera removal, (4) the filtered (Filt.) OTUs after removal of rare OTUs (<0.0005% abundant in all samples), (5) the resultant condensed (Cond.) OTU count following the merging of redundant taxonomic information through Phylogenetic Tools for Analysis of Species-level Taxa (PhyloToAST) (v1.4.0), (6) the OTU count following subsampling to the median (Med.) value (77,806), and (7) the minimum (Min.) value (49,641). Also shown are the (8) Shannon and (9) Simpson diversity indices corresponding to each sample determined using the condensed OTU table data.

Sample	Raw Reads	Trimmed Reads	Unfilt. OTUs	Filt. OTUs	Cond. OTUs	Cond. OTUs Med.	Cond. OTUs Min.	Shannon Diversity	Simpson Diversity
Algae 1	154,561	121,543	4985	1345	268	259	243	5.3590	0.9376
Algae 2	137,323	103,116	2973	855	226	220	204	4.7960	0.9340
Algae 3	160,926	115,779	4745	1383	329	325	302	4.9626	0.9156
Gut Digesta 1	76,329	60,871	2133	580	155	155	151	3.1936	0.8260
Gut Digesta 2	74,249	61,267	2144	599	119	119	113	2.4760	0.5991
Gut Digesta 3	123,640	99,546	3056	611	126	121	106	2.9289	0.7891
Gut Tissue 1	128,539	90,412	4384	1094	328	322	301	4.1236	0.8440
Gut Tissue 2	68,644	51,895	2311	898	273	273	273	3.8787	0.8436
Gut Tissue 3	123,735	89,547	4765	1418	403	397	368	4.8038	0.9250
Pharynx Tissue 1	107,276	72,954	5663	1558	430	430	418	6.0719	0.9642
Pharynx Tissue 2	86,515	58,281	4417	1488	431	431	430	5.9389	0.9632
Pharynx Tissue 3	92,987	63,405	4918	1489	402	402	397	6.2246	0.9697
Water 1	123,154	81,885	4725	1679	504	504	487	5.2838	0.8797
Water 2	137,044	93,713	6127	1087	403	400	386	5.7289	0.9546
Water 3	119,824	85,613	5608	1406	400	400	377	4.2286	0.8211
Summary	total = 1,714,746	total = 1,249,827	total = 44,664	total = 4290	total = 776	total = 776	total = 776	avg. = 4.6666	avg. = 0.8778

**Table 2:** Grouping statistics performed on each OTU table generated in the study. Results show both ANOSIM and Adonis measurements of the initial OTU table (unfiltered; Unfilt.), followed by filtering of those taxa represented at <0.0005% in the study (filtered; Filt.), condensing (Cond.) using PhyloToAST (v1.4.0) and rarefying to the median (Med.) and minimum (Min.) of the condensed OTU table file. Analysis was performed on grouped biological sample replicates (pharynx, *n* = 3; gut tissue, *n* = 3; gut digesta, *n* = 3; water, *n* = 3; and algae, *n* = 3).

Diversity Measure	Unfilt. OTUs	Filt. OTUs	Cond. OTUs	Cond. OTUs Med.	Cond. OTUs Min
ANOSIM	R = 0.93185	R = 0.93185	R = 0.94074	R = 0.94074	R = 0.94222
Adonis	R2 = 0.69518	R2 = 0.71145	R2 = 0.74894	R2 = 0.74951	R2 = 0.75688

**Table 3:** Candidate key taxa resulting from CoNet (v1.1.1) analysis between the gut tissue and gut digesta microbial communities. The group assignment is shown (gut tissue, *n* = 3; gut digesta, *n* = 3), along with the phylum and taxon at the highest resolution. The average abundance (Av. Ab.) was determined for each taxon based on the respective group (gut tissue or digesta as indicated). The entries were ranked according to closeness centrality (Clos. Cent.), highest to lowest (a feature of keystoneness), and then by degree (edges; Deg.), highest to lowest. Also shown is the betweenness centrality (Bet. Cent.), and the total, positive (co-presence; Pos. Deg.) and negative (co-exclusion; Neg. Deg.) degree values.

Sample Type	Phylum	Taxon	Av. Ab.	Clos. Cent.	Bet. Cent.	Deg.	Pos. Deg.	Neg. Deg.
Gut Digesta	Fusobacteria	*Propionigenium*	14.89%	0.39	0.13	17	14	4
Gut Digesta	Proteobacteria (Gammaproteobacteria)	*Moritella*	0.15%	0.44	0.17	16	10	6
Gut Digesta	Bacteroidetes	SB-1	0.38%	0.39	0.06	14	3	11
Gut Digesta	Proteobacteria (Deltaproteobacteria)	Desulfobacteraceae	0.24%	0.38	0.04	14	2	12
Gut Digesta	Proteobacteria (Deltaproteobacteria)	*Desulfovibrio*	0.24%	0.41	0.11	13	6	7
Gut Tissue	Proteobacteria (Alphaproteobacteria)	Rhodobacteraceae	0.34%	0.41	0.05	9	1	8
Gut Tissue	Cyanobacteria	Rhodophyta	0.22%	0.41	0.05	9	1	8
Gut Tissue	Proteobacteria (Gammaproteobacteria)	Vibrionaceae	0.36%	0.41	0.09	9	5	5
Gut Tissue	Proteobacteria (Epsilonproteobacteria)	*Arcobacter*	20.59%	0.38	0.04	9	7	2
Gut Tissue	Firmicutes	*Bacilli*	0.16%	0.38	0.06	8	6	2

## Supplementary Materials

The following are available online at http://www.mdpi.com/2076-2607/7/2/35/s1: Figure S1: 2D multidimensional scale (MDS) plot and dendrogram analysis performed using the Bray–Curtis metrics calculated for the top 100 and rare taxa across all samples of the study; Figure S2: 3D principle coordinates analysis (PCoA) plot and dendrogram analysis performed using the weighted Unifrac metrics calculated for the rarefied OTU table data across all samples of the study; Figure S3: 3D principle coordinates analysis (PCoA) plot and dendrogram analysis performed using the unweighted Unifrac metrics calculated for the rarefied OTU table data across all samples of the study; Table S1: The OTU table data to most resolvable level of the merged biological replicate samples of this study (pharynx, *n* = 3; gut tissue, *n* = 3; gut digesta, *n* = 3; water, *n* = 3; and algae, *n* = 3) with taxonomy assigned using the GreenGenes (v13.8) database; Table S2: The unfiltered OTU table data of all samples of this study with taxonomy assigned using the GreenGenes (v13.8) database; Table S3: The QIIME2 (v2018.11) ASV table data generated through DADA2 (v1.10) for all samples in this study with taxonomy assigned using the GreenGenes (v13.8) database; Table S4: Top 100 representative sequences aligned to multiple databases; Table S5: Results of the predicted functional profiles determined through PICRUSt (v1.1.2) and analyzed through STAMP (v2.1.3) of the Kyoto Encyclopedia of Genes and Genomes (KEGG) Level-2 and KEGG-Level-3 categories (gut tissue, *n* = 3; gut digesta, *n* = 3); Table S6: Description of each functional category determined through LEfSe analysis (LDA threshold at ± 2.4) of the KEGG Ortholog (KO) Ids determined through PICRUSt (v1.1.2) analysis of the gut tissue and gut digesta microbial communities.
