# Supplementary material for: The Purple Sea Urchin Strongylocentrotus purpuratus Demonstrates a Compartmentalization of Gut Bacterial Microbiota, Predictive Functional Attributes, and Taxonomic Co-Occurrence
Source: Microorganisms. 2019 Jan 26;7(2):35. doi: 10.3390/microorganisms7020035 (PMC6406795; doi:10.3390/microorganisms7020035)
Supplement: Supplementary file 1 [file microorganisms-07-00035-s001.zip › Sup_Data_Final/Fig_S1_top-rare_beta_div_r1_edit-final.pdf]

Title: The Purple Sea Urchin *Strongylocentrotus Purpuratus* Demonstrates a Compartmentalization of Gut Bacterial Microbiota, Predictive Functional Attributes, and Taxonomic Co-Occurrence

Authors:
Joseph A. Hakim[1,*], Julie B. Schram[2], Aaron W. E. Galloway[2], Casey D. Morrow[3], Michael R. Crowley[4], Stephen A. Watts[1], and Asim K. Bej[1,*]

Corresponding Author *
Affiliations:
[1]Department of Biology, University of Alabama at Birmingham, 1300 University Blvd., Birmingham, AL 35294, USA; joe21@uab.edu (J.A.H); sawatts@uab.edu (S.A.W.); abej@uab.edu (A.K.B)
[2]Oregon Institute of Marine Biology, University of Oregon, 63466 Boat Basin Rd, Charleston, OR 97420, USA; jschram@uoregon.edu (J.B.S); agallow3@uoregon.edu (A.W.E.G.)
[3]Department of Cell, Developmental and Integrative Biology, University of Alabama at Birmingham, 1918 University Blvd., Birmingham, AL 35294, USA; caseym@uab.edu (C.D.M)
[4]Department of Genetics, Heflin Center Genomics Core, School of Medicine, University of Alabama at Birmingham, 705 South 20th Street, AL 35294 USA; mcrowley@uab.edu (M.R.C)

*****Correspondence: Joseph A. Hakim (joe21@uab.edu) (Primary corresponding author); Asim K. Bej (abej@uab.edu) (Submitting corresponding author); Tel.: +1 (205) 934-9857.

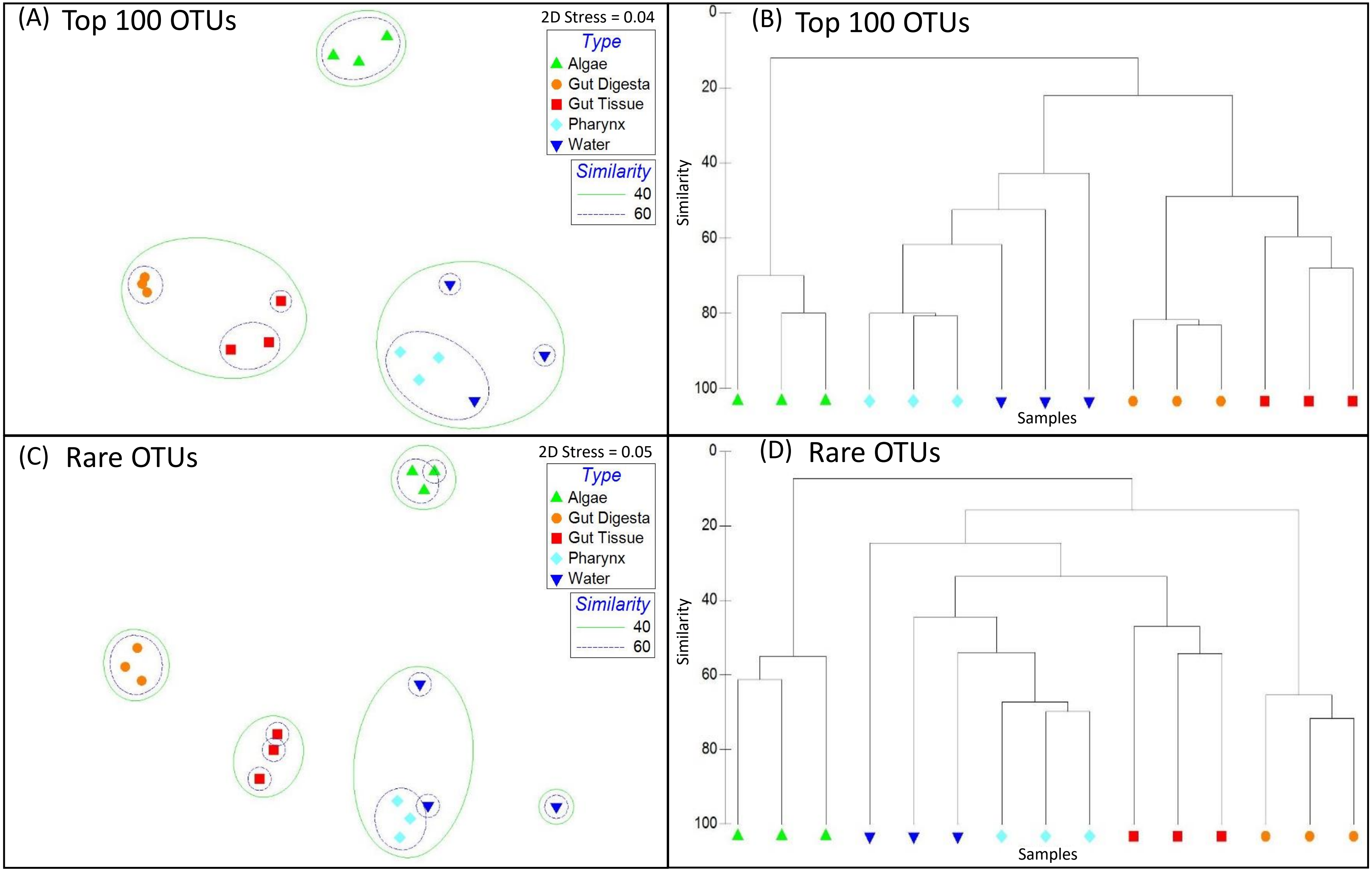

Supplementary Figure 1: 2D multidimensional scale (MDS) plot and dendrogram analysis performed using the Bray-Curtis metrics calculated for the top 100 and rare taxa across all samples of the study. (A) 2D MDS and (B) dendrogram analysis of the top 100 OTUs, and (C) 2D MDS and (D) dendrogram analysis of the rare OTUs (non-top 100) determined across all samples of the study. The rarefied OTU table was pre-treated prior to calculating the Bray-Curtis similarity, by standardizing each OTU by the total sequence count per sample (to reduce the large disparities between samples), and then by log transforming the data transform to lessen the dominant contribution of highly abundant OTUs. The overlay of similarity observed in the 2D MDS plots is shown as 40% and 60% Bray-Curtis similarity. Figure legends are shown in the 2D MDS plots, including the 2D Stress values. Data was generated and plotted through PRIMER-6 software (Primer-E Ltd, Plymouth Marine Laboratory, Plymouth UK, v6.1.2)
