# Supplementary material for: The Purple Sea Urchin Strongylocentrotus purpuratus Demonstrates a Compartmentalization of Gut Bacterial Microbiota, Predictive Functional Attributes, and Taxonomic Co-Occurrence
Source: Microorganisms. 2019 Jan 26;7(2):35. doi: 10.3390/microorganisms7020035 (PMC6406795; doi:10.3390/microorganisms7020035)
Supplement: Supplementary file 1 [file microorganisms-07-00035-s001.zip › Sup_Data_Final/Table_S1_rarefied_min_OTU_table-final.docx]

**Title:** The Purple Sea Urchin *Strongylocentrotus Purpuratus* Demonstrates a Compartmentalization of Gut Bacterial Microbiota, Predictive Functional Attributes, and Taxonomic Co-Occurrence

**Authors:**

Joseph A. Hakim^1,*^, Julie B. Schram^2^, Aaron W. E. Galloway^2^, Casey D. Morrow^3^, Michael R. Crowley^4^, Stephen A. Watts^1^, and Asim K. Bej^1,*^

**Corresponding Author ^*^**

**Affiliations:**

^1^ Department of Biology, University of Alabama at Birmingham, 1300 University Blvd., Birmingham, AL 35294, USA; joe21@uab.edu (J.A.H); sawatts@uab.edu (S.A.W.); abej@uab.edu (A.K.B)

^2^ Oregon Institute of Marine Biology, University of Oregon, 63466 Boat Basin Rd, Charleston, OR 97420, USA; jschram@uoregon.edu (J.B.S); agallow3@uoregon.edu (A.W.E.G.)

^3^ Department of Cell, Developmental and Integrative Biology, University of Alabama at Birmingham, 1918 University Blvd., Birmingham, AL 35294, USA; caseym@uab.edu (C.D.M)

^4^ Department of Genetics, Heflin Center Genomics Core, School of Medicine, University of Alabama at Birmingham, 705 South 20th Street, AL 35294 USA; mcrowley@uab.edu (M.R.C)

***** Correspondence: Joseph A. Hakim (joe21@uab.edu) (Primary corresponding author); Asim K. Bej (abej@uab.edu) (Submitting corresponding author); Tel.: +1 (205) 934-9857.

**Supplementary Table 1:** The OTU table data to most resolvable level of the merged (*n* = 3) biological replicate samples of this study (pharynx, *n* = 3; gut tissue, *n* = 3; gut digesta, *n* = 3; water, *n* = 3; and algae, *n* = 3) with taxonomy assigned using the GreengGenes (v13.8) database. Also shown is the OTU ID assigned to the representative sequence corresponding to the taxonomic identity.

| **Taxonomy** | **Water** | **Pharynx** | **Gut Tissue** | **Gut Digesta** | **Algae** | **OTU ID** |
| --- | --- | --- | --- | --- | --- | --- |
| k__Archaea | 1 | 0 | 10 | 2 | 0 | denovo13596 |
| k__Archaea; p__Crenarchaeota; c__Thaumarchaeota; o__Cenarchaeales; f__Cenarchaeaceae; g__Nitrosopumilus; s__ | 3 | 0 | 1 | 5 | 1 | denovo43920 |
| k__Archaea; p__Euryarchaeota; c__Methanobacteria; o__Methanobacteriales; f__Methanobacteriaceae; g__Methanobrevibacter; s__ | 2 | 0 | 2 | 13 | 3 | denovo7913 |
| k__Archaea; p__Euryarchaeota; c__Methanomicrobia; o__Methanomicrobiales; f__Methanocorpusculaceae; g__Methanocorpusculum; s__ | 0 | 166 | 772 | 1 | 0 | denovo19931 |
| k__Archaea; p__Euryarchaeota; c__Thermoplasmata; o__E2; f__Marine group II; g__; s__ | 0 | 0 | 1 | 0 | 34 | denovo15575 |
| k__Bacteria | 1093 | 584 | 3034 | 4440 | 4748 | denovo20030 |
| k__Bacteria; p__[Thermi]; c__Deinococci | 3 | 0 | 0 | 0 | 0 | denovo6928 |
| k__Bacteria; p__[Thermi]; c__Deinococci; o__Deinococcales; f__Deinococcaceae; g__Deinococcus; s__ | 0 | 0 | 12 | 4 | 37 | denovo9154 |
| k__Bacteria; p__[Thermi]; c__Deinococci; o__Deinococcales; f__Deinococcaceae; g__Deinococcus; s__geothermalis | 0 | 0 | 0 | 0 | 7 | denovo19456 |
| k__Bacteria; p__[Thermi]; c__Deinococci; o__Deinococcales; f__Trueperaceae | 93 | 0 | 0 | 0 | 0 | denovo27760 |
| k__Bacteria; p__[Thermi]; c__Deinococci; o__Deinococcales; f__Trueperaceae; g__; s__ | 2025 | 0 | 0 | 0 | 0 | denovo4830 |
| k__Bacteria; p__[Thermi]; c__Deinococci; o__Thermales; f__Thermaceae; g__Meiothermus; s__ | 0 | 0 | 0 | 14 | 60 | denovo26358 |
| k__Bacteria; p__[Thermi]; c__Deinococci; o__Thermales; f__Thermaceae; g__Thermus; s__ | 0 | 0 | 0 | 0 | 42 | denovo7372 |
| k__Bacteria; p__Acidobacteria; c__[Chloracidobacteria]; o__RB41; f__; g__; s__ | 1 | 0 | 0 | 9 | 5 | denovo28978 |
| k__Bacteria; p__Acidobacteria; c__[Chloracidobacteria]; o__RB41; f__Ellin6075; g__; s__ | 0 | 0 | 0 | 0 | 187 | denovo25186 |
| k__Bacteria; p__Actinobacteria | 5 | 0 | 0 | 0 | 0 | denovo1906 |
| k__Bacteria; p__Actinobacteria; c__Acidimicrobiia; o__Acidimicrobiales | 718 | 0 | 0 | 0 | 0 | denovo40641 |
| k__Bacteria; p__Actinobacteria; c__Acidimicrobiia; o__Acidimicrobiales; f__; g__; s__ | 179 | 0 | 0 | 0 | 3 | denovo19520 |
| k__Bacteria; p__Actinobacteria; c__Acidimicrobiia; o__Acidimicrobiales; f__C111; g__; s__ | 116 | 6 | 62 | 0 | 11 | denovo21762 |
| k__Bacteria; p__Actinobacteria; c__Acidimicrobiia; o__Acidimicrobiales; f__JdFBGBact; g__; s__ | 2310 | 3 | 1 | 2 | 19 | denovo3336 |
| k__Bacteria; p__Actinobacteria; c__Acidimicrobiia; o__Acidimicrobiales; f__ntu14; g__; s__ | 6 | 0 | 0 | 0 | 0 | denovo15863 |
| k__Bacteria; p__Actinobacteria; c__Acidimicrobiia; o__Acidimicrobiales; f__OCS155; g__; s__ | 0 | 0 | 3 | 12 | 41 | denovo31147 |
| k__Bacteria; p__Actinobacteria; c__Acidimicrobiia; o__Acidimicrobiales; f__SC3-41; g__; s__ | 1139 | 0 | 0 | 1 | 9 | denovo31527 |
| k__Bacteria; p__Actinobacteria; c__Acidimicrobiia; o__Acidimicrobiales; f__ZA3409c; g__; s__ | 107 | 0 | 0 | 0 | 3 | denovo42326 |
| k__Bacteria; p__Actinobacteria; c__Actinobacteria; o__Actinomycetales | 0 | 0 | 0 | 0 | 54 | denovo11698 |
| k__Bacteria; p__Actinobacteria; c__Actinobacteria; o__Actinomycetales; f__Actinomycetaceae; g__Actinomyces; s__ | 0 | 0 | 48 | 528 | 741 | denovo37709 |
| k__Bacteria; p__Actinobacteria; c__Actinobacteria; o__Actinomycetales; f__Actinomycetaceae; g__Mobiluncus; s__ | 0 | 0 | 0 | 7 | 6 | denovo36270 |
| k__Bacteria; p__Actinobacteria; c__Actinobacteria; o__Actinomycetales; f__Actinomycetaceae; g__Varibaculum; s__ | 0 | 0 | 0 | 12 | 1 | denovo18346 |
| k__Bacteria; p__Actinobacteria; c__Actinobacteria; o__Actinomycetales; f__Brevibacteriaceae; g__Brevibacterium; s__aureum | 0 | 1 | 0 | 2 | 1253 | denovo43669 |
| k__Bacteria; p__Actinobacteria; c__Actinobacteria; o__Actinomycetales; f__Brevibacteriaceae; g__Brevibacterium; s__paucivorans | 0 | 0 | 0 | 10 | 21 | denovo1445 |
| k__Bacteria; p__Actinobacteria; c__Actinobacteria; o__Actinomycetales; f__Cellulomonadaceae; g__Cellulomonas; s__ | 0 | 0 | 0 | 1 | 362 | denovo13401 |
| k__Bacteria; p__Actinobacteria; c__Actinobacteria; o__Actinomycetales; f__Corynebacteriaceae; g__Corynebacterium | 0 | 0 | 2 | 23 | 15 | denovo35649 |
| k__Bacteria; p__Actinobacteria; c__Actinobacteria; o__Actinomycetales; f__Corynebacteriaceae; g__Corynebacterium; s__ | 4 | 0 | 30 | 217 | 381 | denovo17582 |
| k__Bacteria; p__Actinobacteria; c__Actinobacteria; o__Actinomycetales; f__Corynebacteriaceae; g__Corynebacterium; s__durum | 0 | 0 | 0 | 2 | 3 | denovo28110 |
| k__Bacteria; p__Actinobacteria; c__Actinobacteria; o__Actinomycetales; f__Corynebacteriaceae; g__Corynebacterium; s__kroppenstedtii | 0 | 0 | 3 | 26 | 45 | denovo30323 |
| k__Bacteria; p__Actinobacteria; c__Actinobacteria; o__Actinomycetales; f__Dermabacteraceae; g__; s__ | 0 | 0 | 0 | 0 | 5 | denovo41208 |
| k__Bacteria; p__Actinobacteria; c__Actinobacteria; o__Actinomycetales; f__Dermabacteraceae; g__Brachybacterium; s__ | 0 | 0 | 0 | 7 | 80 | denovo43431 |
| k__Bacteria; p__Actinobacteria; c__Actinobacteria; o__Actinomycetales; f__Dietziaceae | 0 | 0 | 0 | 0 | 5 | denovo23932 |
| k__Bacteria; p__Actinobacteria; c__Actinobacteria; o__Actinomycetales; f__Geodermatophilaceae | 0 | 0 | 1 | 2 | 1 | denovo16235 |
| k__Bacteria; p__Actinobacteria; c__Actinobacteria; o__Actinomycetales; f__Geodermatophilaceae; g__; s__ | 1 | 0 | 14 | 15 | 18 | denovo3485 |
| k__Bacteria; p__Actinobacteria; c__Actinobacteria; o__Actinomycetales; f__Geodermatophilaceae; g__Geodermatophilus; s__obscurus | 0 | 0 | 0 | 2 | 7 | denovo42913 |
| k__Bacteria; p__Actinobacteria; c__Actinobacteria; o__Actinomycetales; f__Glycomycetaceae; g__Glycomyces; s__ | 0 | 0 | 2 | 0 | 2 | denovo24081 |
| k__Bacteria; p__Actinobacteria; c__Actinobacteria; o__Actinomycetales; f__Gordoniaceae; g__Gordonia; s__ | 0 | 0 | 0 | 0 | 24 | denovo29364 |
| k__Bacteria; p__Actinobacteria; c__Actinobacteria; o__Actinomycetales; f__Intrasporangiaceae | 0 | 0 | 14 | 25 | 61 | denovo20374 |
| k__Bacteria; p__Actinobacteria; c__Actinobacteria; o__Actinomycetales; f__Intrasporangiaceae; g__Serinicoccus; s__ | 0 | 0 | 0 | 0 | 4 | denovo23026 |
| k__Bacteria; p__Actinobacteria; c__Actinobacteria; o__Actinomycetales; f__Microbacteriaceae | 0 | 0 | 1 | 4 | 25 | denovo16718 |
| k__Bacteria; p__Actinobacteria; c__Actinobacteria; o__Actinomycetales; f__Microbacteriaceae; g__Agromyces; s__ | 0 | 0 | 0 | 0 | 14 | denovo16099 |
| k__Bacteria; p__Actinobacteria; c__Actinobacteria; o__Actinomycetales; f__Microbacteriaceae; g__Leucobacter; s__ | 0 | 0 | 0 | 0 | 51 | denovo19505 |
| k__Bacteria; p__Actinobacteria; c__Actinobacteria; o__Actinomycetales; f__Microbacteriaceae; g__Microbacterium; s__ | 0 | 0 | 0 | 2 | 10 | denovo21939 |
| k__Bacteria; p__Actinobacteria; c__Actinobacteria; o__Actinomycetales; f__Microbacteriaceae; g__Rathayibacter; s__ | 0 | 0 | 0 | 0 | 7 | denovo22433 |
| k__Bacteria; p__Actinobacteria; c__Actinobacteria; o__Actinomycetales; f__Micrococcaceae; g__Arthrobacter; s__psychrolactophilus | 0 | 0 | 0 | 0 | 21 | denovo39170 |
| k__Bacteria; p__Actinobacteria; c__Actinobacteria; o__Actinomycetales; f__Micrococcaceae; g__Kocuria | 0 | 0 | 0 | 16 | 26 | denovo22845 |
| k__Bacteria; p__Actinobacteria; c__Actinobacteria; o__Actinomycetales; f__Micrococcaceae; g__Micrococcus; s__ | 0 | 0 | 10 | 11 | 56 | denovo1365 |
| k__Bacteria; p__Actinobacteria; c__Actinobacteria; o__Actinomycetales; f__Micrococcaceae; g__Rothia; s__dentocariosa | 0 | 0 | 1 | 32 | 355 | denovo9699 |
| k__Bacteria; p__Actinobacteria; c__Actinobacteria; o__Actinomycetales; f__Micrococcaceae; g__Rothia; s__mucilaginosa | 1 | 0 | 5 | 89 | 190 | denovo41962 |
| k__Bacteria; p__Actinobacteria; c__Actinobacteria; o__Actinomycetales; f__Micrococcaceae; g__Sinomonas; s__ | 1 | 0 | 3 | 220 | 17 | denovo12120 |
| k__Bacteria; p__Actinobacteria; c__Actinobacteria; o__Actinomycetales; f__Mycobacteriaceae; g__Mycobacterium; s__ | 0 | 0 | 1 | 4 | 6217 | denovo36433 |
| k__Bacteria; p__Actinobacteria; c__Actinobacteria; o__Actinomycetales; f__Mycobacteriaceae; g__Mycobacterium; s__llatzerense | 0 | 0 | 0 | 0 | 29 | denovo12746 |
| k__Bacteria; p__Actinobacteria; c__Actinobacteria; o__Actinomycetales; f__Nocardiaceae; g__Rhodococcus; s__ | 0 | 0 | 0 | 0 | 44 | denovo37887 |
| k__Bacteria; p__Actinobacteria; c__Actinobacteria; o__Actinomycetales; f__Nocardiaceae; g__Rhodococcus; s__fascians | 0 | 0 | 0 | 0 | 19 | denovo22058 |
| k__Bacteria; p__Actinobacteria; c__Actinobacteria; o__Actinomycetales; f__Nocardioidaceae; g__; s__ | 0 | 1 | 26 | 32 | 15 | denovo6349 |
| k__Bacteria; p__Actinobacteria; c__Actinobacteria; o__Actinomycetales; f__Nocardioidaceae; g__Aeromicrobium; s__ | 0 | 0 | 0 | 0 | 6 | denovo4328 |
| k__Bacteria; p__Actinobacteria; c__Actinobacteria; o__Actinomycetales; f__Nocardioidaceae; g__Nocardioides; s__ | 0 | 0 | 0 | 0 | 6 | denovo3833 |
| k__Bacteria; p__Actinobacteria; c__Actinobacteria; o__Actinomycetales; f__Propionibacteriaceae; g__Propionibacterium; s__acnes | 1 | 0 | 2 | 25 | 35 | denovo34796 |
| k__Bacteria; p__Actinobacteria; c__Actinobacteria; o__Actinomycetales; f__Pseudonocardiaceae; g__Actinomycetospora; s__ | 0 | 0 | 0 | 0 | 6 | denovo4817 |
| k__Bacteria; p__Actinobacteria; c__Actinobacteria; o__Actinomycetales; f__Pseudonocardiaceae; g__Pseudonocardia; s__ | 0 | 0 | 0 | 1 | 141 | denovo1624 |
| k__Bacteria; p__Actinobacteria; c__Actinobacteria; o__Bifidobacteriales; f__Bifidobacteriaceae; g__Bifidobacterium | 0 | 0 | 0 | 14 | 7 | denovo20043 |
| k__Bacteria; p__Actinobacteria; c__Actinobacteria; o__Bifidobacteriales; f__Bifidobacteriaceae; g__Bifidobacterium; s__ | 3 | 3 | 129 | 1048 | 491 | denovo17101 |
| k__Bacteria; p__Actinobacteria; c__Actinobacteria; o__Bifidobacteriales; f__Bifidobacteriaceae; g__Bifidobacterium; s__adolescentis | 0 | 0 | 1 | 9 | 0 | denovo28470 |
| k__Bacteria; p__Actinobacteria; c__Actinobacteria; o__Bifidobacteriales; f__Bifidobacteriaceae; g__Gardnerella; s__ | 12 | 0 | 14 | 125 | 97 | denovo24126 |
| k__Bacteria; p__Actinobacteria; c__Coriobacteriia; o__Coriobacteriales; f__Coriobacteriaceae | 0 | 0 | 0 | 14 | 0 | denovo30760 |
| k__Bacteria; p__Actinobacteria; c__Coriobacteriia; o__Coriobacteriales; f__Coriobacteriaceae; g__; s__ | 0 | 0 | 2 | 22 | 3 | denovo35534 |
| k__Bacteria; p__Actinobacteria; c__Coriobacteriia; o__Coriobacteriales; f__Coriobacteriaceae; g__Adlercreutzia; s__ | 0 | 0 | 19 | 110 | 15 | denovo24832 |
| k__Bacteria; p__Actinobacteria; c__Coriobacteriia; o__Coriobacteriales; f__Coriobacteriaceae; g__Atopobium; s__ | 2 | 1 | 14 | 382 | 202 | denovo31476 |
| k__Bacteria; p__Actinobacteria; c__Coriobacteriia; o__Coriobacteriales; f__Coriobacteriaceae; g__Atopobium; s__vaginae | 1 | 0 | 1 | 22 | 19 | denovo3319 |
| k__Bacteria; p__Actinobacteria; c__Coriobacteriia; o__Coriobacteriales; f__Coriobacteriaceae; g__Collinsella; s__ | 0 | 0 | 0 | 6 | 0 | denovo14143 |
| k__Bacteria; p__Actinobacteria; c__Coriobacteriia; o__Coriobacteriales; f__Coriobacteriaceae; g__Collinsella; s__aerofaciens | 2 | 1 | 13 | 280 | 85 | denovo34391 |
| k__Bacteria; p__Actinobacteria; c__Coriobacteriia; o__Coriobacteriales; f__Coriobacteriaceae; g__Collinsella; s__stercoris | 0 | 0 | 2 | 3 | 2 | denovo6757 |
| k__Bacteria; p__Actinobacteria; c__Coriobacteriia; o__Coriobacteriales; f__Coriobacteriaceae; g__Eggerthella; s__lenta | 0 | 0 | 5 | 15 | 0 | denovo11819 |
| k__Bacteria; p__Armatimonadetes; c__[Fimbriimonadia]; o__[Fimbriimonadales]; f__[Fimbriimonadaceae]; g__Fimbriimonas; s__ | 0 | 0 | 0 | 7 | 0 | denovo9726 |
| k__Bacteria; p__Bacteroidetes | 1401 | 4237 | 3211 | 333 | 42 | denovo7215 |
| k__Bacteria; p__Bacteroidetes; c__; o__; f__; g__; s__ | 0 | 0 | 2 | 10 | 36 | denovo8520 |
| k__Bacteria; p__Bacteroidetes; c__[Rhodothermi]; o__[Rhodothermales]; f__[Balneolaceae]; g__Balneola; s__ | 0 | 0 | 2 | 62 | 8 | denovo43520 |
| k__Bacteria; p__Bacteroidetes; c__[Saprospirae]; o__[Saprospirales] | 177 | 0 | 0 | 0 | 0 | denovo37826 |
| k__Bacteria; p__Bacteroidetes; c__[Saprospirae]; o__[Saprospirales]; f__; g__; s__ | 531 | 0 | 0 | 0 | 2 | denovo11742 |
| k__Bacteria; p__Bacteroidetes; c__[Saprospirae]; o__[Saprospirales]; f__Chitinophagaceae | 60 | 0 | 0 | 4 | 114 | denovo9944 |
| k__Bacteria; p__Bacteroidetes; c__[Saprospirae]; o__[Saprospirales]; f__Chitinophagaceae; g__; s__ | 1206 | 0 | 4 | 22 | 20 | denovo31924 |
| k__Bacteria; p__Bacteroidetes; c__[Saprospirae]; o__[Saprospirales]; f__Chitinophagaceae; g__Sediminibacterium; s__ | 0 | 0 | 3 | 2 | 22 | denovo44449 |
| k__Bacteria; p__Bacteroidetes; c__[Saprospirae]; o__[Saprospirales]; f__Saprospiraceae | 1058 | 0 | 0 | 0 | 3 | denovo34151 |
| k__Bacteria; p__Bacteroidetes; c__[Saprospirae]; o__[Saprospirales]; f__Saprospiraceae; g__; s__ | 21682 | 3 | 3 | 9 | 96 | denovo27338 |
| k__Bacteria; p__Bacteroidetes; c__[Saprospirae]; o__[Saprospirales]; f__Saprospiraceae; g__Lewinella | 202 | 0 | 0 | 0 | 2 | denovo4957 |
| k__Bacteria; p__Bacteroidetes; c__[Saprospirae]; o__[Saprospirales]; f__Saprospiraceae; g__Lewinella; s__ | 8 | 0 | 5 | 0 | 5 | denovo14385 |
| k__Bacteria; p__Bacteroidetes; c__[Saprospirae]; o__[Saprospirales]; f__Saprospiraceae; g__Portibacter; s__lacus | 752 | 0 | 0 | 0 | 1 | denovo31128 |
| k__Bacteria; p__Bacteroidetes; c__[Saprospirae]; o__[Saprospirales]; f__Saprospiraceae; g__Saprospira; s__ | 268 | 0 | 0 | 0 | 6 | denovo32700 |
| k__Bacteria; p__Bacteroidetes; c__Bacteroidia; o__Bacteroidales | 253 | 1175 | 1306 | 128 | 17 | denovo23554 |
| k__Bacteria; p__Bacteroidetes; c__Bacteroidia; o__Bacteroidales; f__; g__; s__ | 14 | 1536 | 1436 | 349 | 27 | denovo27331 |
| k__Bacteria; p__Bacteroidetes; c__Bacteroidia; o__Bacteroidales; f__[Barnesiellaceae]; g__; s__ | 0 | 0 | 54 | 30 | 22 | denovo17156 |
| k__Bacteria; p__Bacteroidetes; c__Bacteroidia; o__Bacteroidales; f__[Odoribacteraceae]; g__Butyricimonas; s__ | 0 | 0 | 0 | 29 | 1 | denovo9313 |
| k__Bacteria; p__Bacteroidetes; c__Bacteroidia; o__Bacteroidales; f__[Odoribacteraceae]; g__Odoribacter; s__ | 1 | 0 | 127 | 506 | 451 | denovo25190 |
| k__Bacteria; p__Bacteroidetes; c__Bacteroidia; o__Bacteroidales; f__[Paraprevotellaceae]; g__; s__ | 0 | 0 | 4 | 83 | 0 | denovo27024 |
| k__Bacteria; p__Bacteroidetes; c__Bacteroidia; o__Bacteroidales; f__[Paraprevotellaceae]; g__[Prevotella]; s__ | 10 | 3 | 197 | 3594 | 2036 | denovo31073 |
| k__Bacteria; p__Bacteroidetes; c__Bacteroidia; o__Bacteroidales; f__[Paraprevotellaceae]; g__[Prevotella]; s__tannerae | 0 | 0 | 1 | 12 | 6 | denovo37096 |
| k__Bacteria; p__Bacteroidetes; c__Bacteroidia; o__Bacteroidales; f__[Paraprevotellaceae]; g__Paraprevotella; s__ | 1 | 0 | 6 | 47 | 17 | denovo14488 |
| k__Bacteria; p__Bacteroidetes; c__Bacteroidia; o__Bacteroidales; f__Bacteroidaceae; g__Bacteroides | 1 | 0 | 85 | 372 | 229 | denovo14621 |
| k__Bacteria; p__Bacteroidetes; c__Bacteroidia; o__Bacteroidales; f__Bacteroidaceae; g__Bacteroides; s__ | 4 | 6 | 210 | 1014 | 344 | denovo844 |
| k__Bacteria; p__Bacteroidetes; c__Bacteroidia; o__Bacteroidales; f__Bacteroidaceae; g__Bacteroides; s__caccae | 0 | 0 | 18 | 58 | 31 | denovo22402 |
| k__Bacteria; p__Bacteroidetes; c__Bacteroidia; o__Bacteroidales; f__Bacteroidaceae; g__Bacteroides; s__eggerthii | 2 | 0 | 7 | 49 | 46 | denovo26786 |
| k__Bacteria; p__Bacteroidetes; c__Bacteroidia; o__Bacteroidales; f__Bacteroidaceae; g__Bacteroides; s__fragilis | 0 | 0 | 11 | 61 | 21 | denovo29401 |
| k__Bacteria; p__Bacteroidetes; c__Bacteroidia; o__Bacteroidales; f__Bacteroidaceae; g__Bacteroides; s__ovatus | 8 | 2 | 540 | 1501 | 534 | denovo42642 |
| k__Bacteria; p__Bacteroidetes; c__Bacteroidia; o__Bacteroidales; f__Bacteroidaceae; g__Bacteroides; s__plebeius | 1 | 0 | 0 | 4 | 1 | denovo39465 |
| k__Bacteria; p__Bacteroidetes; c__Bacteroidia; o__Bacteroidales; f__Bacteroidaceae; g__Bacteroides; s__uniformis | 4 | 5 | 107 | 358 | 293 | denovo15998 |
| k__Bacteria; p__Bacteroidetes; c__Bacteroidia; o__Bacteroidales; f__Marinilabiaceae; g__; s__ | 0 | 0 | 3 | 0 | 4 | denovo138 |
| k__Bacteria; p__Bacteroidetes; c__Bacteroidia; o__Bacteroidales; f__Marinilabiaceae; g__Cytophaga; s__fermentans | 0 | 58 | 11 | 0 | 0 | denovo10367 |
| k__Bacteria; p__Bacteroidetes; c__Bacteroidia; o__Bacteroidales; f__Porphyromonadaceae; g__Paludibacter; s__ | 0 | 0 | 0 | 2 | 4 | denovo38145 |
| k__Bacteria; p__Bacteroidetes; c__Bacteroidia; o__Bacteroidales; f__Porphyromonadaceae; g__Parabacteroides | 1 | 5 | 14 | 396 | 54 | denovo12896 |
| k__Bacteria; p__Bacteroidetes; c__Bacteroidia; o__Bacteroidales; f__Porphyromonadaceae; g__Parabacteroides; s__ | 1 | 2 | 20 | 85 | 34 | denovo30617 |
| k__Bacteria; p__Bacteroidetes; c__Bacteroidia; o__Bacteroidales; f__Porphyromonadaceae; g__Parabacteroides; s__distasonis | 0 | 0 | 6 | 36 | 18 | denovo36004 |
| k__Bacteria; p__Bacteroidetes; c__Bacteroidia; o__Bacteroidales; f__Porphyromonadaceae; g__Porphyromonas; s__ | 1 | 1 | 33 | 924 | 667 | denovo35427 |
| k__Bacteria; p__Bacteroidetes; c__Bacteroidia; o__Bacteroidales; f__Porphyromonadaceae; g__Porphyromonas; s__endodontalis | 2 | 0 | 3 | 45 | 18 | denovo3204 |
| k__Bacteria; p__Bacteroidetes; c__Bacteroidia; o__Bacteroidales; f__Porphyromonadaceae; g__Tannerella; s__ | 0 | 0 | 1 | 14 | 12 | denovo3970 |
| k__Bacteria; p__Bacteroidetes; c__Bacteroidia; o__Bacteroidales; f__Prevotellaceae; g__Prevotella | 0 | 0 | 2 | 71 | 10 | denovo37950 |
| k__Bacteria; p__Bacteroidetes; c__Bacteroidia; o__Bacteroidales; f__Prevotellaceae; g__Prevotella; s__ | 51 | 12 | 616 | 9667 | 4249 | denovo32860 |
| k__Bacteria; p__Bacteroidetes; c__Bacteroidia; o__Bacteroidales; f__Prevotellaceae; g__Prevotella; s__intermedia | 0 | 0 | 0 | 14 | 1 | denovo6286 |
| k__Bacteria; p__Bacteroidetes; c__Bacteroidia; o__Bacteroidales; f__Prevotellaceae; g__Prevotella; s__nanceiensis | 2 | 0 | 10 | 301 | 400 | denovo24527 |
| k__Bacteria; p__Bacteroidetes; c__Bacteroidia; o__Bacteroidales; f__Prevotellaceae; g__Prevotella; s__nigrescens | 0 | 0 | 0 | 41 | 60 | denovo19960 |
| k__Bacteria; p__Bacteroidetes; c__Bacteroidia; o__Bacteroidales; f__Prevotellaceae; g__Prevotella; s__stercorea | 0 | 0 | 5 | 120 | 5 | denovo19562 |
| k__Bacteria; p__Bacteroidetes; c__Bacteroidia; o__Bacteroidales; f__Rikenellaceae | 0 | 0 | 1 | 8 | 1 | denovo44410 |
| k__Bacteria; p__Bacteroidetes; c__Bacteroidia; o__Bacteroidales; f__Rikenellaceae; g__; s__ | 5 | 10 | 263 | 1841 | 873 | denovo32502 |
| k__Bacteria; p__Bacteroidetes; c__Bacteroidia; o__Bacteroidales; f__Rikenellaceae; g__AF12; s__ | 0 | 0 | 1 | 4 | 0 | denovo32655 |
| k__Bacteria; p__Bacteroidetes; c__Bacteroidia; o__Bacteroidales; f__Rikenellaceae; g__Alistipes; s__massiliensis | 0 | 0 | 0 | 4 | 2 | denovo36432 |
| k__Bacteria; p__Bacteroidetes; c__Bacteroidia; o__Bacteroidales; f__Rikenellaceae; g__Rikenella; s__ | 0 | 0 | 0 | 60 | 3 | denovo12080 |
| k__Bacteria; p__Bacteroidetes; c__Bacteroidia; o__Bacteroidales; f__S24-7; g__; s__ | 12 | 8 | 738 | 5714 | 2140 | denovo36739 |
| k__Bacteria; p__Bacteroidetes; c__Bacteroidia; o__Bacteroidales; f__SB-1; g__; s__ | 0 | 571 | 1460 | 78 | 1 | denovo34530 |
| k__Bacteria; p__Bacteroidetes; c__Cytophagia; o__Cytophagales | 3 | 0 | 0 | 0 | 4 | denovo8579 |
| k__Bacteria; p__Bacteroidetes; c__Cytophagia; o__Cytophagales; f__[Amoebophilaceae]; g__Candidatus Cardinium; s__ | 3 | 0 | 0 | 0 | 4 | denovo4143 |
| k__Bacteria; p__Bacteroidetes; c__Cytophagia; o__Cytophagales; f__[Amoebophilaceae]; g__SC3-56; s__ | 32 | 0 | 0 | 0 | 0 | denovo34783 |
| k__Bacteria; p__Bacteroidetes; c__Cytophagia; o__Cytophagales; f__Cytophagaceae; g__Adhaeribacter; s__ | 0 | 0 | 0 | 13 | 0 | denovo34936 |
| k__Bacteria; p__Bacteroidetes; c__Cytophagia; o__Cytophagales; f__Cytophagaceae; g__Dyadobacter; s__ | 0 | 0 | 0 | 3 | 29 | denovo28756 |
| k__Bacteria; p__Bacteroidetes; c__Cytophagia; o__Cytophagales; f__Cytophagaceae; g__Hymenobacter; s__ | 0 | 0 | 0 | 0 | 17 | denovo39667 |
| k__Bacteria; p__Bacteroidetes; c__Cytophagia; o__Cytophagales; f__Cytophagaceae; g__Leadbetterella; s__ | 0 | 0 | 0 | 0 | 5 | denovo8850 |
| k__Bacteria; p__Bacteroidetes; c__Cytophagia; o__Cytophagales; f__Cytophagaceae; g__Siphonobacter; s__aquaeclarae | 0 | 0 | 0 | 1 | 245 | denovo4027 |
| k__Bacteria; p__Bacteroidetes; c__Cytophagia; o__Cytophagales; f__Cytophagaceae; g__Spirosoma; s__ | 0 | 0 | 0 | 0 | 29 | denovo32693 |
| k__Bacteria; p__Bacteroidetes; c__Cytophagia; o__Cytophagales; f__Flammeovirgaceae | 57 | 0 | 0 | 0 | 5 | denovo27901 |
| k__Bacteria; p__Bacteroidetes; c__Cytophagia; o__Cytophagales; f__Flammeovirgaceae; g__; s__ | 74 | 0 | 0 | 0 | 3 | denovo30819 |
| k__Bacteria; p__Bacteroidetes; c__Cytophagia; o__Cytophagales; f__Flammeovirgaceae; g__Flexibacter; s__ | 13 | 0 | 0 | 0 | 3 | denovo42638 |
| k__Bacteria; p__Bacteroidetes; c__Cytophagia; o__Cytophagales; f__Flammeovirgaceae; g__JTB248; s__ | 26 | 0 | 0 | 0 | 1 | denovo3404 |
| k__Bacteria; p__Bacteroidetes; c__Cytophagia; o__Cytophagales; f__Flammeovirgaceae; g__Reichenbachiella; s__ | 0 | 0 | 0 | 25 | 0 | denovo16215 |
| k__Bacteria; p__Bacteroidetes; c__Cytophagia; o__Cytophagales; f__Flammeovirgaceae; g__Roseivirga; s__ | 5 | 0 | 0 | 0 | 0 | denovo4002 |
| k__Bacteria; p__Bacteroidetes; c__Flavobacteriia | 57 | 0 | 0 | 0 | 0 | denovo2558 |
| k__Bacteria; p__Bacteroidetes; c__Flavobacteriia; o__Flavobacteriales | 169 | 22813 | 5292 | 844 | 174 | denovo40082 |
| k__Bacteria; p__Bacteroidetes; c__Flavobacteriia; o__Flavobacteriales; f__; g__; s__ | 911 | 13323 | 6900 | 356 | 137 | denovo13116 |
| k__Bacteria; p__Bacteroidetes; c__Flavobacteriia; o__Flavobacteriales; f__[Weeksellaceae]; g__; s__ | 4 | 0 | 2 | 162 | 85 | denovo12792 |
| k__Bacteria; p__Bacteroidetes; c__Flavobacteriia; o__Flavobacteriales; f__[Weeksellaceae]; g__Chryseobacterium; s__ | 1 | 4 | 10 | 53 | 310 | denovo32106 |
| k__Bacteria; p__Bacteroidetes; c__Flavobacteriia; o__Flavobacteriales; f__[Weeksellaceae]; g__Cloacibacterium; s__ | 0 | 0 | 5 | 6 | 86 | denovo24256 |
| k__Bacteria; p__Bacteroidetes; c__Flavobacteriia; o__Flavobacteriales; f__Cryomorphaceae | 76 | 0 | 0 | 0 | 0 | denovo32645 |
| k__Bacteria; p__Bacteroidetes; c__Flavobacteriia; o__Flavobacteriales; f__Cryomorphaceae; g__; s__ | 204 | 0 | 0 | 0 | 66 | denovo21311 |
| k__Bacteria; p__Bacteroidetes; c__Flavobacteriia; o__Flavobacteriales; f__Cryomorphaceae; g__Crocinitomix; s__ | 265 | 0 | 2 | 11 | 8 | denovo37907 |
| k__Bacteria; p__Bacteroidetes; c__Flavobacteriia; o__Flavobacteriales; f__Cryomorphaceae; g__Cryomorpha; s__ | 2 | 1 | 0 | 0 | 0 | denovo18854 |
| k__Bacteria; p__Bacteroidetes; c__Flavobacteriia; o__Flavobacteriales; f__Cryomorphaceae; g__Fluviicola; s__ | 923 | 0 | 0 | 0 | 19 | denovo17218 |
| k__Bacteria; p__Bacteroidetes; c__Flavobacteriia; o__Flavobacteriales; f__Flavobacteriaceae | 4992 | 173 | 516 | 104 | 75 | denovo34159 |
| k__Bacteria; p__Bacteroidetes; c__Flavobacteriia; o__Flavobacteriales; f__Flavobacteriaceae; g__; s__ | 562 | 0 | 5 | 21 | 330 | denovo20144 |
| k__Bacteria; p__Bacteroidetes; c__Flavobacteriia; o__Flavobacteriales; f__Flavobacteriaceae; g__Aquimarina | 226 | 0 | 0 | 0 | 0 | denovo27802 |
| k__Bacteria; p__Bacteroidetes; c__Flavobacteriia; o__Flavobacteriales; f__Flavobacteriaceae; g__Aquimarina; s__ | 405 | 0 | 0 | 0 | 5 | denovo41633 |
| k__Bacteria; p__Bacteroidetes; c__Flavobacteriia; o__Flavobacteriales; f__Flavobacteriaceae; g__Capnocytophaga; s__ | 1 | 0 | 29 | 609 | 441 | denovo13247 |
| k__Bacteria; p__Bacteroidetes; c__Flavobacteriia; o__Flavobacteriales; f__Flavobacteriaceae; g__Capnocytophaga; s__ochracea | 0 | 0 | 1 | 8 | 24 | denovo36158 |
| k__Bacteria; p__Bacteroidetes; c__Flavobacteriia; o__Flavobacteriales; f__Flavobacteriaceae; g__Cellulophaga | 24 | 0 | 0 | 0 | 0 | denovo32235 |
| k__Bacteria; p__Bacteroidetes; c__Flavobacteriia; o__Flavobacteriales; f__Flavobacteriaceae; g__Cellulophaga; s__ | 6 | 0 | 0 | 0 | 0 | denovo5513 |
| k__Bacteria; p__Bacteroidetes; c__Flavobacteriia; o__Flavobacteriales; f__Flavobacteriaceae; g__Cellulophaga; s__lytica | 7 | 0 | 0 | 0 | 0 | denovo4249 |
| k__Bacteria; p__Bacteroidetes; c__Flavobacteriia; o__Flavobacteriales; f__Flavobacteriaceae; g__Croceitalea; s__dokdonensis | 0 | 0 | 0 | 0 | 8 | denovo6392 |
| k__Bacteria; p__Bacteroidetes; c__Flavobacteriia; o__Flavobacteriales; f__Flavobacteriaceae; g__Flavobacterium | 39 | 0 | 0 | 0 | 0 | denovo978 |
| k__Bacteria; p__Bacteroidetes; c__Flavobacteriia; o__Flavobacteriales; f__Flavobacteriaceae; g__Flavobacterium; s__ | 21 | 0 | 6 | 4 | 361 | denovo24818 |
| k__Bacteria; p__Bacteroidetes; c__Flavobacteriia; o__Flavobacteriales; f__Flavobacteriaceae; g__Flavobacterium; s__gelidilacus | 0 | 0 | 3 | 0 | 5 | denovo834 |
| k__Bacteria; p__Bacteroidetes; c__Flavobacteriia; o__Flavobacteriales; f__Flavobacteriaceae; g__Flavobacterium; s__succinicans | 0 | 0 | 0 | 0 | 4 | denovo37124 |
| k__Bacteria; p__Bacteroidetes; c__Flavobacteriia; o__Flavobacteriales; f__Flavobacteriaceae; g__Kordia; s__ | 523 | 1 | 0 | 0 | 50 | denovo1298 |
| k__Bacteria; p__Bacteroidetes; c__Flavobacteriia; o__Flavobacteriales; f__Flavobacteriaceae; g__Krokinobacter; s__genikus | 199 | 0 | 0 | 1 | 15 | denovo33636 |
| k__Bacteria; p__Bacteroidetes; c__Flavobacteriia; o__Flavobacteriales; f__Flavobacteriaceae; g__Lutibacter; s__maritimus | 0 | 0 | 0 | 0 | 4 | denovo14148 |
| k__Bacteria; p__Bacteroidetes; c__Flavobacteriia; o__Flavobacteriales; f__Flavobacteriaceae; g__Lutimonas; s__ | 16 | 7 | 13 | 1 | 4 | denovo1342 |
| k__Bacteria; p__Bacteroidetes; c__Flavobacteriia; o__Flavobacteriales; f__Flavobacteriaceae; g__Maribacter; s__ | 6365 | 0 | 1 | 1 | 17 | denovo28226 |
| k__Bacteria; p__Bacteroidetes; c__Flavobacteriia; o__Flavobacteriales; f__Flavobacteriaceae; g__Persicivirga; s__xylanidelens | 135 | 0 | 0 | 0 | 4 | denovo28651 |
| k__Bacteria; p__Bacteroidetes; c__Flavobacteriia; o__Flavobacteriales; f__Flavobacteriaceae; g__Polaribacter; s__ | 554 | 0 | 0 | 5 | 273 | denovo35077 |
| k__Bacteria; p__Bacteroidetes; c__Flavobacteriia; o__Flavobacteriales; f__Flavobacteriaceae; g__Polaribacter; s__irgensii | 0 | 0 | 0 | 0 | 22 | denovo9917 |
| k__Bacteria; p__Bacteroidetes; c__Flavobacteriia; o__Flavobacteriales; f__Flavobacteriaceae; g__Psychroserpens; s__ | 910 | 0 | 4 | 8 | 79 | denovo14192 |
| k__Bacteria; p__Bacteroidetes; c__Flavobacteriia; o__Flavobacteriales; f__Flavobacteriaceae; g__Sediminicola; s__ | 0 | 0 | 0 | 0 | 18 | denovo14497 |
| k__Bacteria; p__Bacteroidetes; c__Flavobacteriia; o__Flavobacteriales; f__Flavobacteriaceae; g__Tenacibaculum; s__ | 267 | 1 | 0 | 33 | 178 | denovo26251 |
| k__Bacteria; p__Bacteroidetes; c__Flavobacteriia; o__Flavobacteriales; f__Flavobacteriaceae; g__Ulvibacter; s__ | 1153 | 2 | 0 | 1 | 14 | denovo8202 |
| k__Bacteria; p__Bacteroidetes; c__Flavobacteriia; o__Flavobacteriales; f__Flavobacteriaceae; g__Winogradskyella | 5 | 0 | 0 | 0 | 0 | denovo6883 |
| k__Bacteria; p__Bacteroidetes; c__Flavobacteriia; o__Flavobacteriales; f__Flavobacteriaceae; g__Winogradskyella; s__ | 220 | 0 | 1 | 2 | 14 | denovo7866 |
| k__Bacteria; p__Bacteroidetes; c__Flavobacteriia; o__Flavobacteriales; f__NS9; g__; s__ | 0 | 0 | 0 | 0 | 13 | denovo14702 |
| k__Bacteria; p__Bacteroidetes; c__Sphingobacteriia; o__Sphingobacteriales | 88 | 0 | 0 | 0 | 2 | denovo1839 |
| k__Bacteria; p__Bacteroidetes; c__Sphingobacteriia; o__Sphingobacteriales; f__; g__; s__ | 15 | 0 | 0 | 0 | 0 | denovo35607 |
| k__Bacteria; p__Bacteroidetes; c__Sphingobacteriia; o__Sphingobacteriales; f__NS11-12; g__; s__ | 1511 | 0 | 0 | 0 | 16 | denovo4295 |
| k__Bacteria; p__Bacteroidetes; c__Sphingobacteriia; o__Sphingobacteriales; f__Sphingobacteriaceae; g__; s__ | 0 | 0 | 0 | 0 | 9 | denovo3536 |
| k__Bacteria; p__Bacteroidetes; c__Sphingobacteriia; o__Sphingobacteriales; f__Sphingobacteriaceae; g__Pedobacter; s__ | 0 | 0 | 0 | 0 | 59 | denovo1416 |
| k__Bacteria; p__Bacteroidetes; c__Sphingobacteriia; o__Sphingobacteriales; f__Sphingobacteriaceae; g__Sphingobacterium; s__multivorum | 0 | 0 | 0 | 2 | 176 | denovo22236 |
| k__Bacteria; p__Bacteroidetes; c__VC2_1_Bac22; o__; f__; g__; s__ | 0 | 0 | 0 | 0 | 50 | denovo33671 |
| k__Bacteria; p__Chlamydiae; c__Chlamydiia; o__Chlamydiales; f__Chlamydiaceae; g__Chlamydia; s__ | 0 | 0 | 0 | 12 | 0 | denovo38645 |
| k__Bacteria; p__Chlamydiae; c__Chlamydiia; o__Chlamydiales; f__Simkaniaceae | 2 | 21 | 195 | 88 | 2 | denovo40206 |
| k__Bacteria; p__Chlamydiae; c__Chlamydiia; o__Chlamydiales; f__Simkaniaceae; g__; s__ | 0 | 0 | 998 | 0 | 1 | denovo14781 |
| k__Bacteria; p__Chloroflexi; c__Anaerolineae | 211 | 0 | 0 | 0 | 0 | denovo418 |
| k__Bacteria; p__Chloroflexi; c__Anaerolineae; o__Caldilineales; f__Caldilineaceae | 7 | 0 | 0 | 0 | 0 | denovo33047 |
| k__Bacteria; p__Chloroflexi; c__Anaerolineae; o__Caldilineales; f__Caldilineaceae; g__; s__ | 56 | 0 | 0 | 0 | 1 | denovo13201 |
| k__Bacteria; p__Chloroflexi; c__Chloroflexi; o__[Roseiflexales]; f__; g__; s__ | 0 | 0 | 0 | 0 | 9 | denovo13482 |
| k__Bacteria; p__Chloroflexi; c__Chloroflexi; o__[Roseiflexales]; f__[Kouleothrixaceae]; g__; s__ | 0 | 0 | 0 | 4 | 9 | denovo26390 |
| k__Bacteria; p__Cyanobacteria | 0 | 0 | 0 | 0 | 7 | denovo24890 |
| k__Bacteria; p__Cyanobacteria; c__4C0d-2; o__MLE1-12; f__; g__; s__ | 0 | 0 | 0 | 0 | 6 | denovo40713 |
| k__Bacteria; p__Cyanobacteria; c__4C0d-2; o__YS2; f__; g__; s__ | 0 | 0 | 0 | 81 | 6 | denovo3685 |
| k__Bacteria; p__Cyanobacteria; c__Chloroplast | 0 | 5 | 0 | 0 | 0 | denovo8607 |
| k__Bacteria; p__Cyanobacteria; c__Chloroplast; o__; f__; g__; s__ | 64 | 0 | 0 | 0 | 0 | denovo40503 |
| k__Bacteria; p__Cyanobacteria; c__Chloroplast; o__Cryptophyta; f__; g__; s__ | 0 | 0 | 0 | 0 | 3 | denovo17426 |
| k__Bacteria; p__Cyanobacteria; c__Chloroplast; o__Rhodophyta; f__; g__; s__ | 14815 | 306 | 322 | 18 | 40 | denovo1777 |
| k__Bacteria; p__Cyanobacteria; c__Chloroplast; o__Stramenopiles; f__; g__; s__ | 13042 | 24 | 57 | 98 | 964 | denovo13842 |
| k__Bacteria; p__Cyanobacteria; c__Chloroplast; o__Streptophyta; f__; g__; s__ | 0 | 0 | 13 | 133 | 863 | denovo35452 |
| k__Bacteria; p__Cyanobacteria; c__Chloroplast; o__UA01; f__; g__; s__ | 6 | 0 | 0 | 0 | 0 | denovo31873 |
| k__Bacteria; p__Cyanobacteria; c__ML635J-21; o__; f__; g__; s__ | 9 | 0 | 0 | 0 | 54 | denovo19006 |
| k__Bacteria; p__Cyanobacteria; c__Oscillatoriophycideae; o__Chroococcales; f__Xenococcaceae; g__; s__ | 120 | 1 | 0 | 3 | 8 | denovo34297 |
| k__Bacteria; p__Cyanobacteria; c__Oscillatoriophycideae; o__Oscillatoriales; f__Phormidiaceae; g__Phormidium; s__ | 0 | 0 | 0 | 0 | 11 | denovo24601 |
| k__Bacteria; p__Cyanobacteria; c__Synechococcophycideae; o__Pseudanabaenales; f__Pseudanabaenaceae; g__; s__ | 65 | 0 | 0 | 3 | 3 | denovo35739 |
| k__Bacteria; p__Cyanobacteria; c__Synechococcophycideae; o__Pseudanabaenales; f__Pseudanabaenaceae; g__Leptolyngbya; s__ | 0 | 0 | 0 | 4 | 0 | denovo11897 |
| k__Bacteria; p__Cyanobacteria; c__Synechococcophycideae; o__Pseudanabaenales; f__Pseudanabaenaceae; g__Leptolyngbya; s__antarctica | 156 | 0 | 0 | 0 | 0 | denovo35312 |
| k__Bacteria; p__Cyanobacteria; c__Synechococcophycideae; o__Synechococcales; f__Acaryochloridaceae; g__Acaryochloris; s__ | 22 | 0 | 0 | 0 | 4 | denovo38480 |
| k__Bacteria; p__Cyanobacteria; c__Synechococcophycideae; o__Synechococcales; f__Synechococcaceae; g__Prochlorococcus; s__ | 0 | 0 | 0 | 1 | 3 | denovo40934 |
| k__Bacteria; p__Cyanobacteria; c__Synechococcophycideae; o__Synechococcales; f__Synechococcaceae; g__Synechococcus; s__ | 0 | 0 | 5 | 51 | 14 | denovo34028 |
| k__Bacteria; p__Deferribacteres; c__Deferribacteres; o__Deferribacterales; f__Deferribacteraceae; g__Mucispirillum; s__schaedleri | 0 | 0 | 2 | 108 | 14 | denovo22369 |
| k__Bacteria; p__Elusimicrobia; c__Elusimicrobia; o__Elusimicrobiales; f__Elusimicrobiaceae; g__; s__ | 0 | 0 | 0 | 3 | 0 | denovo42922 |
| k__Bacteria; p__Fibrobacteres; c__TG3; o__TG3-2; f__; g__; s__ | 0 | 4 | 0 | 6 | 0 | denovo38406 |
| k__Bacteria; p__Firmicutes | 0 | 24 | 505 | 66 | 34 | denovo32760 |
| k__Bacteria; p__Firmicutes; c__Bacilli | 0 | 117 | 234 | 46 | 16 | denovo329 |
| k__Bacteria; p__Firmicutes; c__Bacilli; o__Bacillales | 0 | 0 | 18 | 3 | 11 | denovo24310 |
| k__Bacteria; p__Firmicutes; c__Bacilli; o__Bacillales; f__[Exiguobacteraceae]; g__; s__ | 0 | 0 | 0 | 5 | 10 | denovo22872 |
| k__Bacteria; p__Firmicutes; c__Bacilli; o__Bacillales; f__[Exiguobacteraceae]; g__Exiguobacterium; s__ | 1 | 0 | 3 | 4 | 1 | denovo21700 |
| k__Bacteria; p__Firmicutes; c__Bacilli; o__Bacillales; f__Alicyclobacillaceae; g__Alicyclobacillus; s__ | 0 | 0 | 11 | 0 | 5 | denovo21296 |
| k__Bacteria; p__Firmicutes; c__Bacilli; o__Bacillales; f__Bacillaceae | 0 | 0 | 19 | 26 | 180 | denovo4455 |
| k__Bacteria; p__Firmicutes; c__Bacilli; o__Bacillales; f__Bacillaceae; g__Bacillus | 0 | 0 | 1 | 0 | 3 | denovo30452 |
| k__Bacteria; p__Firmicutes; c__Bacilli; o__Bacillales; f__Bacillaceae; g__Bacillus; s__ | 0 | 0 | 13 | 14 | 6 | denovo1307 |
| k__Bacteria; p__Firmicutes; c__Bacilli; o__Bacillales; f__Bacillaceae; g__Bacillus; s__cereus | 0 | 0 | 0 | 7 | 5 | denovo684 |
| k__Bacteria; p__Firmicutes; c__Bacilli; o__Bacillales; f__Bacillaceae; g__Bacillus; s__flexus | 0 | 0 | 1 | 4 | 1 | denovo12199 |
| k__Bacteria; p__Firmicutes; c__Bacilli; o__Bacillales; f__Bacillaceae; g__Geobacillus; s__ | 0 | 0 | 0 | 0 | 47 | denovo19962 |
| k__Bacteria; p__Firmicutes; c__Bacilli; o__Bacillales; f__Paenibacillaceae; g__Paenibacillus; s__amylolyticus | 0 | 0 | 0 | 7 | 0 | denovo26678 |
| k__Bacteria; p__Firmicutes; c__Bacilli; o__Bacillales; f__Paenibacillaceae; g__Paenibacillus; s__barengoltzii | 0 | 1 | 0 | 9 | 3 | denovo27505 |
| k__Bacteria; p__Firmicutes; c__Bacilli; o__Bacillales; f__Planococcaceae | 0 | 0 | 1 | 2 | 103 | denovo36626 |
| k__Bacteria; p__Firmicutes; c__Bacilli; o__Bacillales; f__Planococcaceae; g__Chryseomicrobium; s__imtechense | 0 | 0 | 0 | 1 | 10 | denovo20699 |
| k__Bacteria; p__Firmicutes; c__Bacilli; o__Bacillales; f__Planococcaceae; g__Planomicrobium; s__ | 0 | 0 | 0 | 2 | 5 | denovo37923 |
| k__Bacteria; p__Firmicutes; c__Bacilli; o__Bacillales; f__Staphylococcaceae; g__Staphylococcus | 0 | 0 | 3 | 3 | 4 | denovo44520 |
| k__Bacteria; p__Firmicutes; c__Bacilli; o__Bacillales; f__Staphylococcaceae; g__Staphylococcus; s__ | 10 | 1 | 165 | 776 | 896 | denovo29914 |
| k__Bacteria; p__Firmicutes; c__Bacilli; o__Gemellales | 0 | 0 | 2 | 24 | 13 | denovo31222 |
| k__Bacteria; p__Firmicutes; c__Bacilli; o__Gemellales; f__Gemellaceae | 0 | 0 | 0 | 31 | 35 | denovo17108 |
| k__Bacteria; p__Firmicutes; c__Bacilli; o__Gemellales; f__Gemellaceae; g__; s__ | 13 | 2 | 228 | 3928 | 2403 | denovo29206 |
| k__Bacteria; p__Firmicutes; c__Bacilli; o__Gemellales; f__Gemellaceae; g__Gemella; s__ | 0 | 0 | 1 | 4 | 1 | denovo27236 |
| k__Bacteria; p__Firmicutes; c__Bacilli; o__Lactobacillales | 0 | 0 | 1 | 39 | 22 | denovo25469 |
| k__Bacteria; p__Firmicutes; c__Bacilli; o__Lactobacillales; f__Aerococcaceae; g__Abiotrophia; s__ | 0 | 0 | 1 | 12 | 10 | denovo6000 |
| k__Bacteria; p__Firmicutes; c__Bacilli; o__Lactobacillales; f__Aerococcaceae; g__Aerococcus; s__ | 0 | 0 | 1 | 31 | 5 | denovo16659 |
| k__Bacteria; p__Firmicutes; c__Bacilli; o__Lactobacillales; f__Aerococcaceae; g__Alloiococcus; s__ | 0 | 0 | 1 | 7 | 10 | denovo34879 |
| k__Bacteria; p__Firmicutes; c__Bacilli; o__Lactobacillales; f__Aerococcaceae; g__Facklamia; s__ | 0 | 0 | 0 | 5 | 17 | denovo36331 |
| k__Bacteria; p__Firmicutes; c__Bacilli; o__Lactobacillales; f__Carnobacteriaceae | 0 | 0 | 0 | 4 | 0 | denovo34447 |
| k__Bacteria; p__Firmicutes; c__Bacilli; o__Lactobacillales; f__Carnobacteriaceae; g__Granulicatella; s__ | 0 | 0 | 39 | 665 | 648 | denovo5210 |
| k__Bacteria; p__Firmicutes; c__Bacilli; o__Lactobacillales; f__Enterococcaceae | 0 | 0 | 4 | 12 | 6 | denovo30080 |
| k__Bacteria; p__Firmicutes; c__Bacilli; o__Lactobacillales; f__Enterococcaceae; g__Enterococcus | 1 | 0 | 11 | 63 | 20 | denovo31900 |
| k__Bacteria; p__Firmicutes; c__Bacilli; o__Lactobacillales; f__Enterococcaceae; g__Enterococcus; s__casseliflavus | 0 | 0 | 3 | 3 | 2 | denovo26934 |
| k__Bacteria; p__Firmicutes; c__Bacilli; o__Lactobacillales; f__Enterococcaceae; g__Vagococcus; s__ | 0 | 0 | 0 | 5 | 6 | denovo5191 |
| k__Bacteria; p__Firmicutes; c__Bacilli; o__Lactobacillales; f__Lactobacillaceae | 0 | 0 | 0 | 18 | 8 | denovo35166 |
| k__Bacteria; p__Firmicutes; c__Bacilli; o__Lactobacillales; f__Lactobacillaceae; g__Lactobacillus | 5 | 2 | 295 | 1776 | 715 | denovo1031 |
| k__Bacteria; p__Firmicutes; c__Bacilli; o__Lactobacillales; f__Lactobacillaceae; g__Lactobacillus; s__ | 3 | 14 | 110 | 1124 | 887 | denovo12872 |
| k__Bacteria; p__Firmicutes; c__Bacilli; o__Lactobacillales; f__Lactobacillaceae; g__Lactobacillus; s__delbrueckii | 0 | 0 | 4 | 9 | 10 | denovo20874 |
| k__Bacteria; p__Firmicutes; c__Bacilli; o__Lactobacillales; f__Lactobacillaceae; g__Lactobacillus; s__iners | 20 | 4 | 67 | 810 | 694 | denovo22795 |
| k__Bacteria; p__Firmicutes; c__Bacilli; o__Lactobacillales; f__Lactobacillaceae; g__Lactobacillus; s__reuteri | 2 | 0 | 116 | 657 | 1376 | denovo28956 |
| k__Bacteria; p__Firmicutes; c__Bacilli; o__Lactobacillales; f__Lactobacillaceae; g__Lactobacillus; s__vaginalis | 0 | 0 | 2 | 20 | 44 | denovo34493 |
| k__Bacteria; p__Firmicutes; c__Bacilli; o__Lactobacillales; f__Lactobacillaceae; g__Lactobacillus; s__zeae | 0 | 0 | 9 | 13 | 1 | denovo40199 |
| k__Bacteria; p__Firmicutes; c__Bacilli; o__Lactobacillales; f__Lactobacillaceae; g__Pediococcus; s__acidilactici | 0 | 1 | 0 | 12 | 48 | denovo8046 |
| k__Bacteria; p__Firmicutes; c__Bacilli; o__Lactobacillales; f__Leuconostocaceae | 0 | 0 | 0 | 0 | 31 | denovo41735 |
| k__Bacteria; p__Firmicutes; c__Bacilli; o__Lactobacillales; f__Leuconostocaceae; g__; s__ | 0 | 0 | 3 | 5 | 6 | denovo29814 |
| k__Bacteria; p__Firmicutes; c__Bacilli; o__Lactobacillales; f__Leuconostocaceae; g__Leuconostoc; s__ | 0 | 0 | 0 | 0 | 6 | denovo37329 |
| k__Bacteria; p__Firmicutes; c__Bacilli; o__Lactobacillales; f__Streptococcaceae; g__Lactococcus; s__ | 2 | 1 | 10 | 68 | 608 | denovo19506 |
| k__Bacteria; p__Firmicutes; c__Bacilli; o__Lactobacillales; f__Streptococcaceae; g__Lactococcus; s__garvieae | 0 | 0 | 0 | 3 | 4 | denovo44404 |
| k__Bacteria; p__Firmicutes; c__Bacilli; o__Lactobacillales; f__Streptococcaceae; g__Streptococcus | 0 | 0 | 5 | 79 | 81 | denovo35373 |
| k__Bacteria; p__Firmicutes; c__Bacilli; o__Lactobacillales; f__Streptococcaceae; g__Streptococcus; s__ | 7 | 5 | 297 | 1933 | 3018 | denovo16342 |
| k__Bacteria; p__Firmicutes; c__Bacilli; o__Lactobacillales; f__Streptococcaceae; g__Streptococcus; s__anginosus | 0 | 0 | 6 | 6 | 8 | denovo43749 |
| k__Bacteria; p__Firmicutes; c__Bacilli; o__Lactobacillales; f__Streptococcaceae; g__Streptococcus; s__infantis | 0 | 0 | 5 | 37 | 80 | denovo25074 |
| k__Bacteria; p__Firmicutes; c__Bacilli; o__Turicibacterales; f__Turicibacteraceae; g__Turicibacter; s__ | 2 | 7 | 97 | 824 | 259 | denovo40446 |
| k__Bacteria; p__Firmicutes; c__Clostridia | 0 | 9 | 16 | 8 | 2 | denovo6077 |
| k__Bacteria; p__Firmicutes; c__Clostridia; o__Clostridiales | 6 | 257 | 1787 | 2903 | 1138 | denovo3933 |
| k__Bacteria; p__Firmicutes; c__Clostridia; o__Clostridiales; f__; g__; s__ | 10 | 11 | 432 | 2829 | 1350 | denovo16346 |
| k__Bacteria; p__Firmicutes; c__Clostridia; o__Clostridiales; f__[Acidaminobacteraceae]; g__; s__ | 1 | 22 | 64 | 10 | 0 | denovo40680 |
| k__Bacteria; p__Firmicutes; c__Clostridia; o__Clostridiales; f__[Acidaminobacteraceae]; g__Fusibacter; s__ | 0 | 961 | 442 | 147 | 4 | denovo30075 |
| k__Bacteria; p__Firmicutes; c__Clostridia; o__Clostridiales; f__[Mogibacteriaceae] | 0 | 0 | 0 | 5 | 0 | denovo9990 |
| k__Bacteria; p__Firmicutes; c__Clostridia; o__Clostridiales; f__[Mogibacteriaceae]; g__; s__ | 1 | 0 | 12 | 180 | 109 | denovo43022 |
| k__Bacteria; p__Firmicutes; c__Clostridia; o__Clostridiales; f__[Mogibacteriaceae]; g__Mogibacterium; s__ | 2 | 0 | 3 | 13 | 19 | denovo17021 |
| k__Bacteria; p__Firmicutes; c__Clostridia; o__Clostridiales; f__[Tissierellaceae] | 1 | 0 | 115 | 82 | 314 | denovo22543 |
| k__Bacteria; p__Firmicutes; c__Clostridia; o__Clostridiales; f__[Tissierellaceae]; g__; s__ | 0 | 1 | 95 | 30 | 193 | denovo28556 |
| k__Bacteria; p__Firmicutes; c__Clostridia; o__Clostridiales; f__[Tissierellaceae]; g__1-68; s__ | 0 | 0 | 0 | 5 | 15 | denovo35569 |
| k__Bacteria; p__Firmicutes; c__Clostridia; o__Clostridiales; f__[Tissierellaceae]; g__Anaerococcus; s__ | 0 | 0 | 10 | 92 | 151 | denovo41894 |
| k__Bacteria; p__Firmicutes; c__Clostridia; o__Clostridiales; f__[Tissierellaceae]; g__Finegoldia; s__ | 0 | 1 | 3 | 44 | 44 | denovo34805 |
| k__Bacteria; p__Firmicutes; c__Clostridia; o__Clostridiales; f__[Tissierellaceae]; g__Parvimonas; s__ | 0 | 0 | 4 | 184 | 117 | denovo32753 |
| k__Bacteria; p__Firmicutes; c__Clostridia; o__Clostridiales; f__[Tissierellaceae]; g__Peptoniphilus; s__ | 0 | 0 | 8 | 48 | 64 | denovo40555 |
| k__Bacteria; p__Firmicutes; c__Clostridia; o__Clostridiales; f__[Tissierellaceae]; g__ph2; s__ | 0 | 0 | 1 | 7 | 4 | denovo16070 |
| k__Bacteria; p__Firmicutes; c__Clostridia; o__Clostridiales; f__[Tissierellaceae]; g__Tissierella_Soehngenia; s__ | 198 | 45 | 12044 | 8668 | 42350 | denovo23434 |
| k__Bacteria; p__Firmicutes; c__Clostridia; o__Clostridiales; f__[Tissierellaceae]; g__WAL_1855D; s__ | 0 | 0 | 1 | 12 | 15 | denovo1414 |
| k__Bacteria; p__Firmicutes; c__Clostridia; o__Clostridiales; f__Christensenellaceae; g__; s__ | 0 | 0 | 0 | 53 | 2 | denovo30758 |
| k__Bacteria; p__Firmicutes; c__Clostridia; o__Clostridiales; f__Christensenellaceae; g__Christensenella; s__ | 0 | 0 | 0 | 15 | 0 | denovo2321 |
| k__Bacteria; p__Firmicutes; c__Clostridia; o__Clostridiales; f__Clostridiaceae | 1 | 0 | 44 | 184 | 64 | denovo4468 |
| k__Bacteria; p__Firmicutes; c__Clostridia; o__Clostridiales; f__Clostridiaceae; g__Candidatus Arthromitus; s__ | 0 | 0 | 0 | 9 | 4 | denovo8392 |
| k__Bacteria; p__Firmicutes; c__Clostridia; o__Clostridiales; f__Clostridiaceae; g__Clostridium; s__ | 0 | 0 | 1 | 60 | 7 | denovo2158 |
| k__Bacteria; p__Firmicutes; c__Clostridia; o__Clostridiales; f__Clostridiaceae; g__Clostridium; s__butyricum | 0 | 0 | 4 | 2 | 3 | denovo6508 |
| k__Bacteria; p__Firmicutes; c__Clostridia; o__Clostridiales; f__Clostridiaceae; g__Clostridium; s__hiranonis | 0 | 0 | 0 | 3 | 3 | denovo2251 |
| k__Bacteria; p__Firmicutes; c__Clostridia; o__Clostridiales; f__Clostridiaceae; g__Clostridium; s__perfringens | 0 | 0 | 6 | 11 | 16 | denovo41465 |
| k__Bacteria; p__Firmicutes; c__Clostridia; o__Clostridiales; f__Clostridiaceae; g__SMB53; s__ | 0 | 1 | 12 | 187 | 69 | denovo2888 |
| k__Bacteria; p__Firmicutes; c__Clostridia; o__Clostridiales; f__Dehalobacteriaceae; g__Dehalobacterium; s__ | 0 | 2 | 81 | 37 | 22 | denovo10856 |
| k__Bacteria; p__Firmicutes; c__Clostridia; o__Clostridiales; f__Eubacteriaceae; g__Pseudoramibacter_Eubacterium; s__ | 0 | 0 | 5 | 25 | 0 | denovo22201 |
| k__Bacteria; p__Firmicutes; c__Clostridia; o__Clostridiales; f__Lachnospiraceae | 2 | 2 | 205 | 1593 | 686 | denovo13443 |
| k__Bacteria; p__Firmicutes; c__Clostridia; o__Clostridiales; f__Lachnospiraceae; g__; s__ | 4 | 4 | 286 | 2033 | 604 | denovo42979 |
| k__Bacteria; p__Firmicutes; c__Clostridia; o__Clostridiales; f__Lachnospiraceae; g__[Ruminococcus]; s__ | 1 | 3 | 22 | 188 | 42 | denovo36946 |
| k__Bacteria; p__Firmicutes; c__Clostridia; o__Clostridiales; f__Lachnospiraceae; g__[Ruminococcus]; s__gnavus | 4 | 0 | 97 | 584 | 252 | denovo12463 |
| k__Bacteria; p__Firmicutes; c__Clostridia; o__Clostridiales; f__Lachnospiraceae; g__[Ruminococcus]; s__torques | 0 | 2 | 6 | 107 | 32 | denovo14302 |
| k__Bacteria; p__Firmicutes; c__Clostridia; o__Clostridiales; f__Lachnospiraceae; g__Anaerostipes; s__ | 0 | 1 | 0 | 18 | 18 | denovo13088 |
| k__Bacteria; p__Firmicutes; c__Clostridia; o__Clostridiales; f__Lachnospiraceae; g__Blautia | 1 | 0 | 0 | 34 | 23 | denovo30669 |
| k__Bacteria; p__Firmicutes; c__Clostridia; o__Clostridiales; f__Lachnospiraceae; g__Blautia; s__ | 17 | 8 | 123 | 1298 | 422 | denovo21194 |
| k__Bacteria; p__Firmicutes; c__Clostridia; o__Clostridiales; f__Lachnospiraceae; g__Blautia; s__obeum | 3 | 0 | 17 | 256 | 56 | denovo33793 |
| k__Bacteria; p__Firmicutes; c__Clostridia; o__Clostridiales; f__Lachnospiraceae; g__Blautia; s__producta | 1 | 2 | 123 | 121 | 215 | denovo11678 |
| k__Bacteria; p__Firmicutes; c__Clostridia; o__Clostridiales; f__Lachnospiraceae; g__Butyrivibrio; s__ | 0 | 0 | 0 | 30 | 8 | denovo14328 |
| k__Bacteria; p__Firmicutes; c__Clostridia; o__Clostridiales; f__Lachnospiraceae; g__Catonella; s__ | 0 | 0 | 6 | 123 | 103 | denovo82 |
| k__Bacteria; p__Firmicutes; c__Clostridia; o__Clostridiales; f__Lachnospiraceae; g__Clostridium | 0 | 0 | 0 | 4 | 2 | denovo36535 |
| k__Bacteria; p__Firmicutes; c__Clostridia; o__Clostridiales; f__Lachnospiraceae; g__Clostridium; s__citroniae | 0 | 0 | 46 | 46 | 55 | denovo43179 |
| k__Bacteria; p__Firmicutes; c__Clostridia; o__Clostridiales; f__Lachnospiraceae; g__Clostridium; s__colinum | 0 | 0 | 0 | 14 | 5 | denovo38978 |
| k__Bacteria; p__Firmicutes; c__Clostridia; o__Clostridiales; f__Lachnospiraceae; g__Clostridium; s__hathewayi | 0 | 0 | 19 | 20 | 3 | denovo35594 |
| k__Bacteria; p__Firmicutes; c__Clostridia; o__Clostridiales; f__Lachnospiraceae; g__Coprococcus; s__ | 1 | 1 | 58 | 519 | 202 | denovo19030 |
| k__Bacteria; p__Firmicutes; c__Clostridia; o__Clostridiales; f__Lachnospiraceae; g__Coprococcus; s__catus | 0 | 4 | 8 | 46 | 10 | denovo20156 |
| k__Bacteria; p__Firmicutes; c__Clostridia; o__Clostridiales; f__Lachnospiraceae; g__Coprococcus; s__eutactus | 0 | 0 | 1 | 12 | 0 | denovo42071 |
| k__Bacteria; p__Firmicutes; c__Clostridia; o__Clostridiales; f__Lachnospiraceae; g__Defluviitalea; s__saccharophila | 0 | 55 | 95 | 25 | 0 | denovo14289 |
| k__Bacteria; p__Firmicutes; c__Clostridia; o__Clostridiales; f__Lachnospiraceae; g__Dorea; s__ | 0 | 0 | 27 | 170 | 89 | denovo40705 |
| k__Bacteria; p__Firmicutes; c__Clostridia; o__Clostridiales; f__Lachnospiraceae; g__Dorea; s__formicigenerans | 1 | 3 | 8 | 72 | 6 | denovo18260 |
| k__Bacteria; p__Firmicutes; c__Clostridia; o__Clostridiales; f__Lachnospiraceae; g__Lachnobacterium; s__ | 0 | 0 | 1 | 19 | 0 | denovo41203 |
| k__Bacteria; p__Firmicutes; c__Clostridia; o__Clostridiales; f__Lachnospiraceae; g__Lachnospira; s__ | 0 | 0 | 1 | 80 | 6 | denovo33637 |
| k__Bacteria; p__Firmicutes; c__Clostridia; o__Clostridiales; f__Lachnospiraceae; g__Moryella | 0 | 0 | 0 | 24 | 0 | denovo4221 |
| k__Bacteria; p__Firmicutes; c__Clostridia; o__Clostridiales; f__Lachnospiraceae; g__Moryella; s__ | 0 | 0 | 5 | 31 | 152 | denovo43114 |
| k__Bacteria; p__Firmicutes; c__Clostridia; o__Clostridiales; f__Lachnospiraceae; g__Oribacterium; s__ | 1 | 0 | 10 | 194 | 454 | denovo15251 |
| k__Bacteria; p__Firmicutes; c__Clostridia; o__Clostridiales; f__Lachnospiraceae; g__Roseburia; s__ | 4 | 1 | 18 | 461 | 96 | denovo32750 |
| k__Bacteria; p__Firmicutes; c__Clostridia; o__Clostridiales; f__Lachnospiraceae; g__Ruminococcus; s__gauvreauii | 0 | 0 | 0 | 8 | 0 | denovo17382 |
| k__Bacteria; p__Firmicutes; c__Clostridia; o__Clostridiales; f__Lachnospiraceae; g__Shuttleworthia; s__ | 1 | 3 | 5 | 24 | 40 | denovo12970 |
| k__Bacteria; p__Firmicutes; c__Clostridia; o__Clostridiales; f__Peptococcaceae | 0 | 6 | 3 | 0 | 0 | denovo5966 |
| k__Bacteria; p__Firmicutes; c__Clostridia; o__Clostridiales; f__Peptococcaceae; g__; s__ | 0 | 85 | 188 | 3 | 0 | denovo42686 |
| k__Bacteria; p__Firmicutes; c__Clostridia; o__Clostridiales; f__Peptococcaceae; g__Peptococcus; s__ | 0 | 0 | 4 | 53 | 33 | denovo14871 |
| k__Bacteria; p__Firmicutes; c__Clostridia; o__Clostridiales; f__Peptococcaceae; g__rc4-4; s__ | 0 | 0 | 21 | 33 | 52 | denovo34769 |
| k__Bacteria; p__Firmicutes; c__Clostridia; o__Clostridiales; f__Peptostreptococcaceae | 0 | 0 | 1 | 19 | 6 | denovo40263 |
| k__Bacteria; p__Firmicutes; c__Clostridia; o__Clostridiales; f__Peptostreptococcaceae; g__; s__ | 0 | 0 | 0 | 18 | 3 | denovo23162 |
| k__Bacteria; p__Firmicutes; c__Clostridia; o__Clostridiales; f__Peptostreptococcaceae; g__[Clostridium]; s__difficile | 0 | 0 | 3 | 31 | 21 | denovo28649 |
| k__Bacteria; p__Firmicutes; c__Clostridia; o__Clostridiales; f__Peptostreptococcaceae; g__Filifactor; s__ | 0 | 0 | 4 | 9 | 2 | denovo25139 |
| k__Bacteria; p__Firmicutes; c__Clostridia; o__Clostridiales; f__Peptostreptococcaceae; g__Peptostreptococcus; s__ | 0 | 0 | 11 | 169 | 88 | denovo21013 |
| k__Bacteria; p__Firmicutes; c__Clostridia; o__Clostridiales; f__Ruminococcaceae | 0 | 9 | 30 | 203 | 43 | denovo31993 |
| k__Bacteria; p__Firmicutes; c__Clostridia; o__Clostridiales; f__Ruminococcaceae; g__; s__ | 0 | 0 | 13 | 231 | 54 | denovo19218 |
| k__Bacteria; p__Firmicutes; c__Clostridia; o__Clostridiales; f__Ruminococcaceae; g__Butyricicoccus; s__pullicaecorum | 0 | 0 | 4 | 29 | 1 | denovo22808 |
| k__Bacteria; p__Firmicutes; c__Clostridia; o__Clostridiales; f__Ruminococcaceae; g__Clostridium; s__methylpentosum | 0 | 0 | 5 | 20 | 0 | denovo7141 |
| k__Bacteria; p__Firmicutes; c__Clostridia; o__Clostridiales; f__Ruminococcaceae; g__Faecalibacterium; s__prausnitzii | 9 | 1 | 110 | 1060 | 179 | denovo13475 |
| k__Bacteria; p__Firmicutes; c__Clostridia; o__Clostridiales; f__Ruminococcaceae; g__Oscillospira; s__ | 1 | 2 | 195 | 1377 | 490 | denovo27241 |
| k__Bacteria; p__Firmicutes; c__Clostridia; o__Clostridiales; f__Ruminococcaceae; g__Ruminococcus | 0 | 0 | 8 | 62 | 21 | denovo11837 |
| k__Bacteria; p__Firmicutes; c__Clostridia; o__Clostridiales; f__Ruminococcaceae; g__Ruminococcus; s__ | 7 | 2 | 53 | 717 | 207 | denovo14623 |
| k__Bacteria; p__Firmicutes; c__Clostridia; o__Clostridiales; f__Ruminococcaceae; g__Ruminococcus; s__bromii | 0 | 2 | 1 | 137 | 21 | denovo26655 |
| k__Bacteria; p__Firmicutes; c__Clostridia; o__Clostridiales; f__Ruminococcaceae; g__Ruminococcus; s__callidus | 0 | 0 | 0 | 22 | 9 | denovo6768 |
| k__Bacteria; p__Firmicutes; c__Clostridia; o__Clostridiales; f__Veillonellaceae; g__; s__ | 0 | 0 | 0 | 14 | 0 | denovo30578 |
| k__Bacteria; p__Firmicutes; c__Clostridia; o__Clostridiales; f__Veillonellaceae; g__Acidaminococcus; s__ | 0 | 0 | 5 | 49 | 10 | denovo36175 |
| k__Bacteria; p__Firmicutes; c__Clostridia; o__Clostridiales; f__Veillonellaceae; g__Dialister; s__ | 1 | 0 | 2 | 74 | 19 | denovo35715 |
| k__Bacteria; p__Firmicutes; c__Clostridia; o__Clostridiales; f__Veillonellaceae; g__Megasphaera; s__ | 2 | 0 | 29 | 156 | 111 | denovo41488 |
| k__Bacteria; p__Firmicutes; c__Clostridia; o__Clostridiales; f__Veillonellaceae; g__Mitsuokella; s__ | 0 | 0 | 0 | 7 | 0 | denovo5659 |
| k__Bacteria; p__Firmicutes; c__Clostridia; o__Clostridiales; f__Veillonellaceae; g__Phascolarctobacterium; s__ | 0 | 4 | 9 | 192 | 94 | denovo21995 |
| k__Bacteria; p__Firmicutes; c__Clostridia; o__Clostridiales; f__Veillonellaceae; g__Selenomonas; s__ | 9 | 0 | 44 | 749 | 472 | denovo14969 |
| k__Bacteria; p__Firmicutes; c__Clostridia; o__Clostridiales; f__Veillonellaceae; g__Selenomonas; s__noxia | 0 | 0 | 0 | 5 | 2 | denovo14944 |
| k__Bacteria; p__Firmicutes; c__Clostridia; o__Clostridiales; f__Veillonellaceae; g__Veillonella; s__ | 0 | 0 | 0 | 15 | 11 | denovo517 |
| k__Bacteria; p__Firmicutes; c__Clostridia; o__Clostridiales; f__Veillonellaceae; g__Veillonella; s__dispar | 10 | 1 | 128 | 1334 | 3438 | denovo35106 |
| k__Bacteria; p__Firmicutes; c__Clostridia; o__Clostridiales; f__Veillonellaceae; g__Veillonella; s__parvula | 0 | 0 | 0 | 4 | 13 | denovo9009 |
| k__Bacteria; p__Firmicutes; c__Erysipelotrichi; o__Erysipelotrichales; f__Erysipelotrichaceae | 0 | 6 | 17 | 269 | 124 | denovo4497 |
| k__Bacteria; p__Firmicutes; c__Erysipelotrichi; o__Erysipelotrichales; f__Erysipelotrichaceae; g__; s__ | 0 | 0 | 0 | 21 | 4 | denovo15840 |
| k__Bacteria; p__Firmicutes; c__Erysipelotrichi; o__Erysipelotrichales; f__Erysipelotrichaceae; g__[Eubacterium]; s__biforme | 0 | 0 | 1 | 58 | 2 | denovo31188 |
| k__Bacteria; p__Firmicutes; c__Erysipelotrichi; o__Erysipelotrichales; f__Erysipelotrichaceae; g__[Eubacterium]; s__dolichum | 0 | 0 | 4 | 39 | 22 | denovo5401 |
| k__Bacteria; p__Firmicutes; c__Erysipelotrichi; o__Erysipelotrichales; f__Erysipelotrichaceae; g__Allobaculum; s__ | 1 | 2 | 154 | 1020 | 532 | denovo20555 |
| k__Bacteria; p__Firmicutes; c__Erysipelotrichi; o__Erysipelotrichales; f__Erysipelotrichaceae; g__Bulleidia; s__moorei | 3 | 2 | 6 | 421 | 197 | denovo29578 |
| k__Bacteria; p__Firmicutes; c__Erysipelotrichi; o__Erysipelotrichales; f__Erysipelotrichaceae; g__Catenibacterium; s__ | 0 | 0 | 12 | 189 | 0 | denovo4911 |
| k__Bacteria; p__Firmicutes; c__Erysipelotrichi; o__Erysipelotrichales; f__Erysipelotrichaceae; g__Clostridium; s__saccharogumia | 2 | 1 | 13 | 44 | 21 | denovo16835 |
| k__Bacteria; p__Firmicutes; c__Erysipelotrichi; o__Erysipelotrichales; f__Erysipelotrichaceae; g__Coprobacillus; s__ | 0 | 0 | 11 | 7 | 6 | denovo6740 |
| k__Bacteria; p__Firmicutes; c__Erysipelotrichi; o__Erysipelotrichales; f__Erysipelotrichaceae; g__p-75-a5; s__ | 0 | 0 | 1 | 7 | 0 | denovo30924 |
| k__Bacteria; p__Firmicutes; c__Erysipelotrichi; o__Erysipelotrichales; f__Erysipelotrichaceae; g__Sharpea; s__ | 0 | 0 | 0 | 11 | 5 | denovo35335 |
| k__Bacteria; p__Fusobacteria; c__Fusobacteriia; o__Fusobacteriales; f__; g__; s__ | 0 | 3 | 1 | 0 | 2 | denovo16431 |
| k__Bacteria; p__Fusobacteria; c__Fusobacteriia; o__Fusobacteriales; f__Fusobacteriaceae | 0 | 4 | 30 | 253 | 100 | denovo7210 |
| k__Bacteria; p__Fusobacteria; c__Fusobacteriia; o__Fusobacteriales; f__Fusobacteriaceae; g__; s__ | 0 | 0 | 18 | 187 | 108 | denovo32643 |
| k__Bacteria; p__Fusobacteria; c__Fusobacteriia; o__Fusobacteriales; f__Fusobacteriaceae; g__Fusobacterium; s__ | 45 | 5 | 812 | 14956 | 8006 | denovo22147 |
| k__Bacteria; p__Fusobacteria; c__Fusobacteriia; o__Fusobacteriales; f__Fusobacteriaceae; g__Propionigenium; s__ | 195 | 22180 | 8558 | 1608 | 186 | denovo25306 |
| k__Bacteria; p__Fusobacteria; c__Fusobacteriia; o__Fusobacteriales; f__Fusobacteriaceae; g__Psychrilyobacter; s__ | 9 | 1 | 0 | 0 | 8 | denovo35103 |
| k__Bacteria; p__Fusobacteria; c__Fusobacteriia; o__Fusobacteriales; f__Leptotrichiaceae | 0 | 0 | 4 | 8 | 0 | denovo29632 |
| k__Bacteria; p__Fusobacteria; c__Fusobacteriia; o__Fusobacteriales; f__Leptotrichiaceae; g__; s__ | 0 | 0 | 0 | 9 | 5 | denovo3774 |
| k__Bacteria; p__Fusobacteria; c__Fusobacteriia; o__Fusobacteriales; f__Leptotrichiaceae; g__Leptotrichia; s__ | 1 | 0 | 85 | 1463 | 821 | denovo35603 |
| k__Bacteria; p__Fusobacteria; c__Fusobacteriia; o__Fusobacteriales; f__Leptotrichiaceae; g__Sneathia; s__ | 0 | 1 | 10 | 371 | 193 | denovo40698 |
| k__Bacteria; p__GN02 | 6 | 0 | 0 | 0 | 0 | denovo25723 |
| k__Bacteria; p__GN02; c__; o__; f__; g__; s__ | 170 | 0 | 0 | 0 | 3 | denovo43104 |
| k__Bacteria; p__GN02; c__BD1-5; o__; f__; g__; s__ | 585 | 1 | 105 | 1 | 112 | denovo33518 |
| k__Bacteria; p__GN02; c__IIB17; o__; f__; g__; s__ | 21 | 0 | 0 | 0 | 0 | denovo36876 |
| k__Bacteria; p__Lentisphaerae; c__[Lentisphaeria] | 1 | 2 | 3 | 10 | 2 | denovo3978 |
| k__Bacteria; p__Lentisphaerae; c__[Lentisphaeria]; o__Lentisphaerales | 29 | 0 | 0 | 0 | 0 | denovo7995 |
| k__Bacteria; p__Lentisphaerae; c__[Lentisphaeria]; o__Lentisphaerales; f__; g__; s__ | 0 | 74 | 212 | 23 | 0 | denovo6864 |
| k__Bacteria; p__Lentisphaerae; c__[Lentisphaeria]; o__Lentisphaerales; f__Lentisphaeraceae; g__; s__ | 18 | 0 | 0 | 0 | 0 | denovo43644 |
| k__Bacteria; p__Lentisphaerae; c__[Lentisphaeria]; o__Lentisphaerales; f__Lentisphaeraceae; g__Lentisphaera; s__ | 1 | 0 | 0 | 0 | 0 | denovo22796 |
| k__Bacteria; p__Lentisphaerae; c__[Lentisphaeria]; o__Victivallales; f__Victivallaceae; g__; s__ | 10 | 451 | 215 | 92 | 21 | denovo10691 |
| k__Bacteria; p__OD1 | 4 | 0 | 0 | 0 | 0 | denovo22319 |
| k__Bacteria; p__OD1; c__; o__; f__; g__; s__ | 22 | 0 | 0 | 0 | 0 | denovo20696 |
| k__Bacteria; p__OD1; c__SM2F11; o__; f__; g__; s__ | 3 | 0 | 0 | 0 | 0 | denovo42173 |
| k__Bacteria; p__OD1; c__ZB2; o__; f__; g__; s__ | 77 | 0 | 0 | 0 | 1 | denovo18670 |
| k__Bacteria; p__Planctomycetes; c__OM190; o__agg27; f__; g__; s__ | 274 | 0 | 0 | 0 | 8 | denovo34325 |
| k__Bacteria; p__Planctomycetes; c__OM190; o__CL500-15; f__; g__; s__ | 50 | 0 | 0 | 0 | 0 | denovo38976 |
| k__Bacteria; p__Planctomycetes; c__Phycisphaerae | 0 | 49 | 8 | 3 | 0 | denovo23272 |
| k__Bacteria; p__Planctomycetes; c__Phycisphaerae; o__AKAU3564; f__; g__; s__ | 0 | 15 | 3 | 20 | 0 | denovo34375 |
| k__Bacteria; p__Planctomycetes; c__Phycisphaerae; o__Phycisphaerales; f__; g__; s__ | 1 | 0 | 0 | 0 | 2 | denovo4451 |
| k__Bacteria; p__Planctomycetes; c__Phycisphaerae; o__Phycisphaerales; f__Phycisphaeraceae; g__; s__ | 15 | 0 | 5 | 11 | 3 | denovo7256 |
| k__Bacteria; p__Planctomycetes; c__Planctomycetia; o__Gemmatales; f__Isosphaeraceae; g__; s__ | 0 | 0 | 0 | 0 | 9 | denovo44362 |
| k__Bacteria; p__Planctomycetes; c__Planctomycetia; o__Pirellulales; f__Pirellulaceae | 0 | 0 | 14 | 0 | 0 | denovo12547 |
| k__Bacteria; p__Planctomycetes; c__Planctomycetia; o__Pirellulales; f__Pirellulaceae; g__; s__ | 335 | 0 | 15 | 22 | 110 | denovo14011 |
| k__Bacteria; p__Planctomycetes; c__Planctomycetia; o__Pirellulales; f__Pirellulaceae; g__Planctomycete; s__LF1 | 83 | 0 | 19 | 0 | 21 | denovo612 |
| k__Bacteria; p__Planctomycetes; c__Planctomycetia; o__Pirellulales; f__Pirellulaceae; g__planctomycete; s__MS30D1 | 32 | 0 | 6 | 0 | 4 | denovo40593 |
| k__Bacteria; p__Planctomycetes; c__Planctomycetia; o__Planctomycetales; f__Planctomycetaceae; g__Planctomyces; s__ | 158 | 0 | 2 | 47 | 27 | denovo5355 |
| k__Bacteria; p__Planctomycetes; c__vadinHA49; o__PeHg47; f__; g__; s__ | 0 | 137 | 112 | 20 | 0 | denovo33402 |
| k__Bacteria; p__Proteobacteria | 613 | 121 | 1302 | 125 | 104 | denovo22781 |
| k__Bacteria; p__Proteobacteria; c__Alphaproteobacteria | 1196 | 146 | 71 | 3488 | 360 | denovo43707 |
| k__Bacteria; p__Proteobacteria; c__Alphaproteobacteria; o__; f__; g__; s__ | 114 | 0 | 0 | 14 | 86 | denovo21173 |
| k__Bacteria; p__Proteobacteria; c__Alphaproteobacteria; o__BD7-3; f__; g__; s__ | 2124 | 0 | 0 | 6 | 635 | denovo31990 |
| k__Bacteria; p__Proteobacteria; c__Alphaproteobacteria; o__Caulobacterales; f__Caulobacteraceae | 3 | 0 | 90 | 47 | 185 | denovo40647 |
| k__Bacteria; p__Proteobacteria; c__Alphaproteobacteria; o__Caulobacterales; f__Caulobacteraceae; g__; s__ | 0 | 0 | 29 | 24 | 83 | denovo20321 |
| k__Bacteria; p__Proteobacteria; c__Alphaproteobacteria; o__Caulobacterales; f__Caulobacteraceae; g__Brevundimonas; s__diminuta | 0 | 0 | 1 | 1 | 66 | denovo16402 |
| k__Bacteria; p__Proteobacteria; c__Alphaproteobacteria; o__Caulobacterales; f__Caulobacteraceae; g__Brevundimonas; s__poindexterae | 0 | 0 | 10 | 9 | 53 | denovo17370 |
| k__Bacteria; p__Proteobacteria; c__Alphaproteobacteria; o__Caulobacterales; f__Caulobacteraceae; g__Caulobacter; s__ | 0 | 0 | 0 | 1 | 192 | denovo25425 |
| k__Bacteria; p__Proteobacteria; c__Alphaproteobacteria; o__Caulobacterales; f__Caulobacteraceae; g__Caulobacter; s__henricii | 0 | 0 | 3 | 1 | 2 | denovo20822 |
| k__Bacteria; p__Proteobacteria; c__Alphaproteobacteria; o__Caulobacterales; f__Caulobacteraceae; g__Mycoplana; s__ | 6 | 0 | 181 | 145 | 699 | denovo15945 |
| k__Bacteria; p__Proteobacteria; c__Alphaproteobacteria; o__Caulobacterales; f__Caulobacteraceae; g__Phenylobacterium; s__ | 0 | 0 | 30 | 32 | 340 | denovo26750 |
| k__Bacteria; p__Proteobacteria; c__Alphaproteobacteria; o__Kiloniellales; f__; g__; s__ | 75 | 7 | 0 | 0 | 9 | denovo23905 |
| k__Bacteria; p__Proteobacteria; c__Alphaproteobacteria; o__Kiloniellales; f__Kiloniellaceae; g__; s__ | 2 | 0 | 0 | 0 | 1 | denovo8873 |
| k__Bacteria; p__Proteobacteria; c__Alphaproteobacteria; o__RF32; f__; g__; s__ | 0 | 0 | 2 | 74 | 5 | denovo22890 |
| k__Bacteria; p__Proteobacteria; c__Alphaproteobacteria; o__Rhizobiales | 11 | 0 | 5 | 0 | 43 | denovo4727 |
| k__Bacteria; p__Proteobacteria; c__Alphaproteobacteria; o__Rhizobiales; f__Aurantimonadaceae; g__; s__ | 1 | 0 | 32 | 22 | 102 | denovo37087 |
| k__Bacteria; p__Proteobacteria; c__Alphaproteobacteria; o__Rhizobiales; f__Beijerinckiaceae; g__; s__ | 0 | 0 | 9 | 15 | 28 | denovo9948 |
| k__Bacteria; p__Proteobacteria; c__Alphaproteobacteria; o__Rhizobiales; f__Bradyrhizobiaceae; g__Balneimonas; s__ | 0 | 0 | 5 | 0 | 0 | denovo30934 |
| k__Bacteria; p__Proteobacteria; c__Alphaproteobacteria; o__Rhizobiales; f__Bradyrhizobiaceae; g__Bosea; s__genosp. | 0 | 0 | 1 | 7 | 0 | denovo12548 |
| k__Bacteria; p__Proteobacteria; c__Alphaproteobacteria; o__Rhizobiales; f__Bradyrhizobiaceae; g__Bradyrhizobium; s__ | 0 | 0 | 132 | 214 | 144 | denovo29210 |
| k__Bacteria; p__Proteobacteria; c__Alphaproteobacteria; o__Rhizobiales; f__Hyphomicrobiaceae | 0 | 1 | 0 | 8 | 0 | denovo15595 |
| k__Bacteria; p__Proteobacteria; c__Alphaproteobacteria; o__Rhizobiales; f__Hyphomicrobiaceae; g__; s__ | 6 | 0 | 2 | 10 | 15 | denovo1273 |
| k__Bacteria; p__Proteobacteria; c__Alphaproteobacteria; o__Rhizobiales; f__Hyphomicrobiaceae; g__Devosia; s__ | 0 | 0 | 12 | 12 | 32 | denovo1131 |
| k__Bacteria; p__Proteobacteria; c__Alphaproteobacteria; o__Rhizobiales; f__Hyphomicrobiaceae; g__Pannonibacter; s__phragmitetus | 4 | 0 | 45 | 29 | 90 | denovo19880 |
| k__Bacteria; p__Proteobacteria; c__Alphaproteobacteria; o__Rhizobiales; f__Methylobacteriaceae; g__; s__ | 0 | 0 | 2 | 4 | 492 | denovo3938 |
| k__Bacteria; p__Proteobacteria; c__Alphaproteobacteria; o__Rhizobiales; f__Methylobacteriaceae; g__Methylobacterium; s__ | 4 | 0 | 106 | 182 | 197 | denovo24945 |
| k__Bacteria; p__Proteobacteria; c__Alphaproteobacteria; o__Rhizobiales; f__Methylobacteriaceae; g__Methylobacterium; s__adhaesivum | 0 | 0 | 0 | 11 | 16 | denovo36532 |
| k__Bacteria; p__Proteobacteria; c__Alphaproteobacteria; o__Rhizobiales; f__Methylobacteriaceae; g__Methylobacterium; s__organophilum | 1 | 0 | 5 | 2 | 14 | denovo4924 |
| k__Bacteria; p__Proteobacteria; c__Alphaproteobacteria; o__Rhizobiales; f__Methylocystaceae; g__Pleomorphomonas; s__ | 0 | 0 | 29 | 15 | 76 | denovo22548 |
| k__Bacteria; p__Proteobacteria; c__Alphaproteobacteria; o__Rhizobiales; f__Phyllobacteriaceae; g__; s__ | 97 | 8 | 46 | 3 | 36 | denovo23767 |
| k__Bacteria; p__Proteobacteria; c__Alphaproteobacteria; o__Rhizobiales; f__Phyllobacteriaceae; g__Phyllobacterium; s__ | 2 | 0 | 48 | 90 | 54 | denovo29309 |
| k__Bacteria; p__Proteobacteria; c__Alphaproteobacteria; o__Rhizobiales; f__Rhizobiaceae | 0 | 0 | 0 | 0 | 17 | denovo36335 |
| k__Bacteria; p__Proteobacteria; c__Alphaproteobacteria; o__Rhizobiales; f__Rhizobiaceae; g__Agrobacterium | 0 | 0 | 0 | 1 | 9 | denovo15228 |
| k__Bacteria; p__Proteobacteria; c__Alphaproteobacteria; o__Rhizobiales; f__Rhizobiaceae; g__Agrobacterium; s__ | 5 | 0 | 82 | 127 | 876 | denovo11523 |
| k__Bacteria; p__Proteobacteria; c__Alphaproteobacteria; o__Rhizobiales; f__Rhizobiaceae; g__Kaistia; s__ | 0 | 0 | 0 | 0 | 10 | denovo40323 |
| k__Bacteria; p__Proteobacteria; c__Alphaproteobacteria; o__Rhizobiales; f__Rhizobiaceae; g__Shinella; s__granuli | 0 | 0 | 0 | 1 | 4 | denovo39428 |
| k__Bacteria; p__Proteobacteria; c__Alphaproteobacteria; o__Rhizobiales; f__Xanthobacteraceae; g__; s__ | 0 | 0 | 0 | 0 | 12 | denovo28199 |
| k__Bacteria; p__Proteobacteria; c__Alphaproteobacteria; o__Rhizobiales; f__Xanthobacteraceae; g__Azorhizobium; s__ | 0 | 0 | 4 | 6 | 13 | denovo11000 |
| k__Bacteria; p__Proteobacteria; c__Alphaproteobacteria; o__Rhizobiales; f__Xanthobacteraceae; g__Xanthobacter; s__ | 0 | 0 | 2 | 1 | 2 | denovo32202 |
| k__Bacteria; p__Proteobacteria; c__Alphaproteobacteria; o__Rhodobacterales; f__Hyphomonadaceae | 1709 | 0 | 0 | 1 | 355 | denovo39005 |
| k__Bacteria; p__Proteobacteria; c__Alphaproteobacteria; o__Rhodobacterales; f__Hyphomonadaceae; g__; s__ | 1862 | 0 | 9 | 63 | 245 | denovo4760 |
| k__Bacteria; p__Proteobacteria; c__Alphaproteobacteria; o__Rhodobacterales; f__Hyphomonadaceae; g__Hellea; s__balneolensis | 1270 | 0 | 0 | 8 | 40 | denovo42948 |
| k__Bacteria; p__Proteobacteria; c__Alphaproteobacteria; o__Rhodobacterales; f__Hyphomonadaceae; g__Hyphomonas; s__ | 1 | 0 | 5 | 5 | 0 | denovo20767 |
| k__Bacteria; p__Proteobacteria; c__Alphaproteobacteria; o__Rhodobacterales; f__Hyphomonadaceae; g__Maricaulis; s__ | 0 | 0 | 6 | 29 | 5 | denovo6289 |
| k__Bacteria; p__Proteobacteria; c__Alphaproteobacteria; o__Rhodobacterales; f__Hyphomonadaceae; g__Robiginitomaculum; s__antarcticum | 488 | 0 | 0 | 0 | 44 | denovo25165 |
| k__Bacteria; p__Proteobacteria; c__Alphaproteobacteria; o__Rhodobacterales; f__Rhodobacteraceae | 2711 | 12 | 503 | 13 | 303 | denovo15668 |
| k__Bacteria; p__Proteobacteria; c__Alphaproteobacteria; o__Rhodobacterales; f__Rhodobacteraceae; g__Anaerospora; s__ | 18 | 0 | 7 | 0 | 4 | denovo30616 |
| k__Bacteria; p__Proteobacteria; c__Alphaproteobacteria; o__Rhodobacterales; f__Rhodobacteraceae; g__Antarctobacter; s__ | 17 | 0 | 2 | 0 | 2 | denovo397 |
| k__Bacteria; p__Proteobacteria; c__Alphaproteobacteria; o__Rhodobacterales; f__Rhodobacteraceae; g__Loktanella; s__ | 981 | 0 | 5 | 0 | 115 | denovo27166 |
| k__Bacteria; p__Proteobacteria; c__Alphaproteobacteria; o__Rhodobacterales; f__Rhodobacteraceae; g__Octadecabacter | 13 | 0 | 0 | 0 | 0 | denovo11862 |
| k__Bacteria; p__Proteobacteria; c__Alphaproteobacteria; o__Rhodobacterales; f__Rhodobacteraceae; g__Octadecabacter; s__ | 5484 | 9 | 52 | 10 | 697 | denovo34012 |
| k__Bacteria; p__Proteobacteria; c__Alphaproteobacteria; o__Rhodobacterales; f__Rhodobacteraceae; g__Octadecabacter; s__antarcticus | 418 | 2 | 0 | 4 | 15 | denovo6382 |
| k__Bacteria; p__Proteobacteria; c__Alphaproteobacteria; o__Rhodobacterales; f__Rhodobacteraceae; g__Paracoccus | 1 | 1 | 10 | 42 | 117 | denovo10101 |
| k__Bacteria; p__Proteobacteria; c__Alphaproteobacteria; o__Rhodobacterales; f__Rhodobacteraceae; g__Paracoccus; s__ | 2 | 0 | 14 | 5 | 84 | denovo42158 |
| k__Bacteria; p__Proteobacteria; c__Alphaproteobacteria; o__Rhodobacterales; f__Rhodobacteraceae; g__Paracoccus; s__kawasakiensis | 0 | 0 | 0 | 0 | 5 | denovo15649 |
| k__Bacteria; p__Proteobacteria; c__Alphaproteobacteria; o__Rhodobacterales; f__Rhodobacteraceae; g__Phaeobacter | 3 | 0 | 0 | 0 | 0 | denovo5016 |
| k__Bacteria; p__Proteobacteria; c__Alphaproteobacteria; o__Rhodobacterales; f__Rhodobacteraceae; g__Phaeobacter; s__ | 335 | 9 | 124 | 1 | 64 | denovo4446 |
| k__Bacteria; p__Proteobacteria; c__Alphaproteobacteria; o__Rhodobacterales; f__Rhodobacteraceae; g__Phaeobacter; s__gallaeciensis | 22 | 0 | 1 | 0 | 0 | denovo4798 |
| k__Bacteria; p__Proteobacteria; c__Alphaproteobacteria; o__Rhodobacterales; f__Rhodobacteraceae; g__Pseudoruegeria; s__ | 121 | 0 | 18 | 4 | 15 | denovo20532 |
| k__Bacteria; p__Proteobacteria; c__Alphaproteobacteria; o__Rhodobacterales; f__Rhodobacteraceae; g__Rhodobacter; s__ | 0 | 0 | 0 | 7 | 19 | denovo21943 |
| k__Bacteria; p__Proteobacteria; c__Alphaproteobacteria; o__Rhodobacterales; f__Rhodobacteraceae; g__Rhodovulum; s__ | 0 | 1 | 8 | 33 | 5 | denovo12861 |
| k__Bacteria; p__Proteobacteria; c__Alphaproteobacteria; o__Rhodobacterales; f__Rhodobacteraceae; g__Rubellimicrobium; s__ | 0 | 0 | 1 | 2 | 5 | denovo34974 |
| k__Bacteria; p__Proteobacteria; c__Alphaproteobacteria; o__Rhodobacterales; f__Rhodobacteraceae; g__Ruegeria; s__ | 12 | 0 | 0 | 0 | 2 | denovo12007 |
| k__Bacteria; p__Proteobacteria; c__Alphaproteobacteria; o__Rhodobacterales; f__Rhodobacteraceae; g__Sulfitobacter; s__japonica | 8 | 0 | 0 | 0 | 34 | denovo33500 |
| k__Bacteria; p__Proteobacteria; c__Alphaproteobacteria; o__Rhodospirillales; f__; g__; s__ | 0 | 10 | 6 | 0 | 0 | denovo23929 |
| k__Bacteria; p__Proteobacteria; c__Alphaproteobacteria; o__Rhodospirillales; f__Acetobacteraceae | 0 | 0 | 0 | 0 | 50 | denovo7194 |
| k__Bacteria; p__Proteobacteria; c__Alphaproteobacteria; o__Rhodospirillales; f__Acetobacteraceae; g__; s__ | 0 | 0 | 1 | 2 | 9 | denovo6133 |
| k__Bacteria; p__Proteobacteria; c__Alphaproteobacteria; o__Rhodospirillales; f__Acetobacteraceae; g__Acetobacter; s__ | 0 | 0 | 0 | 11 | 3 | denovo14322 |
| k__Bacteria; p__Proteobacteria; c__Alphaproteobacteria; o__Rhodospirillales; f__Acetobacteraceae; g__Roseomonas; s__mucosa | 0 | 0 | 0 | 0 | 88 | denovo19392 |
| k__Bacteria; p__Proteobacteria; c__Alphaproteobacteria; o__Rhodospirillales; f__Acetobacteraceae; g__Roseomonas; s__rosea | 0 | 0 | 3 | 1 | 85 | denovo18991 |
| k__Bacteria; p__Proteobacteria; c__Alphaproteobacteria; o__Rhodospirillales; f__Rhodospirillaceae | 0 | 5 | 0 | 7 | 0 | denovo28629 |
| k__Bacteria; p__Proteobacteria; c__Alphaproteobacteria; o__Rhodospirillales; f__Rhodospirillaceae; g__; s__ | 0 | 0 | 0 | 0 | 11 | denovo43004 |
| k__Bacteria; p__Proteobacteria; c__Alphaproteobacteria; o__Rhodospirillales; f__Rhodospirillaceae; g__Azospirillum | 0 | 0 | 1 | 2 | 20 | denovo370 |
| k__Bacteria; p__Proteobacteria; c__Alphaproteobacteria; o__Rhodospirillales; f__Rhodospirillaceae; g__Azospirillum; s__ | 0 | 0 | 47 | 25 | 7 | denovo25637 |
| k__Bacteria; p__Proteobacteria; c__Alphaproteobacteria; o__Rhodospirillales; f__Rhodospirillaceae; g__Novispirillum; s__ | 0 | 0 | 8 | 2 | 33 | denovo11199 |
| k__Bacteria; p__Proteobacteria; c__Alphaproteobacteria; o__Rhodospirillales; f__Rhodospirillaceae; g__Rhodospirillum; s__centenum | 0 | 0 | 125 | 119 | 80 | denovo22759 |
| k__Bacteria; p__Proteobacteria; c__Alphaproteobacteria; o__Rhodospirillales; f__Rhodospirillaceae; g__Skermanella; s__ | 0 | 0 | 5 | 9 | 0 | denovo16803 |
| k__Bacteria; p__Proteobacteria; c__Alphaproteobacteria; o__Rickettsiales | 0 | 2 | 9 | 0 | 0 | denovo599 |
| k__Bacteria; p__Proteobacteria; c__Alphaproteobacteria; o__Rickettsiales; f__; g__; s__ | 58 | 18 | 5 | 21 | 18 | denovo9050 |
| k__Bacteria; p__Proteobacteria; c__Alphaproteobacteria; o__Rickettsiales; f__mitochondria | 4491 | 39 | 1 | 0 | 50 | denovo20324 |
| k__Bacteria; p__Proteobacteria; c__Alphaproteobacteria; o__Rickettsiales; f__mitochondria; g__; s__ | 431 | 14 | 76 | 0 | 67 | denovo35514 |
| k__Bacteria; p__Proteobacteria; c__Alphaproteobacteria; o__Rickettsiales; f__mitochondria; g__Podophyllum; s__peltatum | 0 | 0 | 3 | 10 | 109 | denovo573 |
| k__Bacteria; p__Proteobacteria; c__Alphaproteobacteria; o__Rickettsiales; f__mitochondria; g__Vermamoeba; s__vermiformis | 0 | 0 | 0 | 0 | 2 | denovo161 |
| k__Bacteria; p__Proteobacteria; c__Alphaproteobacteria; o__Rickettsiales; f__Pelagibacteraceae; g__; s__ | 0 | 0 | 0 | 6 | 9 | denovo25912 |
| k__Bacteria; p__Proteobacteria; c__Alphaproteobacteria; o__Rickettsiales; f__Rickettsiaceae | 0 | 6 | 18 | 0 | 0 | denovo4263 |
| k__Bacteria; p__Proteobacteria; c__Alphaproteobacteria; o__Rickettsiales; f__Rickettsiaceae; g__; s__ | 91 | 6 | 33 | 2 | 35 | denovo5110 |
| k__Bacteria; p__Proteobacteria; c__Alphaproteobacteria; o__Rickettsiales; f__Rickettsiaceae; g__Rickettsia | 0 | 0 | 11 | 0 | 0 | denovo3101 |
| k__Bacteria; p__Proteobacteria; c__Alphaproteobacteria; o__Rickettsiales; f__Rickettsiaceae; g__Rickettsia; s__ | 0 | 0 | 29 | 136 | 23 | denovo44044 |
| k__Bacteria; p__Proteobacteria; c__Alphaproteobacteria; o__Rickettsiales; f__Rickettsiaceae; g__Wolbachia; s__ | 0 | 0 | 0 | 9 | 4 | denovo34846 |
| k__Bacteria; p__Proteobacteria; c__Alphaproteobacteria; o__Sphingomonadales | 0 | 0 | 0 | 6 | 17 | denovo20330 |
| k__Bacteria; p__Proteobacteria; c__Alphaproteobacteria; o__Sphingomonadales; f__; g__; s__ | 30 | 0 | 0 | 1 | 16 | denovo13538 |
| k__Bacteria; p__Proteobacteria; c__Alphaproteobacteria; o__Sphingomonadales; f__Erythrobacteraceae | 40 | 0 | 0 | 0 | 18 | denovo19333 |
| k__Bacteria; p__Proteobacteria; c__Alphaproteobacteria; o__Sphingomonadales; f__Erythrobacteraceae; g__; s__ | 265 | 0 | 69 | 115 | 137 | denovo19040 |
| k__Bacteria; p__Proteobacteria; c__Alphaproteobacteria; o__Sphingomonadales; f__Erythrobacteraceae; g__Altererythrobacter; s__luteolus | 32 | 0 | 0 | 0 | 18 | denovo7687 |
| k__Bacteria; p__Proteobacteria; c__Alphaproteobacteria; o__Sphingomonadales; f__Sphingomonadaceae | 0 | 0 | 0 | 1 | 5 | denovo23850 |
| k__Bacteria; p__Proteobacteria; c__Alphaproteobacteria; o__Sphingomonadales; f__Sphingomonadaceae; g__; s__ | 0 | 0 | 1 | 3 | 0 | denovo14974 |
| k__Bacteria; p__Proteobacteria; c__Alphaproteobacteria; o__Sphingomonadales; f__Sphingomonadaceae; g__Blastomonas; s__natatoria | 0 | 0 | 5 | 1 | 3 | denovo3215 |
| k__Bacteria; p__Proteobacteria; c__Alphaproteobacteria; o__Sphingomonadales; f__Sphingomonadaceae; g__Kaistobacter; s__ | 0 | 1 | 18 | 17 | 11 | denovo11487 |
| k__Bacteria; p__Proteobacteria; c__Alphaproteobacteria; o__Sphingomonadales; f__Sphingomonadaceae; g__Novosphingobium | 0 | 0 | 0 | 0 | 12 | denovo25566 |
| k__Bacteria; p__Proteobacteria; c__Alphaproteobacteria; o__Sphingomonadales; f__Sphingomonadaceae; g__Novosphingobium; s__ | 0 | 0 | 38 | 36 | 147 | denovo44634 |
| k__Bacteria; p__Proteobacteria; c__Alphaproteobacteria; o__Sphingomonadales; f__Sphingomonadaceae; g__Sphingobium; s__ | 0 | 0 | 13 | 1 | 779 | denovo34037 |
| k__Bacteria; p__Proteobacteria; c__Alphaproteobacteria; o__Sphingomonadales; f__Sphingomonadaceae; g__Sphingomonas | 5 | 0 | 26 | 28 | 173 | denovo21542 |
| k__Bacteria; p__Proteobacteria; c__Alphaproteobacteria; o__Sphingomonadales; f__Sphingomonadaceae; g__Sphingomonas; s__ | 0 | 0 | 6 | 15 | 40 | denovo15569 |
| k__Bacteria; p__Proteobacteria; c__Betaproteobacteria; o__; f__; g__; s__ | 0 | 0 | 0 | 0 | 16 | denovo10661 |
| k__Bacteria; p__Proteobacteria; c__Betaproteobacteria; o__Burkholderiales | 86 | 0 | 11 | 30 | 4 | denovo5155 |
| k__Bacteria; p__Proteobacteria; c__Betaproteobacteria; o__Burkholderiales; f__Alcaligenaceae; g__Achromobacter; s__ | 0 | 0 | 2 | 2 | 99 | denovo18760 |
| k__Bacteria; p__Proteobacteria; c__Betaproteobacteria; o__Burkholderiales; f__Alcaligenaceae; g__Pigmentiphaga; s__ | 0 | 0 | 0 | 0 | 5 | denovo1828 |
| k__Bacteria; p__Proteobacteria; c__Betaproteobacteria; o__Burkholderiales; f__Alcaligenaceae; g__Sutterella; s__ | 0 | 0 | 52 | 201 | 102 | denovo40758 |
| k__Bacteria; p__Proteobacteria; c__Betaproteobacteria; o__Burkholderiales; f__Burkholderiaceae; g__Burkholderia | 0 | 0 | 4 | 24 | 9 | denovo34376 |
| k__Bacteria; p__Proteobacteria; c__Betaproteobacteria; o__Burkholderiales; f__Burkholderiaceae; g__Burkholderia; s__ | 37 | 55 | 28 | 129 | 83 | denovo40182 |
| k__Bacteria; p__Proteobacteria; c__Betaproteobacteria; o__Burkholderiales; f__Burkholderiaceae; g__Salinispora; s__ | 0 | 0 | 0 | 0 | 96 | denovo15413 |
| k__Bacteria; p__Proteobacteria; c__Betaproteobacteria; o__Burkholderiales; f__Comamonadaceae | 3 | 0 | 210 | 304 | 1257 | denovo13115 |
| k__Bacteria; p__Proteobacteria; c__Betaproteobacteria; o__Burkholderiales; f__Comamonadaceae; g__; s__ | 0 | 0 | 1 | 8 | 13 | denovo40432 |
| k__Bacteria; p__Proteobacteria; c__Betaproteobacteria; o__Burkholderiales; f__Comamonadaceae; g__Acidovorax | 0 | 1 | 3 | 4 | 40 | denovo21174 |
| k__Bacteria; p__Proteobacteria; c__Betaproteobacteria; o__Burkholderiales; f__Comamonadaceae; g__Acidovorax; s__ | 0 | 0 | 0 | 0 | 10 | denovo13634 |
| k__Bacteria; p__Proteobacteria; c__Betaproteobacteria; o__Burkholderiales; f__Comamonadaceae; g__Aquabacterium; s__ | 0 | 0 | 10 | 13 | 204 | denovo14929 |
| k__Bacteria; p__Proteobacteria; c__Betaproteobacteria; o__Burkholderiales; f__Comamonadaceae; g__Comamonas; s__ | 0 | 0 | 0 | 0 | 18 | denovo8797 |
| k__Bacteria; p__Proteobacteria; c__Betaproteobacteria; o__Burkholderiales; f__Comamonadaceae; g__Delftia; s__ | 2 | 1 | 55 | 170 | 3336 | denovo31636 |
| k__Bacteria; p__Proteobacteria; c__Betaproteobacteria; o__Burkholderiales; f__Comamonadaceae; g__Hydrogenophaga; s__ | 1 | 0 | 105 | 91 | 137 | denovo32229 |
| k__Bacteria; p__Proteobacteria; c__Betaproteobacteria; o__Burkholderiales; f__Comamonadaceae; g__Limnobacter; s__ | 0 | 0 | 5 | 4 | 7 | denovo15836 |
| k__Bacteria; p__Proteobacteria; c__Betaproteobacteria; o__Burkholderiales; f__Comamonadaceae; g__Limnohabitans; s__ | 0 | 0 | 1 | 5 | 23 | denovo32283 |
| k__Bacteria; p__Proteobacteria; c__Betaproteobacteria; o__Burkholderiales; f__Comamonadaceae; g__Methylibium; s__ | 0 | 0 | 77 | 109 | 587 | denovo12070 |
| k__Bacteria; p__Proteobacteria; c__Betaproteobacteria; o__Burkholderiales; f__Comamonadaceae; g__Rhodoferax; s__ | 0 | 0 | 1 | 0 | 4 | denovo4381 |
| k__Bacteria; p__Proteobacteria; c__Betaproteobacteria; o__Burkholderiales; f__Comamonadaceae; g__Schlegelella; s__ | 0 | 0 | 0 | 4 | 12 | denovo27911 |
| k__Bacteria; p__Proteobacteria; c__Betaproteobacteria; o__Burkholderiales; f__Comamonadaceae; g__Tepidimonas; s__ | 0 | 0 | 1 | 2 | 2 | denovo31311 |
| k__Bacteria; p__Proteobacteria; c__Betaproteobacteria; o__Burkholderiales; f__Oxalobacteraceae | 9 | 0 | 290 | 292 | 248 | denovo14548 |
| k__Bacteria; p__Proteobacteria; c__Betaproteobacteria; o__Burkholderiales; f__Oxalobacteraceae; g__; s__ | 0 | 0 | 92 | 89 | 25 | denovo21512 |
| k__Bacteria; p__Proteobacteria; c__Betaproteobacteria; o__Burkholderiales; f__Oxalobacteraceae; g__Cupriavidus; s__ | 0 | 0 | 20 | 7 | 27 | denovo15554 |
| k__Bacteria; p__Proteobacteria; c__Betaproteobacteria; o__Burkholderiales; f__Oxalobacteraceae; g__Herbaspirillum; s__ | 0 | 0 | 7 | 105 | 226 | denovo5348 |
| k__Bacteria; p__Proteobacteria; c__Betaproteobacteria; o__Burkholderiales; f__Oxalobacteraceae; g__Janthinobacterium; s__ | 3 | 0 | 229 | 303 | 2186 | denovo17568 |
| k__Bacteria; p__Proteobacteria; c__Betaproteobacteria; o__Burkholderiales; f__Oxalobacteraceae; g__Massilia | 0 | 0 | 24 | 22 | 4 | denovo37536 |
| k__Bacteria; p__Proteobacteria; c__Betaproteobacteria; o__Burkholderiales; f__Oxalobacteraceae; g__Massilia; s__alkalitolerans | 0 | 0 | 2 | 0 | 11 | denovo22970 |
| k__Bacteria; p__Proteobacteria; c__Betaproteobacteria; o__Burkholderiales; f__Oxalobacteraceae; g__Massilia; s__haematophila | 0 | 0 | 5 | 7 | 0 | denovo30379 |
| k__Bacteria; p__Proteobacteria; c__Betaproteobacteria; o__Burkholderiales; f__Oxalobacteraceae; g__Paucimonas; s__lemoignei | 0 | 0 | 6 | 2 | 9 | denovo2649 |
| k__Bacteria; p__Proteobacteria; c__Betaproteobacteria; o__Burkholderiales; f__Oxalobacteraceae; g__Ralstonia; s__ | 1 | 0 | 21 | 41 | 30 | denovo33968 |
| k__Bacteria; p__Proteobacteria; c__Betaproteobacteria; o__Methylophilales; f__Methylophilaceae; g__; s__ | 2 | 0 | 0 | 0 | 80 | denovo34648 |
| k__Bacteria; p__Proteobacteria; c__Betaproteobacteria; o__Methylophilales; f__Methylophilaceae; g__Methylotenera; s__mobilis | 69 | 0 | 7 | 0 | 2 | denovo5759 |
| k__Bacteria; p__Proteobacteria; c__Betaproteobacteria; o__Neisseriales; f__Neisseriaceae | 0 | 0 | 2 | 56 | 28 | denovo31003 |
| k__Bacteria; p__Proteobacteria; c__Betaproteobacteria; o__Neisseriales; f__Neisseriaceae; g__Chitinimonas; s__taiwanensis | 0 | 0 | 5 | 2 | 17 | denovo40613 |
| k__Bacteria; p__Proteobacteria; c__Betaproteobacteria; o__Neisseriales; f__Neisseriaceae; g__Eikenella; s__ | 0 | 0 | 2 | 39 | 34 | denovo3917 |
| k__Bacteria; p__Proteobacteria; c__Betaproteobacteria; o__Neisseriales; f__Neisseriaceae; g__Kingella; s__ | 0 | 0 | 0 | 2 | 51 | denovo43658 |
| k__Bacteria; p__Proteobacteria; c__Betaproteobacteria; o__Neisseriales; f__Neisseriaceae; g__Neisseria | 0 | 0 | 6 | 78 | 40 | denovo2339 |
| k__Bacteria; p__Proteobacteria; c__Betaproteobacteria; o__Neisseriales; f__Neisseriaceae; g__Neisseria; s__bacilliformis | 0 | 0 | 0 | 59 | 28 | denovo19346 |
| k__Bacteria; p__Proteobacteria; c__Betaproteobacteria; o__Neisseriales; f__Neisseriaceae; g__Neisseria; s__subflava | 20 | 2 | 158 | 3247 | 1779 | denovo15590 |
| k__Bacteria; p__Proteobacteria; c__Betaproteobacteria; o__Neisseriales; f__Neisseriaceae; g__Vogesella; s__ | 0 | 0 | 89 | 114 | 111 | denovo11901 |
| k__Bacteria; p__Proteobacteria; c__Betaproteobacteria; o__Rhodocyclales; f__Rhodocyclaceae; g__Hydrogenophilus; s__ | 0 | 0 | 2 | 1 | 11 | denovo18359 |
| k__Bacteria; p__Proteobacteria; c__Deltaproteobacteria | 525 | 15 | 25 | 3 | 9 | denovo40819 |
| k__Bacteria; p__Proteobacteria; c__Deltaproteobacteria; o__; f__; g__; s__ | 63 | 0 | 0 | 0 | 1 | denovo4795 |
| k__Bacteria; p__Proteobacteria; c__Deltaproteobacteria; o__Bdellovibrionales; f__Bacteriovoracaceae | 58 | 0 | 0 | 0 | 1 | denovo31848 |
| k__Bacteria; p__Proteobacteria; c__Deltaproteobacteria; o__Bdellovibrionales; f__Bacteriovoracaceae; g__; s__ | 950 | 0 | 2 | 2 | 6 | denovo33747 |
| k__Bacteria; p__Proteobacteria; c__Deltaproteobacteria; o__Bdellovibrionales; f__Bacteriovoracaceae; g__Bacteriovorax; s__ | 385 | 27 | 10 | 0 | 24 | denovo34825 |
| k__Bacteria; p__Proteobacteria; c__Deltaproteobacteria; o__Bdellovibrionales; f__Bacteriovoracaceae; g__Peredibacter; s__starrii | 0 | 0 | 0 | 5 | 10 | denovo28707 |
| k__Bacteria; p__Proteobacteria; c__Deltaproteobacteria; o__Bdellovibrionales; f__Bdellovibrionaceae; g__Bdellovibrio; s__ | 925 | 0 | 1 | 0 | 16 | denovo29158 |
| k__Bacteria; p__Proteobacteria; c__Deltaproteobacteria; o__Desulfobacterales; f__Desulfobacteraceae | 0 | 136 | 183 | 6 | 0 | denovo15824 |
| k__Bacteria; p__Proteobacteria; c__Deltaproteobacteria; o__Desulfobacterales; f__Desulfobacteraceae; g__; s__ | 2 | 217 | 372 | 109 | 5 | denovo10876 |
| k__Bacteria; p__Proteobacteria; c__Deltaproteobacteria; o__Desulfobacterales; f__Desulfobacteraceae; g__Desulfofrigus; s__ | 0 | 10 | 36 | 3 | 0 | denovo29423 |
| k__Bacteria; p__Proteobacteria; c__Deltaproteobacteria; o__Desulfobacterales; f__Desulfobulbaceae | 0 | 22 | 2 | 0 | 0 | denovo24897 |
| k__Bacteria; p__Proteobacteria; c__Deltaproteobacteria; o__Desulfobacterales; f__Desulfobulbaceae; g__; s__ | 4 | 6 | 18 | 0 | 0 | denovo15365 |
| k__Bacteria; p__Proteobacteria; c__Deltaproteobacteria; o__Desulfobacterales; f__Desulfobulbaceae; g__Desulfobulbus | 0 | 15 | 1 | 0 | 0 | denovo31237 |
| k__Bacteria; p__Proteobacteria; c__Deltaproteobacteria; o__Desulfobacterales; f__Desulfobulbaceae; g__Desulfotalea; s__ | 57 | 6802 | 6818 | 1330 | 71 | denovo22786 |
| k__Bacteria; p__Proteobacteria; c__Deltaproteobacteria; o__Desulfovibrionales | 0 | 13 | 95 | 2 | 0 | denovo9770 |
| k__Bacteria; p__Proteobacteria; c__Deltaproteobacteria; o__Desulfovibrionales; f__Desulfomicrobiaceae; g__; s__ | 0 | 0 | 0 | 0 | 2 | denovo41205 |
| k__Bacteria; p__Proteobacteria; c__Deltaproteobacteria; o__Desulfovibrionales; f__Desulfovibrionaceae; g__; s__ | 0 | 0 | 3 | 23 | 1 | denovo6284 |
| k__Bacteria; p__Proteobacteria; c__Deltaproteobacteria; o__Desulfovibrionales; f__Desulfovibrionaceae; g__Bilophila; s__ | 1 | 0 | 24 | 111 | 12 | denovo31386 |
| k__Bacteria; p__Proteobacteria; c__Deltaproteobacteria; o__Desulfovibrionales; f__Desulfovibrionaceae; g__Desulfovibrio | 0 | 164 | 39 | 27 | 0 | denovo13956 |
| k__Bacteria; p__Proteobacteria; c__Deltaproteobacteria; o__Desulfovibrionales; f__Desulfovibrionaceae; g__Desulfovibrio; s__ | 0 | 195 | 139 | 48 | 10 | denovo11838 |
| k__Bacteria; p__Proteobacteria; c__Deltaproteobacteria; o__Desulfovibrionales; f__Desulfovibrionaceae; g__Desulfovibrio; s__C21_c20 | 2 | 0 | 3 | 65 | 3 | denovo26164 |
| k__Bacteria; p__Proteobacteria; c__Deltaproteobacteria; o__Desulfovibrionales; f__Desulfovibrionaceae; g__Desulfovibrio; s__D168 | 0 | 0 | 0 | 0 | 10 | denovo4348 |
| k__Bacteria; p__Proteobacteria; c__Deltaproteobacteria; o__GMD14H09; f__; g__; s__ | 184 | 0 | 0 | 0 | 0 | denovo38446 |
| k__Bacteria; p__Proteobacteria; c__Deltaproteobacteria; o__MIZ46; f__; g__; s__ | 4 | 0 | 0 | 0 | 0 | denovo15996 |
| k__Bacteria; p__Proteobacteria; c__Deltaproteobacteria; o__Myxococcales | 49 | 0 | 0 | 0 | 0 | denovo30078 |
| k__Bacteria; p__Proteobacteria; c__Deltaproteobacteria; o__Myxococcales; f__; g__; s__ | 402 | 0 | 0 | 8 | 0 | denovo23237 |
| k__Bacteria; p__Proteobacteria; c__Deltaproteobacteria; o__Myxococcales; f__0319-6G20; g__; s__ | 0 | 0 | 6 | 0 | 0 | denovo18584 |
| k__Bacteria; p__Proteobacteria; c__Deltaproteobacteria; o__Myxococcales; f__Haliangiaceae; g__; s__ | 54 | 0 | 0 | 0 | 2 | denovo20803 |
| k__Bacteria; p__Proteobacteria; c__Deltaproteobacteria; o__Myxococcales; f__Haliangiaceae; g__Haliangium; s__ | 5 | 0 | 0 | 0 | 0 | denovo15243 |
| k__Bacteria; p__Proteobacteria; c__Deltaproteobacteria; o__Myxococcales; f__Nannocystaceae; g__Plesiocystis; s__ | 36 | 0 | 0 | 0 | 1 | denovo11218 |
| k__Bacteria; p__Proteobacteria; c__Deltaproteobacteria; o__Myxococcales; f__OM27; g__; s__ | 304 | 0 | 0 | 0 | 6 | denovo29157 |
| k__Bacteria; p__Proteobacteria; c__Deltaproteobacteria; o__PB19; f__; g__; s__ | 35 | 0 | 0 | 0 | 0 | denovo435 |
| k__Bacteria; p__Proteobacteria; c__Deltaproteobacteria; o__Spirobacillales; f__; g__; s__ | 165 | 0 | 0 | 0 | 3 | denovo23897 |
| k__Bacteria; p__Proteobacteria; c__Deltaproteobacteria; o__Syntrophobacterales; f__Syntrophobacteraceae; g__; s__ | 0 | 0 | 0 | 16 | 4 | denovo6394 |
| k__Bacteria; p__Proteobacteria; c__Epsilonproteobacteria; o__Campylobacterales | 10 | 0 | 302 | 4 | 2 | denovo36276 |
| k__Bacteria; p__Proteobacteria; c__Epsilonproteobacteria; o__Campylobacterales; f__Campylobacteraceae | 0 | 0 | 3306 | 9 | 2 | denovo22547 |
| k__Bacteria; p__Proteobacteria; c__Epsilonproteobacteria; o__Campylobacterales; f__Campylobacteraceae; g__; s__ | 0 | 0 | 48 | 0 | 0 | denovo7638 |
| k__Bacteria; p__Proteobacteria; c__Epsilonproteobacteria; o__Campylobacterales; f__Campylobacteraceae; g__Arcobacter | 0 | 13 | 126 | 0 | 0 | denovo9202 |
| k__Bacteria; p__Proteobacteria; c__Epsilonproteobacteria; o__Campylobacterales; f__Campylobacteraceae; g__Arcobacter; s__ | 15 | 125 | 30540 | 244 | 20 | denovo6297 |
| k__Bacteria; p__Proteobacteria; c__Epsilonproteobacteria; o__Campylobacterales; f__Campylobacteraceae; g__Campylobacter; s__ | 6 | 1 | 49 | 953 | 752 | denovo6348 |
| k__Bacteria; p__Proteobacteria; c__Epsilonproteobacteria; o__Campylobacterales; f__Campylobacteraceae; g__Campylobacter; s__rectus | 0 | 0 | 0 | 2 | 7 | denovo10244 |
| k__Bacteria; p__Proteobacteria; c__Epsilonproteobacteria; o__Campylobacterales; f__Campylobacteraceae; g__Campylobacter; s__ureolyticus | 0 | 0 | 1 | 2 | 2 | denovo44081 |
| k__Bacteria; p__Proteobacteria; c__Epsilonproteobacteria; o__Campylobacterales; f__Helicobacteraceae | 0 | 0 | 1 | 23 | 0 | denovo9780 |
| k__Bacteria; p__Proteobacteria; c__Epsilonproteobacteria; o__Campylobacterales; f__Helicobacteraceae; g__Flexispira; s__ | 0 | 0 | 4 | 15 | 4 | denovo5538 |
| k__Bacteria; p__Proteobacteria; c__Epsilonproteobacteria; o__Campylobacterales; f__Helicobacteraceae; g__Helicobacter; s__ | 0 | 0 | 22 | 117 | 21 | denovo5043 |
| k__Bacteria; p__Proteobacteria; c__Epsilonproteobacteria; o__Campylobacterales; f__Helicobacteraceae; g__Helicobacter; s__apodemus | 2 | 0 | 16 | 182 | 49 | denovo42878 |
| k__Bacteria; p__Proteobacteria; c__Epsilonproteobacteria; o__Campylobacterales; f__Helicobacteraceae; g__Helicobacter; s__pylori | 0 | 0 | 0 | 0 | 6 | denovo35267 |
| k__Bacteria; p__Proteobacteria; c__Epsilonproteobacteria; o__Campylobacterales; f__Helicobacteraceae; g__Sulfurimonas | 0 | 0 | 12 | 99 | 0 | denovo17753 |
| k__Bacteria; p__Proteobacteria; c__Epsilonproteobacteria; o__Campylobacterales; f__Helicobacteraceae; g__Sulfurimonas; s__ | 1 | 36 | 18450 | 5103 | 3 | denovo22540 |
| k__Bacteria; p__Proteobacteria; c__Epsilonproteobacteria; o__Campylobacterales; f__Helicobacteraceae; g__Sulfurimonas; s__autotrophica | 0 | 3 | 14 | 0 | 0 | denovo28504 |
| k__Bacteria; p__Proteobacteria; c__Gammaproteobacteria | 8684 | 39 | 7552 | 9275 | 141 | denovo39124 |
| k__Bacteria; p__Proteobacteria; c__Gammaproteobacteria; o__; f__; g__; s__ | 2836 | 0 | 0 | 6 | 1 | denovo5350 |
| k__Bacteria; p__Proteobacteria; c__Gammaproteobacteria; o__[Marinicellales]; f__[Marinicellaceae]; g__; s__ | 5 | 0 | 0 | 0 | 1 | denovo31550 |
| k__Bacteria; p__Proteobacteria; c__Gammaproteobacteria; o__[Marinicellales]; f__[Marinicellaceae]; g__Marinicella; s__ | 86 | 0 | 0 | 0 | 29 | denovo22384 |
| k__Bacteria; p__Proteobacteria; c__Gammaproteobacteria; o__34P16; f__; g__; s__ | 0 | 0 | 0 | 4 | 2 | denovo32196 |
| k__Bacteria; p__Proteobacteria; c__Gammaproteobacteria; o__Alteromonadales | 29 | 245 | 13 | 35 | 22 | denovo16300 |
| k__Bacteria; p__Proteobacteria; c__Gammaproteobacteria; o__Alteromonadales; f__; g__; s__ | 18 | 3 | 6 | 0 | 11 | denovo13460 |
| k__Bacteria; p__Proteobacteria; c__Gammaproteobacteria; o__Alteromonadales; f__[Chromatiaceae]; g__; s__ | 0 | 0 | 0 | 4 | 20 | denovo3818 |
| k__Bacteria; p__Proteobacteria; c__Gammaproteobacteria; o__Alteromonadales; f__[Chromatiaceae]; g__Alishewanella; s__ | 0 | 0 | 1 | 0 | 4 | denovo28803 |
| k__Bacteria; p__Proteobacteria; c__Gammaproteobacteria; o__Alteromonadales; f__[Chromatiaceae]; g__Rheinheimera; s__ | 0 | 0 | 69 | 104 | 1372 | denovo19065 |
| k__Bacteria; p__Proteobacteria; c__Gammaproteobacteria; o__Alteromonadales; f__Alteromonadaceae | 33 | 7 | 0 | 0 | 5 | denovo3775 |
| k__Bacteria; p__Proteobacteria; c__Gammaproteobacteria; o__Alteromonadales; f__Alteromonadaceae; g__; s__ | 498 | 0 | 0 | 1 | 36 | denovo17585 |
| k__Bacteria; p__Proteobacteria; c__Gammaproteobacteria; o__Alteromonadales; f__Alteromonadaceae; g__Agarivorans; s__ | 4 | 62 | 0 | 4 | 1 | denovo27380 |
| k__Bacteria; p__Proteobacteria; c__Gammaproteobacteria; o__Alteromonadales; f__Alteromonadaceae; g__Candidatus Endobugula; s__ | 646 | 0 | 3 | 0 | 70 | denovo16983 |
| k__Bacteria; p__Proteobacteria; c__Gammaproteobacteria; o__Alteromonadales; f__Alteromonadaceae; g__gas; s__vacuolate | 100 | 0 | 0 | 0 | 27 | denovo3123 |
| k__Bacteria; p__Proteobacteria; c__Gammaproteobacteria; o__Alteromonadales; f__Alteromonadaceae; g__Glaciecola | 4 | 0 | 0 | 0 | 2 | denovo13131 |
| k__Bacteria; p__Proteobacteria; c__Gammaproteobacteria; o__Alteromonadales; f__Alteromonadaceae; g__Glaciecola; s__ | 158 | 0 | 0 | 5 | 86 | denovo35839 |
| k__Bacteria; p__Proteobacteria; c__Gammaproteobacteria; o__Alteromonadales; f__Alteromonadaceae; g__Glaciecola; s__punicea | 152 | 0 | 0 | 1 | 60 | denovo17040 |
| k__Bacteria; p__Proteobacteria; c__Gammaproteobacteria; o__Alteromonadales; f__Alteromonadaceae; g__HTCC2207; s__ | 3 | 0 | 0 | 0 | 57 | denovo31365 |
| k__Bacteria; p__Proteobacteria; c__Gammaproteobacteria; o__Alteromonadales; f__Alteromonadaceae; g__Marinobacter; s__ | 0 | 0 | 0 | 6 | 0 | denovo21951 |
| k__Bacteria; p__Proteobacteria; c__Gammaproteobacteria; o__Alteromonadales; f__Alteromonadaceae; g__unclassified; s__Pseudomonadales | 77 | 0 | 0 | 0 | 1 | denovo8944 |
| k__Bacteria; p__Proteobacteria; c__Gammaproteobacteria; o__Alteromonadales; f__Alteromonadaceae; g__ZD0117; s__ | 0 | 0 | 0 | 0 | 4 | denovo35292 |
| k__Bacteria; p__Proteobacteria; c__Gammaproteobacteria; o__Alteromonadales; f__Colwelliaceae | 6 | 0 | 0 | 0 | 2 | denovo42651 |
| k__Bacteria; p__Proteobacteria; c__Gammaproteobacteria; o__Alteromonadales; f__Colwelliaceae; g__; s__ | 34 | 159 | 24 | 10 | 77 | denovo971 |
| k__Bacteria; p__Proteobacteria; c__Gammaproteobacteria; o__Alteromonadales; f__Colwelliaceae; g__Colwellia; s__ | 0 | 8 | 0 | 1 | 1 | denovo44576 |
| k__Bacteria; p__Proteobacteria; c__Gammaproteobacteria; o__Alteromonadales; f__Colwelliaceae; g__Thalassomonas | 0 | 64 | 12 | 1 | 0 | denovo12815 |
| k__Bacteria; p__Proteobacteria; c__Gammaproteobacteria; o__Alteromonadales; f__Colwelliaceae; g__Thalassomonas; s__ | 19 | 29 | 0 | 9 | 106 | denovo12088 |
| k__Bacteria; p__Proteobacteria; c__Gammaproteobacteria; o__Alteromonadales; f__Colwelliaceae; g__Thalassomonas; s__sediminis | 2 | 1629 | 226 | 64 | 9 | denovo2290 |
| k__Bacteria; p__Proteobacteria; c__Gammaproteobacteria; o__Alteromonadales; f__HTCC2188; g__; s__ | 762 | 0 | 0 | 0 | 4 | denovo30809 |
| k__Bacteria; p__Proteobacteria; c__Gammaproteobacteria; o__Alteromonadales; f__HTCC2188; g__HTCC; s__ | 22 | 0 | 0 | 7 | 27 | denovo40843 |
| k__Bacteria; p__Proteobacteria; c__Gammaproteobacteria; o__Alteromonadales; f__Moritellaceae; g__Moritella; s__ | 9 | 217 | 5 | 22 | 3 | denovo36704 |
| k__Bacteria; p__Proteobacteria; c__Gammaproteobacteria; o__Alteromonadales; f__OM60; g__; s__ | 57 | 10 | 54 | 8 | 139 | denovo18928 |
| k__Bacteria; p__Proteobacteria; c__Gammaproteobacteria; o__Alteromonadales; f__OM60; g__Congregibacter; s__ | 6 | 0 | 8 | 1 | 1 | denovo13327 |
| k__Bacteria; p__Proteobacteria; c__Gammaproteobacteria; o__Alteromonadales; f__Psychromonadaceae | 0 | 608 | 15 | 0 | 0 | denovo14360 |
| k__Bacteria; p__Proteobacteria; c__Gammaproteobacteria; o__Alteromonadales; f__Psychromonadaceae; g__Psychromonas; s__ | 519 | 60778 | 10176 | 3259 | 623 | denovo22141 |
| k__Bacteria; p__Proteobacteria; c__Gammaproteobacteria; o__Alteromonadales; f__Shewanellaceae; g__Shewanella | 0 | 5 | 0 | 0 | 0 | denovo34272 |
| k__Bacteria; p__Proteobacteria; c__Gammaproteobacteria; o__Alteromonadales; f__Shewanellaceae; g__Shewanella; s__ | 5 | 604 | 183 | 28 | 9 | denovo4762 |
| k__Bacteria; p__Proteobacteria; c__Gammaproteobacteria; o__Cardiobacteriales; f__Cardiobacteriaceae; g__Cardiobacterium; s__ | 0 | 0 | 1 | 9 | 7 | denovo2024 |
| k__Bacteria; p__Proteobacteria; c__Gammaproteobacteria; o__Chromatiales | 1 | 0 | 0 | 0 | 9 | denovo27933 |
| k__Bacteria; p__Proteobacteria; c__Gammaproteobacteria; o__Chromatiales; f__; g__; s__ | 683 | 0 | 1 | 28 | 98 | denovo20316 |
| k__Bacteria; p__Proteobacteria; c__Gammaproteobacteria; o__Enterobacteriales; f__Enterobacteriaceae | 101 | 5 | 147 | 935 | 710 | denovo21757 |
| k__Bacteria; p__Proteobacteria; c__Gammaproteobacteria; o__Enterobacteriales; f__Enterobacteriaceae; g__Escherichia; s__coli | 3 | 3 | 59 | 478 | 219 | denovo15225 |
| k__Bacteria; p__Proteobacteria; c__Gammaproteobacteria; o__Enterobacteriales; f__Enterobacteriaceae; g__Morganella; s__ | 0 | 0 | 5 | 12 | 5 | denovo38877 |
| k__Bacteria; p__Proteobacteria; c__Gammaproteobacteria; o__Enterobacteriales; f__Enterobacteriaceae; g__Proteus; s__ | 0 | 0 | 1 | 32 | 0 | denovo15958 |
| k__Bacteria; p__Proteobacteria; c__Gammaproteobacteria; o__HTCC2188; f__HTCC2089; g__; s__ | 1393 | 0 | 0 | 0 | 16 | denovo39472 |
| k__Bacteria; p__Proteobacteria; c__Gammaproteobacteria; o__Legionellales; f__Coxiellaceae; g__; s__ | 0 | 128 | 613 | 1 | 7 | denovo8484 |
| k__Bacteria; p__Proteobacteria; c__Gammaproteobacteria; o__Legionellales; f__Francisellaceae | 9 | 0 | 46 | 2 | 0 | denovo149 |
| k__Bacteria; p__Proteobacteria; c__Gammaproteobacteria; o__Legionellales; f__Legionellaceae; g__; s__ | 0 | 0 | 1 | 0 | 3 | denovo31910 |
| k__Bacteria; p__Proteobacteria; c__Gammaproteobacteria; o__Oceanospirillales | 0 | 0 | 0 | 1170 | 0 | denovo34981 |
| k__Bacteria; p__Proteobacteria; c__Gammaproteobacteria; o__Oceanospirillales; f__; g__; s__ | 46 | 0 | 0 | 0 | 5 | denovo40858 |
| k__Bacteria; p__Proteobacteria; c__Gammaproteobacteria; o__Oceanospirillales; f__Endozoicimonaceae; g__; s__ | 0 | 0 | 0 | 29 | 0 | denovo42564 |
| k__Bacteria; p__Proteobacteria; c__Gammaproteobacteria; o__Oceanospirillales; f__Halomonadaceae; g__Candidatus Portiera; s__ | 24 | 0 | 6 | 16 | 340 | denovo6514 |
| k__Bacteria; p__Proteobacteria; c__Gammaproteobacteria; o__Oceanospirillales; f__Halomonadaceae; g__Cobetia; s__ | 0 | 0 | 0 | 2 | 4 | denovo4106 |
| k__Bacteria; p__Proteobacteria; c__Gammaproteobacteria; o__Oceanospirillales; f__Oceanospirillaceae; g__; s__ | 1 | 0 | 0 | 0 | 32 | denovo4055 |
| k__Bacteria; p__Proteobacteria; c__Gammaproteobacteria; o__Oceanospirillales; f__Oceanospirillaceae; g__Amphritea; s__ | 6 | 0 | 0 | 1 | 8 | denovo37225 |
| k__Bacteria; p__Proteobacteria; c__Gammaproteobacteria; o__Oceanospirillales; f__Oceanospirillaceae; g__Marinomonas; s__ | 14 | 0 | 0 | 0 | 16 | denovo18947 |
| k__Bacteria; p__Proteobacteria; c__Gammaproteobacteria; o__Oceanospirillales; f__Oceanospirillaceae; g__Oleispira; s__ | 5 | 0 | 0 | 0 | 2 | denovo5603 |
| k__Bacteria; p__Proteobacteria; c__Gammaproteobacteria; o__Oceanospirillales; f__Oceanospirillaceae; g__Spongiispira; s__norvegica | 23 | 0 | 0 | 0 | 1 | denovo37274 |
| k__Bacteria; p__Proteobacteria; c__Gammaproteobacteria; o__Oceanospirillales; f__Oleiphilaceae; g__; s__ | 5 | 0 | 0 | 0 | 0 | denovo21717 |
| k__Bacteria; p__Proteobacteria; c__Gammaproteobacteria; o__Oceanospirillales; f__Saccharospirillaceae; g__Reinekea; s__ | 6 | 0 | 0 | 0 | 4 | denovo558 |
| k__Bacteria; p__Proteobacteria; c__Gammaproteobacteria; o__Oceanospirillales; f__SUP05; g__; s__ | 2 | 0 | 0 | 0 | 65 | denovo32828 |
| k__Bacteria; p__Proteobacteria; c__Gammaproteobacteria; o__Pasteurellales; f__Pasteurellaceae | 1 | 0 | 21 | 181 | 117 | denovo44469 |
| k__Bacteria; p__Proteobacteria; c__Gammaproteobacteria; o__Pasteurellales; f__Pasteurellaceae; g__Actinobacillus | 5 | 3 | 95 | 1548 | 727 | denovo30986 |
| k__Bacteria; p__Proteobacteria; c__Gammaproteobacteria; o__Pasteurellales; f__Pasteurellaceae; g__Actinobacillus; s__ | 0 | 0 | 1 | 4 | 2 | denovo6355 |
| k__Bacteria; p__Proteobacteria; c__Gammaproteobacteria; o__Pasteurellales; f__Pasteurellaceae; g__Actinobacillus; s__parahaemolyticus | 0 | 0 | 8 | 63 | 37 | denovo4075 |
| k__Bacteria; p__Proteobacteria; c__Gammaproteobacteria; o__Pasteurellales; f__Pasteurellaceae; g__Actinobacillus; s__porcinus | 0 | 0 | 3 | 88 | 76 | denovo29795 |
| k__Bacteria; p__Proteobacteria; c__Gammaproteobacteria; o__Pasteurellales; f__Pasteurellaceae; g__Aggregatibacter | 0 | 0 | 0 | 15 | 6 | denovo35192 |
| k__Bacteria; p__Proteobacteria; c__Gammaproteobacteria; o__Pasteurellales; f__Pasteurellaceae; g__Aggregatibacter; s__ | 5 | 0 | 54 | 811 | 477 | denovo41883 |
| k__Bacteria; p__Proteobacteria; c__Gammaproteobacteria; o__Pasteurellales; f__Pasteurellaceae; g__Aggregatibacter; s__pneumotropica | 0 | 0 | 1 | 6 | 2 | denovo38597 |
| k__Bacteria; p__Proteobacteria; c__Gammaproteobacteria; o__Pasteurellales; f__Pasteurellaceae; g__Aggregatibacter; s__segnis | 0 | 0 | 3 | 101 | 44 | denovo24079 |
| k__Bacteria; p__Proteobacteria; c__Gammaproteobacteria; o__Pasteurellales; f__Pasteurellaceae; g__Haemophilus; s__influenzae | 0 | 0 | 0 | 6 | 6 | denovo25601 |
| k__Bacteria; p__Proteobacteria; c__Gammaproteobacteria; o__Pasteurellales; f__Pasteurellaceae; g__Mannheimia; s__ | 0 | 0 | 5 | 81 | 11 | denovo30962 |
| k__Bacteria; p__Proteobacteria; c__Gammaproteobacteria; o__Pseudomonadales; f__Moraxellaceae; g__Acinetobacter | 0 | 0 | 9 | 8 | 14 | denovo5303 |
| k__Bacteria; p__Proteobacteria; c__Gammaproteobacteria; o__Pseudomonadales; f__Moraxellaceae; g__Acinetobacter; s__ | 7 | 8 | 798 | 2632 | 1714 | denovo13770 |
| k__Bacteria; p__Proteobacteria; c__Gammaproteobacteria; o__Pseudomonadales; f__Moraxellaceae; g__Acinetobacter; s__guillouiae | 0 | 0 | 2 | 3 | 1 | denovo32025 |
| k__Bacteria; p__Proteobacteria; c__Gammaproteobacteria; o__Pseudomonadales; f__Moraxellaceae; g__Acinetobacter; s__lwoffii | 2 | 0 | 12 | 39 | 36 | denovo39966 |
| k__Bacteria; p__Proteobacteria; c__Gammaproteobacteria; o__Pseudomonadales; f__Moraxellaceae; g__Acinetobacter; s__rhizosphaerae | 0 | 3 | 1 | 14 | 6 | denovo5284 |
| k__Bacteria; p__Proteobacteria; c__Gammaproteobacteria; o__Pseudomonadales; f__Moraxellaceae; g__Alkanindiges; s__ | 0 | 0 | 7 | 0 | 5 | denovo30281 |
| k__Bacteria; p__Proteobacteria; c__Gammaproteobacteria; o__Pseudomonadales; f__Moraxellaceae; g__Enhydrobacter; s__ | 3 | 0 | 13 | 19 | 81 | denovo20476 |
| k__Bacteria; p__Proteobacteria; c__Gammaproteobacteria; o__Pseudomonadales; f__Moraxellaceae; g__Psychrobacter | 0 | 0 | 5 | 3 | 3 | denovo28584 |
| k__Bacteria; p__Proteobacteria; c__Gammaproteobacteria; o__Pseudomonadales; f__Pseudomonadaceae | 2 | 0 | 4 | 7 | 352 | denovo35172 |
| k__Bacteria; p__Proteobacteria; c__Gammaproteobacteria; o__Pseudomonadales; f__Pseudomonadaceae; g__Pseudomonas | 2 | 0 | 52 | 190 | 2690 | denovo22195 |
| k__Bacteria; p__Proteobacteria; c__Gammaproteobacteria; o__Pseudomonadales; f__Pseudomonadaceae; g__Pseudomonas; s__ | 0 | 0 | 1 | 9 | 48 | denovo1199 |
| k__Bacteria; p__Proteobacteria; c__Gammaproteobacteria; o__Pseudomonadales; f__Pseudomonadaceae; g__Pseudomonas; s__alcaligenes | 1 | 0 | 3 | 30 | 424 | denovo17412 |
| k__Bacteria; p__Proteobacteria; c__Gammaproteobacteria; o__Pseudomonadales; f__Pseudomonadaceae; g__Pseudomonas; s__balearica | 0 | 0 | 1 | 6 | 16 | denovo18897 |
| k__Bacteria; p__Proteobacteria; c__Gammaproteobacteria; o__Pseudomonadales; f__Pseudomonadaceae; g__Pseudomonas; s__fragi | 0 | 0 | 3 | 1 | 1 | denovo29331 |
| k__Bacteria; p__Proteobacteria; c__Gammaproteobacteria; o__Pseudomonadales; f__Pseudomonadaceae; g__Pseudomonas; s__pseudoalcaligenes | 0 | 0 | 14 | 6 | 18 | denovo1706 |
| k__Bacteria; p__Proteobacteria; c__Gammaproteobacteria; o__Pseudomonadales; f__Pseudomonadaceae; g__Pseudomonas; s__stutzeri | 0 | 0 | 11 | 2 | 28 | denovo39633 |
| k__Bacteria; p__Proteobacteria; c__Gammaproteobacteria; o__Pseudomonadales; f__Pseudomonadaceae; g__Pseudomonas; s__veronii | 0 | 0 | 8 | 19 | 0 | denovo14745 |
| k__Bacteria; p__Proteobacteria; c__Gammaproteobacteria; o__Thiohalorhabdales | 610 | 1 | 0 | 0 | 0 | denovo21130 |
| k__Bacteria; p__Proteobacteria; c__Gammaproteobacteria; o__Thiohalorhabdales; f__; g__; s__ | 1984 | 15 | 12 | 16 | 84 | denovo1770 |
| k__Bacteria; p__Proteobacteria; c__Gammaproteobacteria; o__Thiohalorhabdales; f__Thiohalorhabdaceae; g__; s__ | 30 | 0 | 0 | 0 | 0 | denovo23648 |
| k__Bacteria; p__Proteobacteria; c__Gammaproteobacteria; o__Thiotrichales; f__Piscirickettsiaceae | 4 | 0 | 1 | 0 | 12 | denovo25547 |
| k__Bacteria; p__Proteobacteria; c__Gammaproteobacteria; o__Thiotrichales; f__Piscirickettsiaceae; g__; s__ | 78 | 3 | 0 | 0 | 9 | denovo3612 |
| k__Bacteria; p__Proteobacteria; c__Gammaproteobacteria; o__Thiotrichales; f__Thiotrichaceae | 112 | 0 | 0 | 0 | 6 | denovo2176 |
| k__Bacteria; p__Proteobacteria; c__Gammaproteobacteria; o__Thiotrichales; f__Thiotrichaceae; g__Cocleimonas; s__ | 1947 | 0 | 0 | 0 | 46 | denovo17542 |
| k__Bacteria; p__Proteobacteria; c__Gammaproteobacteria; o__Thiotrichales; f__Thiotrichaceae; g__Leucothrix; s__ | 4652 | 0 | 0 | 1 | 64 | denovo22782 |
| k__Bacteria; p__Proteobacteria; c__Gammaproteobacteria; o__Thiotrichales; f__Thiotrichaceae; g__Thiothrix; s__ | 15 | 0 | 0 | 2 | 6 | denovo6656 |
| k__Bacteria; p__Proteobacteria; c__Gammaproteobacteria; o__Vibrionales; f__Pseudoalteromonadaceae; g__Pseudoalteromonas; s__ | 3 | 0 | 1 | 8 | 32 | denovo34074 |
| k__Bacteria; p__Proteobacteria; c__Gammaproteobacteria; o__Vibrionales; f__Pseudoalteromonadaceae; g__Pseudoalteromonas; s__tunicata | 0 | 0 | 0 | 0 | 24 | denovo25478 |
| k__Bacteria; p__Proteobacteria; c__Gammaproteobacteria; o__Vibrionales; f__Vibrionaceae | 11 | 2477 | 539 | 10 | 17 | denovo4861 |
| k__Bacteria; p__Proteobacteria; c__Gammaproteobacteria; o__Vibrionales; f__Vibrionaceae; g__Aliivibrio; s__fischeri | 29 | 707 | 123 | 48 | 42 | denovo8457 |
| k__Bacteria; p__Proteobacteria; c__Gammaproteobacteria; o__Vibrionales; f__Vibrionaceae; g__Photobacterium | 0 | 0 | 1 | 11 | 4 | denovo19329 |
| k__Bacteria; p__Proteobacteria; c__Gammaproteobacteria; o__Vibrionales; f__Vibrionaceae; g__Photobacterium; s__ | 1 | 7 | 0 | 0 | 5 | denovo17589 |
| k__Bacteria; p__Proteobacteria; c__Gammaproteobacteria; o__Vibrionales; f__Vibrionaceae; g__Vibrio | 0 | 192 | 3 | 6 | 0 | denovo24129 |
| k__Bacteria; p__Proteobacteria; c__Gammaproteobacteria; o__Vibrionales; f__Vibrionaceae; g__Vibrio; s__ | 42 | 1881 | 246 | 282 | 30 | denovo16797 |
| k__Bacteria; p__Proteobacteria; c__Gammaproteobacteria; o__Xanthomonadales; f__Xanthomonadaceae; g__; s__ | 0 | 0 | 1 | 2 | 7 | denovo333 |
| k__Bacteria; p__Proteobacteria; c__Gammaproteobacteria; o__Xanthomonadales; f__Xanthomonadaceae; g__Dokdonella; s__ | 0 | 0 | 0 | 0 | 20 | denovo27115 |
| k__Bacteria; p__Proteobacteria; c__Gammaproteobacteria; o__Xanthomonadales; f__Xanthomonadaceae; g__Lysobacter; s__ | 0 | 0 | 27 | 16 | 22 | denovo17999 |
| k__Bacteria; p__Proteobacteria; c__Gammaproteobacteria; o__Xanthomonadales; f__Xanthomonadaceae; g__Stenotrophomonas; s__ | 0 | 0 | 4 | 29 | 17 | denovo20482 |
| k__Bacteria; p__Proteobacteria; c__Gammaproteobacteria; o__Xanthomonadales; f__Xanthomonadaceae; g__Stenotrophomonas; s__acidaminiphila | 0 | 0 | 0 | 0 | 46 | denovo871 |
| k__Bacteria; p__Proteobacteria; c__Gammaproteobacteria; o__Xanthomonadales; f__Xanthomonadaceae; g__Thermomonas; s__ | 0 | 0 | 1 | 1 | 5 | denovo33080 |
| k__Bacteria; p__Proteobacteria; c__TA18; o__CV90; f__; g__; s__ | 11 | 0 | 0 | 0 | 0 | denovo35702 |
| k__Bacteria; p__Proteobacteria; c__TA18; o__PHOS-HD29; f__; g__; s__ | 38 | 0 | 0 | 0 | 0 | denovo42108 |
| k__Bacteria; p__SAR406; c__AB16; o__; f__; g__; s__ | 0 | 16 | 13 | 0 | 0 | denovo28344 |
| k__Bacteria; p__Spirochaetes; c__[Leptospirae]; o__[Leptospirales]; f__Leptospiraceae; g__Leptonema; s__ | 0 | 0 | 14 | 61 | 0 | denovo17672 |
| k__Bacteria; p__Spirochaetes; c__[Leptospirae]; o__[Leptospirales]; f__Sediment-4; g__; s__ | 0 | 30 | 22 | 6 | 1 | denovo42745 |
| k__Bacteria; p__Spirochaetes; c__MVP-15; o__PL-11B10; f__; g__; s__ | 0 | 0 | 12 | 0 | 0 | denovo14514 |
| k__Bacteria; p__Spirochaetes; c__Spirochaetes | 0 | 67 | 3 | 11 | 1 | denovo6279 |
| k__Bacteria; p__Spirochaetes; c__Spirochaetes; o__; f__; g__; s__ | 0 | 25 | 136 | 10 | 0 | denovo1845 |
| k__Bacteria; p__Spirochaetes; c__Spirochaetes; o__Spirochaetales; f__Spirochaetaceae; g__Spirochaeta; s__ | 1 | 342 | 563 | 26 | 1 | denovo41227 |
| k__Bacteria; p__Spirochaetes; c__Spirochaetes; o__Spirochaetales; f__Spirochaetaceae; g__Treponema | 0 | 0 | 0 | 2 | 3 | denovo37214 |
| k__Bacteria; p__Spirochaetes; c__Spirochaetes; o__Spirochaetales; f__Spirochaetaceae; g__Treponema; s__ | 1 | 0 | 8 | 204 | 85 | denovo44052 |
| k__Bacteria; p__Spirochaetes; c__Spirochaetes; o__Spirochaetales; f__Spirochaetaceae; g__Treponema; s__amylovorum | 0 | 0 | 2 | 13 | 9 | denovo20556 |
| k__Bacteria; p__Spirochaetes; c__Spirochaetes; o__Spirochaetales; f__Spirochaetaceae; g__Treponema; s__socranskii | 0 | 0 | 0 | 2 | 5 | denovo42378 |
| k__Bacteria; p__SR1; c__; o__; f__; g__; s__ | 221 | 1 | 29 | 592 | 377 | denovo7350 |
| k__Bacteria; p__Synergistetes; c__Synergistia; o__Synergistales; f__Dethiosulfovibrionaceae; g__TG5; s__ | 0 | 0 | 1 | 3 | 1 | denovo36054 |
| k__Bacteria; p__Synergistetes; c__Synergistia; o__Synergistales; f__Synergistaceae; g__Cloacibacillus; s__ | 0 | 0 | 0 | 3 | 2 | denovo10609 |
| k__Bacteria; p__Tenericutes | 0 | 16 | 9 | 2 | 9 | denovo29977 |
| k__Bacteria; p__Tenericutes; c__Mollicutes; o__; f__; g__; s__ | 1 | 19 | 10 | 0 | 10 | denovo15196 |
| k__Bacteria; p__Tenericutes; c__Mollicutes; o__Acholeplasmatales; f__; g__; s__ | 0 | 9 | 3 | 0 | 0 | denovo26071 |
| k__Bacteria; p__Tenericutes; c__Mollicutes; o__Anaeroplasmatales; f__Anaeroplasmataceae; g__Anaeroplasma; s__ | 0 | 0 | 60 | 529 | 245 | denovo39169 |
| k__Bacteria; p__Tenericutes; c__Mollicutes; o__Mycoplasmatales; f__Mycoplasmataceae; g__; s__ | 0 | 0 | 9 | 64 | 4 | denovo32109 |
| k__Bacteria; p__Tenericutes; c__Mollicutes; o__Mycoplasmatales; f__Mycoplasmataceae; g__Mycoplasma | 0 | 0 | 0 | 42 | 6 | denovo40111 |
| k__Bacteria; p__Tenericutes; c__Mollicutes; o__Mycoplasmatales; f__Mycoplasmataceae; g__Mycoplasma; s__ | 0 | 0 | 2 | 28 | 11 | denovo41788 |
| k__Bacteria; p__Tenericutes; c__Mollicutes; o__Mycoplasmatales; f__Mycoplasmataceae; g__Mycoplasma; s__muris | 0 | 33 | 21 | 7 | 0 | denovo40821 |
| k__Bacteria; p__Tenericutes; c__Mollicutes; o__Mycoplasmatales; f__Mycoplasmataceae; g__Ureaplasma; s__ | 0 | 0 | 1 | 4 | 3 | denovo36709 |
| k__Bacteria; p__Tenericutes; c__Mollicutes; o__RF39; f__; g__; s__ | 1 | 0 | 10 | 204 | 117 | denovo31994 |
| k__Bacteria; p__TM6; c__SJA-4; o__; f__; g__; s__ | 0 | 0 | 0 | 0 | 7 | denovo39637 |
| k__Bacteria; p__TM7 | 7 | 0 | 0 | 0 | 0 | denovo33733 |
| k__Bacteria; p__TM7; c__TM7-1; o__; f__; g__; s__ | 8 | 0 | 0 | 0 | 0 | denovo30678 |
| k__Bacteria; p__TM7; c__TM7-3; o__; f__; g__; s__ | 0 | 0 | 4 | 55 | 30 | denovo8016 |
| k__Bacteria; p__TM7; c__TM7-3; o__CW040; f__; g__; s__ | 0 | 0 | 0 | 4 | 2 | denovo8726 |
| k__Bacteria; p__TM7; c__TM7-3; o__CW040; f__F16; g__; s__ | 0 | 0 | 1 | 41 | 16 | denovo20484 |
| k__Bacteria; p__TM7; c__TM7-3; o__EW055; f__; g__; s__ | 0 | 0 | 0 | 0 | 9 | denovo16412 |
| k__Bacteria; p__Verrucomicrobia; c__[Spartobacteria]; o__[Chthoniobacterales]; f__[Chthoniobacteraceae]; g__Ellin506; s__ | 0 | 0 | 0 | 12 | 0 | denovo41958 |
| k__Bacteria; p__Verrucomicrobia; c__Opitutae; o__[Cerasicoccales]; f__[Cerasicoccaceae]; g__; s__ | 12 | 0 | 0 | 0 | 8 | denovo14308 |
| k__Bacteria; p__Verrucomicrobia; c__Opitutae; o__Puniceicoccales; f__Puniceicoccaceae | 0 | 0 | 0 | 0 | 9 | denovo30006 |
| k__Bacteria; p__Verrucomicrobia; c__Opitutae; o__Puniceicoccales; f__Puniceicoccaceae; g__Coraliomargarita; s__ | 2 | 3 | 6 | 1 | 2 | denovo29647 |
| k__Bacteria; p__Verrucomicrobia; c__Verruco-5 | 14 | 0 | 0 | 0 | 0 | denovo6126 |
| k__Bacteria; p__Verrucomicrobia; c__Verruco-5; o__MSBL3; f__; g__; s__ | 0 | 317 | 860 | 75 | 0 | denovo38703 |
| k__Bacteria; p__Verrucomicrobia; c__Verruco-5; o__R76-B128; f__; g__; s__ | 3 | 136 | 174 | 61 | 14 | denovo36028 |
| k__Bacteria; p__Verrucomicrobia; c__Verruco-5; o__WCHB1-41 | 0 | 0 | 19 | 0 | 0 | denovo40408 |
| k__Bacteria; p__Verrucomicrobia; c__Verruco-5; o__WCHB1-41; f__RFP12; g__; s__ | 0 | 0 | 0 | 51 | 0 | denovo42706 |
| k__Bacteria; p__Verrucomicrobia; c__Verrucomicrobiae; o__Verrucomicrobiales; f__Verrucomicrobiaceae | 309 | 0 | 1 | 0 | 24 | denovo8608 |
| k__Bacteria; p__Verrucomicrobia; c__Verrucomicrobiae; o__Verrucomicrobiales; f__Verrucomicrobiaceae; g__; s__ | 2230 | 2 | 5 | 0 | 19 | denovo36712 |
| k__Bacteria; p__Verrucomicrobia; c__Verrucomicrobiae; o__Verrucomicrobiales; f__Verrucomicrobiaceae; g__Akkermansia; s__muciniphila | 10 | 12 | 596 | 1274 | 1006 | denovo3063 |
| k__Bacteria; p__Verrucomicrobia; c__Verrucomicrobiae; o__Verrucomicrobiales; f__Verrucomicrobiaceae; g__Luteolibacter; s__ | 0 | 0 | 1 | 0 | 13 | denovo44397 |
| k__Bacteria; p__Verrucomicrobia; c__Verrucomicrobiae; o__Verrucomicrobiales; f__Verrucomicrobiaceae; g__MSBL3; s__ | 365 | 0 | 2 | 0 | 0 | denovo40079 |
| k__Bacteria; p__Verrucomicrobia; c__Verrucomicrobiae; o__Verrucomicrobiales; f__Verrucomicrobiaceae; g__Persicirhabdus; s__ | 64 | 0 | 3 | 8 | 80 | denovo25309 |
| k__Bacteria; p__Verrucomicrobia; c__Verrucomicrobiae; o__Verrucomicrobiales; f__Verrucomicrobiaceae; g__Rubritalea; s__ | 1497 | 8 | 21 | 2 | 40 | denovo13907 |
| k__Bacteria; p__Verrucomicrobia; c__Verrucomicrobiae; o__Verrucomicrobiales; f__Verrucomicrobiaceae; g__Verrucomicrobium; s__ | 103 | 0 | 1 | 0 | 1 | denovo11242 |
| k__Bacteria; p__WPS-2; c__; o__; f__; g__; s__ | 14 | 0 | 0 | 0 | 209 | denovo31448 |
| Unclassified | 0 | 0 | 6 | 0 | 0 | denovo25950 |
