# Supplementary material for: The Purple Sea Urchin Strongylocentrotus purpuratus Demonstrates a Compartmentalization of Gut Bacterial Microbiota, Predictive Functional Attributes, and Taxonomic Co-Occurrence
Source: Microorganisms. 2019 Jan 26;7(2):35. doi: 10.3390/microorganisms7020035 (PMC6406795; doi:10.3390/microorganisms7020035)
Supplement: Supplementary file 1 [file microorganisms-07-00035-s001.zip › Sup_Data_Final/Fig_S3_AB_unweighted_unifrac_r1_edit-final.pdf]

Title: The Purple Sea Urchin *Strongylocentrotus Purpuratus* Demonstrates a Compartmentalization of Gut Bacterial Microbiota, Predictive Functional Attributes, and Taxonomic Co-Occurrence

Authors:
Joseph A. Hakim[1,*], Julie B. Schram[2], Aaron W. E. Galloway[2], Casey D. Morrow[3], Michael R. Crowley[4], Stephen A. Watts[1], and Asim K. Bej[1,*]

Corresponding Author *
Affiliations:
[1]Department of Biology, University of Alabama at Birmingham, 1300 University Blvd., Birmingham, AL 35294, USA; joe21@uab.edu (J.A.H); sawatts@uab.edu (S.A.W.); abej@uab.edu (A.K.B)
[2]Oregon Institute of Marine Biology, University of Oregon, 63466 Boat Basin Rd, Charleston, OR 97420, USA; jschram@uoregon.edu (J.B.S); agallow3@uoregon.edu (A.W.E.G.)
[3]Department of Cell, Developmental and Integrative Biology, University of Alabama at Birmingham, 1918 University Blvd., Birmingham, AL 35294, USA; caseym@uab.edu (C.D.M)
[4]Department of Genetics, Heflin Center Genomics Core, School of Medicine, University of Alabama at Birmingham, 705 South 20th Street, AL 35294 USA; mcrowley@uab.edu (M.R.C)

*****Correspondence: Joseph A. Hakim (joe21@uab.edu) (Primary corresponding author); Asim K. Bej (abej@uab.edu) (Submitting corresponding author); Tel.: +1 (205) 934-9857.

Unweighted Unifrac 3D PCoA

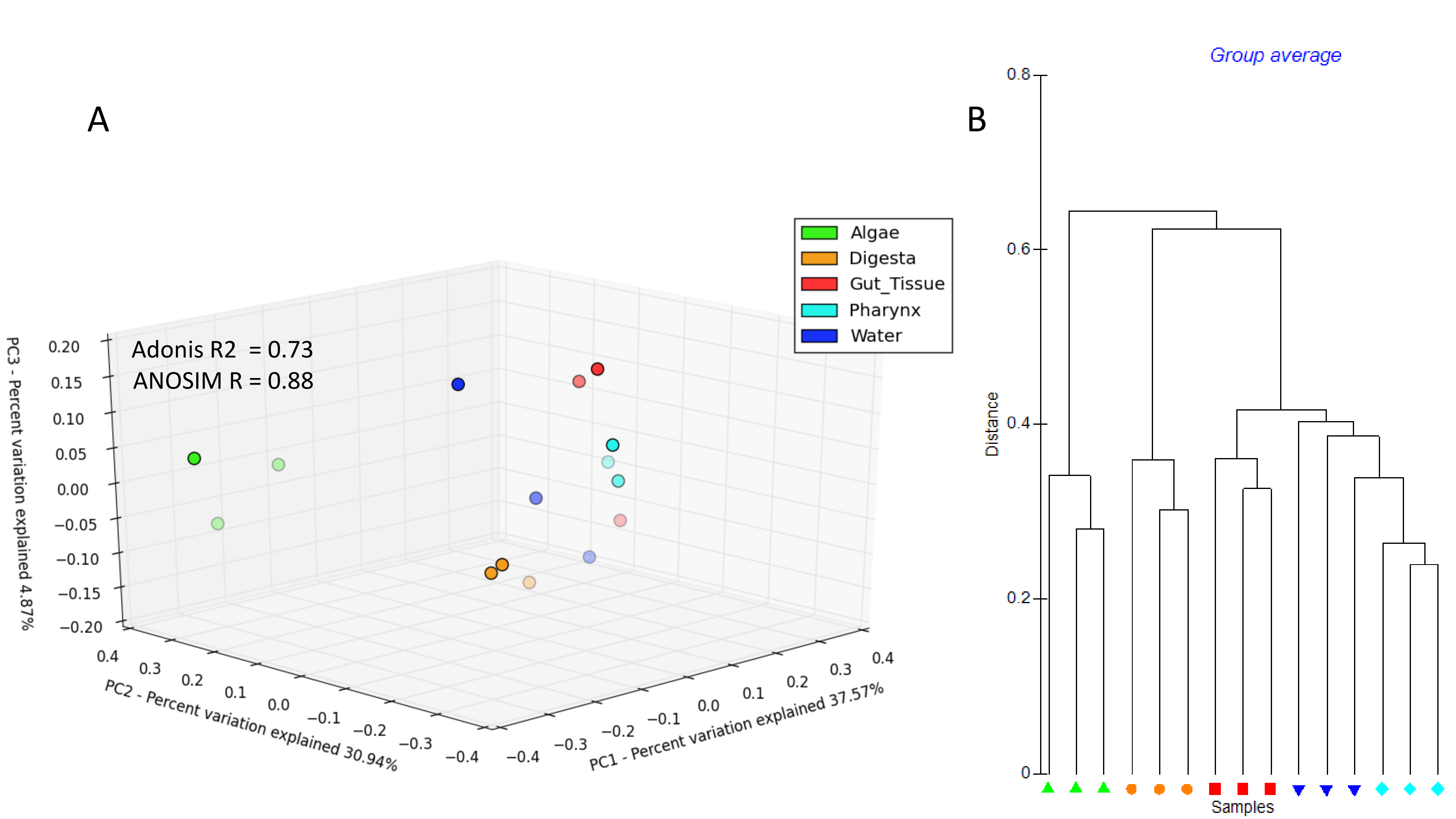

Supplementary Figure 2: 3D principle coordinates analysis (PCoA) plot and dendrogram analysis performed using the unweighted Unifrac metrics calculated for the rarefied OTU table data across all samples of the study. (A) The unweighted Unifrac distances were determined through QIIME (v1.9.1) using the "beta_diversity_through_plots.py" module, and these values were used to construct the 3D PCoA plot using the "PCoA.py" command in PhyloToAST (v1.4.0). The percent variation explained is listed on the X, Y, and Z axes, and the ANOSIM and Adonis measures were calculated between groups using the "compare_categories.py" command in QIIME (v1.9.1) which are listed in the plot. (B) The Unifrac distance values were uploaded into PRIMER-6 (Primer-E Ltd, Plymouth Marine Laboratory, Plymouth UK, v6.1.2), and used to generate the dendrogram using group average clustering. Figure legend corresponding to each sample type is listed to the top right of the PCoA plot.
