# Supplementary material for: The Purple Sea Urchin Strongylocentrotus purpuratus Demonstrates a Compartmentalization of Gut Bacterial Microbiota, Predictive Functional Attributes, and Taxonomic Co-Occurrence
Source: Microorganisms. 2019 Jan 26;7(2):35. doi: 10.3390/microorganisms7020035 (PMC6406795; doi:10.3390/microorganisms7020035)
Supplement: Supplementary file 1 [file microorganisms-07-00035-s001.zip › Sup_Data_Final/Table_S6_KO_LEFSe_Elaborated-final.docx]

**Title:** The Purple Sea Urchin *Strongylocentrotus Purpuratus* Demonstrates a Compartmentalization of Gut Bacterial Microbiota, Predictive Functional Attributes, and Taxonomic Co-Occurrence

**Authors:**

Joseph A. Hakim^1,*^, Julie B. Schram^2^, Aaron W. E. Galloway^2^, Casey D. Morrow^3^, Michael R. Crowley^4^, Stephen A. Watts^1^, and Asim K. Bej^1,*^

**Corresponding Author ^*^**

**Affiliations:**

^1^ Department of Biology, University of Alabama at Birmingham, 1300 University Blvd., Birmingham, AL 35294, USA; joe21@uab.edu (J.A.H); sawatts@uab.edu (S.A.W.); abej@uab.edu (A.K.B)

^2^ Oregon Institute of Marine Biology, University of Oregon, 63466 Boat Basin Rd, Charleston, OR 97420, USA; jschram@uoregon.edu (J.B.S); agallow3@uoregon.edu (A.W.E.G.)

^3^ Department of Cell, Developmental and Integrative Biology, University of Alabama at Birmingham, 1918 University Blvd., Birmingham, AL 35294, USA; caseym@uab.edu (C.D.M)

^4^ Department of Genetics, Heflin Center Genomics Core, School of Medicine, University of Alabama at Birmingham, 705 South 20th Street, AL 35294 USA; mcrowley@uab.edu (M.R.C)

***** Correspondence: Joseph A. Hakim (joe21@uab.edu) (Primary corresponding author); Asim K. Bej (abej@uab.edu) (Submitting corresponding author); Tel.: +1 (205) 934-9857.

**Supplementary Table 6:** Description of each functional category determined through LEFSe analysis (LDA threshold at ± 2.4) of the KEGG Ortholog (KO) Ids determined through PICRUSt (v1.1.2) analysis of the gut tissue and gut digesta microbial communities. Included is each KO Id determined as significant (LDA at ± 2.4) and the accompanying KEGG-Level-1 and 2 designations. Also included is the definition provided through the PICRUSt KO precalculated file based on the KEGG database, and a general function description of the predicted category where possible. The entries are separated based on the sample group of their observed effect size (gut tissue, *n* = 3; gut digesta, *n* = 3).

| **KO Id** | **KEGG-Level-2** | **KEGG-Level-3** | **Definition** | **General Function** | **Reference** |
| --- | --- | --- | --- | --- | --- |
| **Gut Tissue** | | | | | |
| K02484 | Enzyme families;  Signal Transduction | Protein kinases; Two Component System | a two-component system, OmpR family, the sensor kinase | Regulates expression of outer membrane porins for osmoregulation | [1] |
| K07007 | Poorly characterized | General function prediction only | uncharacterized protein |  |  |
| K03559 | Cellular Processes and Signaling | Cell motility and secretion | (exbD) biopolymer transport protein ExbD | Function in ferric ion and biopolymer transport into the cell | [2,3] |
| K07088 | Poorly Characterized | General function prediction only | uncharacterized protein |  |  |
| K02026 | Membrane Transport | Transporters | (ABC.MS.P1) multiple sugar transport system permease proteins | Part of the ABC transporter system involved in Carbohydrate uptake | [4] |
| K00620 | Amino acid metabolism | Arginine and Proline metabolism | (argJ) glutamate N-acetyltransferase / amino-acid N-acetyltransferase | Involved in l-arginine biosynthesis | [5] |
| K07814 | Signal Transduction | Two-Component System | putative two-component system response regulator | Response to environmental stimuli | [6] |
| K00366 | Energy metabolism | Nitrogen metabolism | (nirA) ferredoxin-nitrite reductase | Involved in nitrate uptake and reduction to ammonium in the cell | [7] |
| K01768 | Nucleotide metabolism | Purine metabolism | (E4.6.1.1) adenylate cyclase | Function to synthesize cyclic AMP (cAMP), secondary messenger | [8] |
| K02574 | Energy metabolism | Nitrogen metabolism | (napH) ferredoxin-type protein NapH | Involved in nitrate reductase pathway | [9] |
| K05939 | Lipid metabolism | Fatty acid metabolism;  Glycerophospholipid metabolism | (aas) acyl-[acyl-carrier-protein]-phospholipid O-acyltransferase / long-chain-fatty-acid--[acyl-carrier-protein] ligase | Involved in the acylation of endogenous 2-acyl-GPE in Glycerophospholipid metabolism | [10] |
| K02025 | Membrane transport | Transporters | (ABC.MS.P) multiple sugar transport system permease proteins | Part of the ABC transporter system and involved in carbohydrate uptake | [4] |
| K00123 | Carbohydrate metabolism;  Energy metabolism | Glyoxylate and dicarboxylate metabolism; Methane metabolism | (fdoG, fdhF, fdwA) formate dehydrogenase major subunit | Involved in the metabolism of formate | [11] |
| K01091 | Carbohydrate metabolism | Glyoxylate and dicarboxylate metabolism | (gph) phosphoglycolate phosphatase | Phosphatase required to mitigate DNA damage during oxidative stress | [12] |
| K02435 | Genetic Information Processing | Aminoacyl-tRNA biosynthesis | aspartyl-tRNA(Asn)/glutamyl-tRNA(Gln) amidotransferase subunit C [EC:6.3.5.6 6.3.5.7] | tRNA-mediated transformations of amino acids | [13] |
| K03750 | Metabolism of cofactors and vitamins | Folate biosynthesis | (moeA) molybdopterin molybdotransferase | Involved in cofactor biosynthesis, specifically molybdopterin biosynthesis | [14] |
| K06147 | Membrane transport | Transporters | (ABCB-BAC) ATP-binding cassette, subfamily B, bacterial | ATP‐dependent transmembrane transport | [15] |
| K02573 | Energy metabolism | Nitrogen metabolism | (napG) ferredoxin-type protein NapG | Necessary for Nap-dependent nitrate reduction | [16] |
| K12506 | Metabolism of terpenoids and polyketides | Terpenoid backbone biosynthesis | (ispDF) 2-C-methyl-D-erythritol 4-phosphate cytidylyltransferase / 2-C-methyl-D-erythritol 2,4-cyclodiphosphate synthase | Important in evalonate-independent biosynthesis of isoprenoids | [17] |
| K01952 | Nucleotide metabolism | Purine metabolism | (purL, PFAS) phosphoribosylformylglycinamidine synthase | Functions in the biosynthetic pathway of purines. | [18] |
| K02434 | Genetic Information Processing | Aminoacyl-tRNA biosynthesis | (gatB, PET112) aspartyl-tRNA(Asn)/glutamyl-tRNA(Gln) amidotransferase subunit B | Involved in the tRNA-dependent conversion of non-cognate amino acids | [19] |
| K02050 | Membrane transport | ABC Transporters; Transporters | sulfonate/nitrate/taurine transport system permease protein | Sulfonate transporter, Nitrate, nitrite, and cyanate uptake transporter (NitT), Taurine uptake transporter (TauT) | [20] |
| K07005 | Poorly characterized | General function prediction only | uncharacterized protein |  |  |
| K01644 | Carbohydrate Metabolism; Signal transduction | Citrate cycle (TCA cycle); Two-component system | (citE) citrate lyase subunit beta / citryl-CoA lyase | involved in carbon-carbon lyase activity mediating citryl-CoA to acetyl-CoA and oxaloacetate conversion as part of the reverse TCA cycle | [21] |
| K02006 | Membrane transport | ABC transporters; Transporters | (cbiO) cobalt/nickel transport system ATP-binding protein | Protein necessary in cobalt cofactor metal uptake | [22] |
| K04758 | Cellular Processes and Signaling | Other transporters | (feoA) ferrous iron transport protein A | Necessary for Iron uptake into the cell | [23] |
| **Gut Digesta** | | | | | |
| K07566 | Translation | Transfer RNA biogenesis | (tsaC, rimN, SUA5) threonylcarbamoyladenylate synthase | Proteins necessary in the formation of threonylcarbamoyl of adenosine in the formation of tRNA | [24,25] |
| K12524 | Amino acid metabolism | Glycine, serine and threonine metabolism | (thrA) bifunctional aspartokinase / homoserine dehydrogenase 1 | Involved in the biosynthesis of threonine | [26] |
| K06149 | Cellular processes and signaling | Signal transduction mechanisms | (uspA) universal stress protein A | Expressed during stress and starvation | [27] |
| K05365 | Glycan biosynthesis and metabolism | Glycosyltransferases; Peptidoglycan biosynthesis | (mrcB) penicillin-binding protein 1B | Cell wall formation and synthesis cross-linked peptidoglycan synthesis from lipid intermediates | [28] |
| K02477 | Signal transduction | Two-component system | two-component system, LytTR family, response regulator | Involved in autolysis regulation, found in combination with other sensor domains, and possesses DNA binding domain | [29] |
| K06142 | Cellular Processes and Signaling | Membrane and intracellular structural molecules | (hlpA, ompH) outer membrane protein | Response to environmental cues such as cell density, carbon availability and barometric pressure | [30] |
| K09808 | Membrane transport | ABC Transporter; Transporters | lipoprotein-releasing system permease protein | The release of lipoprotein from the cell membrane | [31,32] |
| K01089 | Amino acid metabolism | Histidine metabolism | (hisB) imidazoleglycerol-phosphate dehydratase / histidinol-phosphatase | Involved in the synthesis of histidine | [33] |
| K02548 | Metabolism of cofactors and vitamins; Metabolism of Terpenoids and Polyketides | Ubiquinone and other terpenoid-quinone biosynthesis; Prenyltransferases | (menA) 1,4-dihydroxy-2-naphthoate octaprenyltransferase | Involved in the production of Menaquinone (MK; vitamin K2) from demethylmenaquinone | [34] |
| K07090 | Poorly Characterized | General function prediction only | uncharacterized protein |  |  |
| K03704 | Transcription | Transcription factors | cold shock protein (beta-ribbon, CspA family) | Destabilizes secondary structures in mRNAs in response to cold stress | [35] |
| K01625 | Carbohydrate metabolism; Amino Acid Metabolism | Pentose phosphate pathway;  Glyoxylate and dicarboxylate metabolism; Pentose and glucuronate interconversions; Arginine and proline metabolism | 2-dehydro-3-deoxyphosphogluconate aldolase / (4S)-4-hydroxy-2-oxoglutarate aldolase | Aldolase involved in carbohydrate metabolism | [36.37] |
| K07025 | Poorly characterized | General function prediction only | putative hydrolase of the HAD superfamily | Large and diverse superfamily that is involved in xenobiotics and metabolic byproduct detoxification | [38] |
| K06177 | Translation | Ribosome Biogenesis | tRNA pseudouridine32 synthase / 23S rRNA pseudouridine746 synthase; ribosomal large subunit pseudouridine synthase A | Involved in tRNA and 23S rRNA synthesis | [39] |

**References**

1. Egger, L.A.; Park, H.; Inouye, M. Signal transduction via the histidyl‐aspartyl phosphorelay. *Genes Cells*. **1997**, 2, 167-184. DOI: 10.1046/j.1365-2443.1997.d01-311.x.
2. Eick-Helmerich, K.; Braun, V. Import of biopolymers into *Escherichia coli*: nucleotide sequences of the exbB and exbD genes are homologous to those of the tolQ and tolR genes, respectively. *J. Bacteriol.* **1989**, 171**,** 5117-5126. DOI: 10.1128/jb.171.9.5117-5126.1989.
3. Wiggerich, H.G.; Klauke, B.; Köplin, R.; Priefer, U.B.; Pühler, A. Unusual structure of the tonB-exb DNA region of *Xanthomonas campestris* pv. campestris: tonB, exbB, and exbD1 are essential for ferric iron uptake, but exbD2 is not. *J. Bacteriol.* **1997**, 179**,** 7103-7110. DOI: 10.1128/jb.179.22.7103-7110.1997.
4. Schneider, E. ABC transporters catalyzing carbohydrate uptake. *Res. Microbiol.* **2001**, 152**,** 303-310. DOI: 10.1016/s0923-2508(01)01201-3.
5. Marc, F.; Weigel, P.; Legrain, C.; Almeras, Y.; Santrot, M.; Glansdorff, N.; Sakanyan, V. Characterization and kinetic mechanism of mono‐and bifunctional ornithine acetyltransferases from thermophilic microorganisms. *FEBS J.* **2000**, 267**,** 5217-5226. DOI: 10.1046/j.1432-1327.2000.01593.x.
6. Hoch, J.A. Two-component and phosphorelay signal transduction. *Curr. Opin. Microbiol.* **2000**, 3**,** 165-170. DOI: 10.1016/s1369-5274(00)00070-9.
7. Frías, J.E.; Flores, E. Induction of the nitrate assimilation nirA operon and protein-protein interactions in the maturation of nitrate and nitrite reductases in the cyanobacterium *Anabaena* sp. strain PCC 7120. *J. Bacteriol.* **2015**, 197**,** 2442-2452. DOI: 10.1128/jb.00198-15.
8. Baker, D.A.; Kelly, J.M. Structure, function and evolution of microbial adenylyl and guanylyl cyclases. *Mol. Microbiol.* **2004**, 52**,** 1229-1242. DOI: 10.1111/j.1365-2958.2004.04067.x.
9. Kern, M.; Simon, J. Periplasmic nitrate reduction in *Wolinella succinogenes*: cytoplasmic NapF facilitates NapA maturation and requires the menaquinol dehydrogenase NapH for membrane attachment. *Microbiology.* **2009**, 155**,** 2784-2794. DOI: 10.1099/mic.0.029983-0.
10. Jackowski, S.; Jackson, P.D.; Rock, C.O. Sequence and function of the aas gene in *Escherichia coli*. *J. Biol. Chem.* **1994**, 269**,** 2921-2928.
11. Abaibou, H.; Pommier, J.; Benoit, S.; Giordano, G.; Mandrand-Berthelot, M.A. Expression and characterization of the *Escherichia coli* fdo locus and a possible physiological role for aerobic formate dehydrogenase. *J. Bacteriol.* **1995,** 177**,** 7141-7149. DOI: 10.1128/jb.177.24.7141-7149.1995.
12. Pellicer, M.T.; Nunez, M.F.; Aguilar, J.; Badia, J.; Baldoma, L. Role of 2-phosphoglycolate phosphatase of *Escherichia coli* in metabolism of the 2-phosphoglycolate formed in DNA repair. *J. Bacteriol.* **2003**, 185**,** 5815-5821. DOI: 10.1128/jb.185.19.5815-5821.2003.
13. Ibba, M.; Söll, D. Aminoacyl-tRNA synthesis. *‎Annu. Rev. Biochem.* **2000**, 69**,** 617-650. DOI: 10.1093/embo-reports/kve095.
14. Nichols, J.D.; Rajagopalan, K. In vitro molybdenum ligation to molybdopterin using purified components. *J. Biol. Chem.* **2005**, 280**,** 7817-7822. DOI: 10.1074/jbc.m413783200.
15. Linton, K.J.; Higgins, C.F. The *Escherichia coli* ATP‐binding cassette (ABC) proteins. *Mol. Microbiol*. **1998**, 28**,** 5-13. DOI: 10.1046/j.1365-2958.1998.00764.x.
16. Brondijk, T.H.C.; Nilavongse, A.; Filenko, N.; Richardson, D.J. NapGH components of the periplasmic nitrate reductase of *Escherichia coli* K-12: location, topology and physiological roles in quinol oxidation and redox balancing. *Biochem. J.* **2004**, 379**,** 47-55. DOI: 10.1042/bj20031115.
17. Jin, Y.; Liu, Z.; Li, Y.; Liu, W.; Tao, Y.; Wang, G. A structural and functional study on the 2-C-methyl-d-erythritol-4-phosphate cytidyltransferase (IspD) from *Bacillus subtilis*. *‎Sci. Rep.* **2016**, 6**,** 36379. DOI: 10.1038/srep36379.
18. Patterson, D.; Bleskan, J.; Gardiner, K.; Bowersox, J. Human phosphoribosylformylglycineamide amidotransferase (FGARAT): regional mapping, complete coding sequence, isolation of a functional genomic clone, and DNA sequence analysis. *Gene.* **1999**, 239**,** 381-391. DOI: 10.1016/s0378-1119(99)00378-9.
19. Sheppard, K.; Yuan, J.; Hohn, M.J.; Jester, B.; Devine, K.M.; Söll, D. From one amino acid to another: tRNA-dependent amino acid biosynthesis. *Nucleic Acids Res.* **2008**, 36**,** 1813-1825. DOI: 10.1093/nar/gkn015.
20. Saier Jr, M.H. Families of transmembrane transporters selective for amino acids and their derivatives. *Microbiology.* **2000**, 146**,** 1775-1795. DOI: 10.1099/00221287-146-8-1775.
21. Hallam, S.J.; Mincer, T.J.; Schleper, C.; Preston, C.M.; Roberts, K.; Richardson, P.M.; Delong, E.F. Pathways of carbon assimilation and ammonia oxidation suggested by environmental genomic analyses of marine Crenarchaeota. *PLoS Biol.* **2006**, 4**,** e95. DOI: 10.1371/journal.pbio.0040095.
22. Rodionov, D.A.; Hebbeln, P.; Gelfand, M.S.; Eitinger, T. Comparative and functional genomic analysis of prokaryotic nickel and cobalt uptake transporters: evidence for a novel group of ATP-binding cassette transporters. *J. Bacteriol.* **2006**, 188**,** 317-327. DOI: 10.1128/jb.188.1.317-327.2006.
23. Lau, C.K.; Krewulak, K.D.; Vogel, H.J. Bacterial ferrous iron transport: the Feo system. *FEMS Microbiol. Rev.* **2015**, 40**,** 273-298. DOI: 10.1093/femsre/fuv049.
24. El Yacoubi, B.; Lyons, B.; Cruz, Y.; Reddy, R.; Nordin, B.; Agnelli, F.; Williamson, J.R.; Schimmel, P.; Swairjo, M.A.; De Crecy-Lagard, V. The universal YrdC/Sua5 family is required for the formation of threonylcarbamoyladenosine in tRNA. *Nucleic Acids Res.* **2009**, 37**,** 2894-2909. DOI: 10.1093/nar/gkp152.
25. Harris, K.A.; Bobay, B.G.; Sarachan, K.L.; Sims, A.F.; Bilbille, Y.; Deutsch, C.; Iwata-Reuyl, D.; Agris, P.F. NMR-based structural analysis of threonylcarbamoyl-AMP synthase and its substrate interactions. *J. Biol. Chem.* **2015**, 290**,** 20032-20043. DOI: 10.1074/jbc.m114.631242.
26. Katinka, M.; Cossart, P.; Sibilli, L.; Saint-Girons, I.; Chalvignac, M.; Le Bras, G.; Cohen, G.; Yaniv, M. Nucleotide sequence of the thrA gene of *Escherichia coli*. *Proc. Natl. Acad. Sci. U.S.A.* **1980**, 77**,** 5730-5733. DOI: 10.1073/pnas.77.10.5730.
27. Nyström, T.; Neidhardt, F.C. Expression and role of the universal stress protein, UspA, of *Escherichia coli* during growth arrest. *Mol. Microbiol.* **1994**, 11**,** 537-544. DOI: 10.1111/j.1365-2958.1994.tb00334.x.
28. Typas, A.; Banzhaf, M.; Van Saparoea, B.V.D.B.; Verheul, J.; Biboy, J.; Nichols, R.J.; Zietek, M.; Beilharz, K.; Kannenberg, K.; Von Rechenberg, M.; Breukink, E.; den Blaauwen, T.; Gross C.A; Vollmer, W. Regulation of peptidoglycan synthesis by outer-membrane proteins. *Cell.* **2010**, 143**,** 1097-1109. DOI: 10.1016/j.cell.2010.11.038.
29. Nikolskaya, A.N.; Galperin, M.Y. A novel type of conserved DNA-binding domain in the transcriptional regulators of the AlgR/AgrA/LytR family. *Nucleic Acids Res.* **2002**, 30**,** 2453-2459. DOI: 10.1093/nar/30.11.2453.
30. Bartlett, D.H.; Welch, T.J. OmpH gene expression is regulated by multiple environmental cues in addition to high pressure in the deep-sea bacterium *Photobacterium* species strain SS9. *J. Bacteriol.* **1995**, 177**,** 1008-1016. DOI: 10.1128/jb.177.4.1008-1016.1995.
31. Fitzgerald, S.N.; Foster, T.J. Molecular analysis of the tagF gene, encoding CDP-glycerol: poly (glycerophosphate) glycerophosphotransferase of *Staphylococcus epidermidis* ATCC 14990. *J. Bacteriol.* **2000**, 182**,** 1046-1052. DOI: 10.1128/jb.182.4.1046-1052.2000.
32. Yakushi, T.; Masuda, K.; Narita, S.I.; Matsuyama, S.I.; Tokuda, H. A new ABC transporter mediating the detachment of lipid-modified proteins from membranes. *Nat. Cell Biol.* **2000**, 2**,** 212. DOI: 10.1038/35008635.
33. Chiariotti, L.; Nappo, A.G.; Carlomagno, M.S.; Bruni, C.B. Gene structure in the histidine operon of *Escherichia coli*. *Mol. Gen. Genet,* **1986**, 202**,** 42-47. DOI: 10.1007/bf00330514.
34. Suvarna, K.; Stevenson, D.; Meganathan, R.; Hudspeth, M. Menaquinone (Vitamin K2) Biosynthesis: Localization and Characterization of the menA Gene from *Escherichia coli*. *J. Bacteriol.* **1998**, 180**,** 2782-2787. DOI: 10.1016/0378-1119(95)00721-0.
35. Bae, W.; Jones, P.G.; Inouye, M. CspA, the major cold shock protein of *Escherichia coli*, negatively regulates its own gene expression. *J. Bacteriol.* **1997**, 179**,** 7081-7088. DOI: 10.1128/jb.179.22.7081-7088.1997.
36. Patil, R.V.; and Dekker, E.E. Cloning, nucleotide sequence, overexpression, and inactivation of the *Escherichia coli* 2-keto-4-hydroxyglutarate aldolase gene. *J. Bacteriol.* **1992**,174**,** 102-107. DOI: 10.1128/jb.174.1.102-107.1992.
37. Holmes, M.L.; Dyall‐Smith, M.L. Sequence and expression of a halobacterial β‐galactosidase gene. *Mol. Microbiol.* **2000**, 36**,** 114-122. DOI: 10.1046/j.1365-2958.2000.01832.x.
38. Koonin, E.V.; Tatusov, R.L. Computer analysis of bacterial haloacid dehalogenases defines a large superfamily of hydrolases with diverse specificity: application of an iterative approach to database search. *J. Mol. Biol.* **1994**, 244**,** 125-132. DOI: 10.1006/jmbi.1994.1711.
39. Raychaudhuri, S.; Niu, L.; Conrad, J.; Lane, B.G.; and Ofengand, J. Functional Effect of Deletion and Mutation of the *Escherichia coli* Ribosomal RNA and tRNA Pseudouridine Synthase RluA. *J. Biol. Chem.* **1999**, 274**,** 18880-18886. DOI: 10.1074/jbc.274.27.18880.
